# Secular change in orogenic duration reveals alternating tectonic regimes during the Archaean-Proterozoic

**DOI:** 10.1038/s41467-026-76530-3

**Published:** 2026-08-11

**Authors:** Lei Zou, Guangyu Huang, Jinghui Guo, Peter A. Cawood, Jia-Hui Liu, Pinghua Liu, Shujuan Jiao, Xiao-Ping Xia, Huijuan Li, Jun-Bo Zhang, Meiyun Huang, Lifei Zhang, Guochun Zhao

**Affiliations:** 1 Hubei Key Laboratory of Petroleum Geochemistry and Environment, YU-CUGW Joint Research Center on Deep Earth and Surface Dynamic Coupling, College of Resources and Environment, Yangtze University, Wuhan, China; 2 State Key Laboratory of Lithospheric and Environmental Coevolution, Institute of Geology and Geophysics, Chinese Academy of Sciences, Beijing, China; 3 Institute of Geology, Chinese Academy of Geological Sciences, Beijing, China; 4 NWU-HKU Joint Centre of Earth and Planetary Sciences, Department of Earth and Planetary Sciences, The University of Hong Kong, Pokfulam Road, Hong Kong, China; 5 School of Earth, Atmosphere & Environment, Monash University, Melbourne, VIC Australia; 6 Center for High Pressure Science & Technology Advanced Research, Beijing, China; 7 Key Laboratory of Orogenic Belts and Crustal Evolution, MOE, School of Earth and Space Sciences, Peking University, Beijing, China; 8 State Key Laboratory of Continental Evolution and Early Life, NWU-HKU Joint Centre of Earth and Planetary Sciences, Department of Geology, Northwest University, Xi’an, China

**Keywords:** Precambrian geology, Geodynamics

## Abstract

Geological constraints and modelling results reveal major yet poorly understood changes in Earth’s tectonic regimes during the late Archaean and Proterozoic. These changes are associated with supercontinent cycles, a primary archive of lithosphere and convecting mantle behaviour, and impact orogen duration and thermal state. However, the lack of precise data on orogen duration during Columbia assembly limits our understanding of the long-term tectonic evolution and comparison with younger orogens. Through in-situ garnet Lu–Hf and monazite microdomain U–Pb geochronology, here we constrain a 220 Myr hot collisional orogen during Columbia assembly, significantly longer than contemporary cold orogens (110–160 Myr). Secular changes in collisional orogen duration indicate a non-monotonic trend from Neoarchaean to Neoproterozoic. This trend implies enhanced plate rigidity and mobility during Columbia assembly, followed by a marked decline during Rodinia assembly, consistent with inferred plate velocity proxies. These findings support evolving tectonic regimes alternating between sluggish lid and more active plate tectonics during the Neoarchaean-Proterozoic.

## Abstract (web summary)

Secular change in orogenic duration reveals a non-monotonic evolution from the Neoarchaean to Neoproterozoic, indicating alternating tectonic regimes between a sluggish lid and active plate tectonics.

## Introduction

Earth’s tectonic regime reflects its dominant mechanism of internal heat dissipation^1^. Combined geological data and modelling results indicate that tectonic regime responds and evolves to changes in mantle viscosity and temperature, lithospheric strength, and the nature of mantle-lithosphere coupling during Earth’s evolution^1–6^. However, the type and duration of tectonic regimes that have operated during Earth’s history, particularly during the Neoarchaean-Proterozoic, remain a source of intense debate. Some models propose that secular mantle cooling progressively enhanced lithospheric rigidity and mobility^7–10^, culminating in modern plate tectonics, while others suggest that tectonic regimes alternated spatially or temporally between distinct end-members^5,11,12^.

**Fig. 1:**
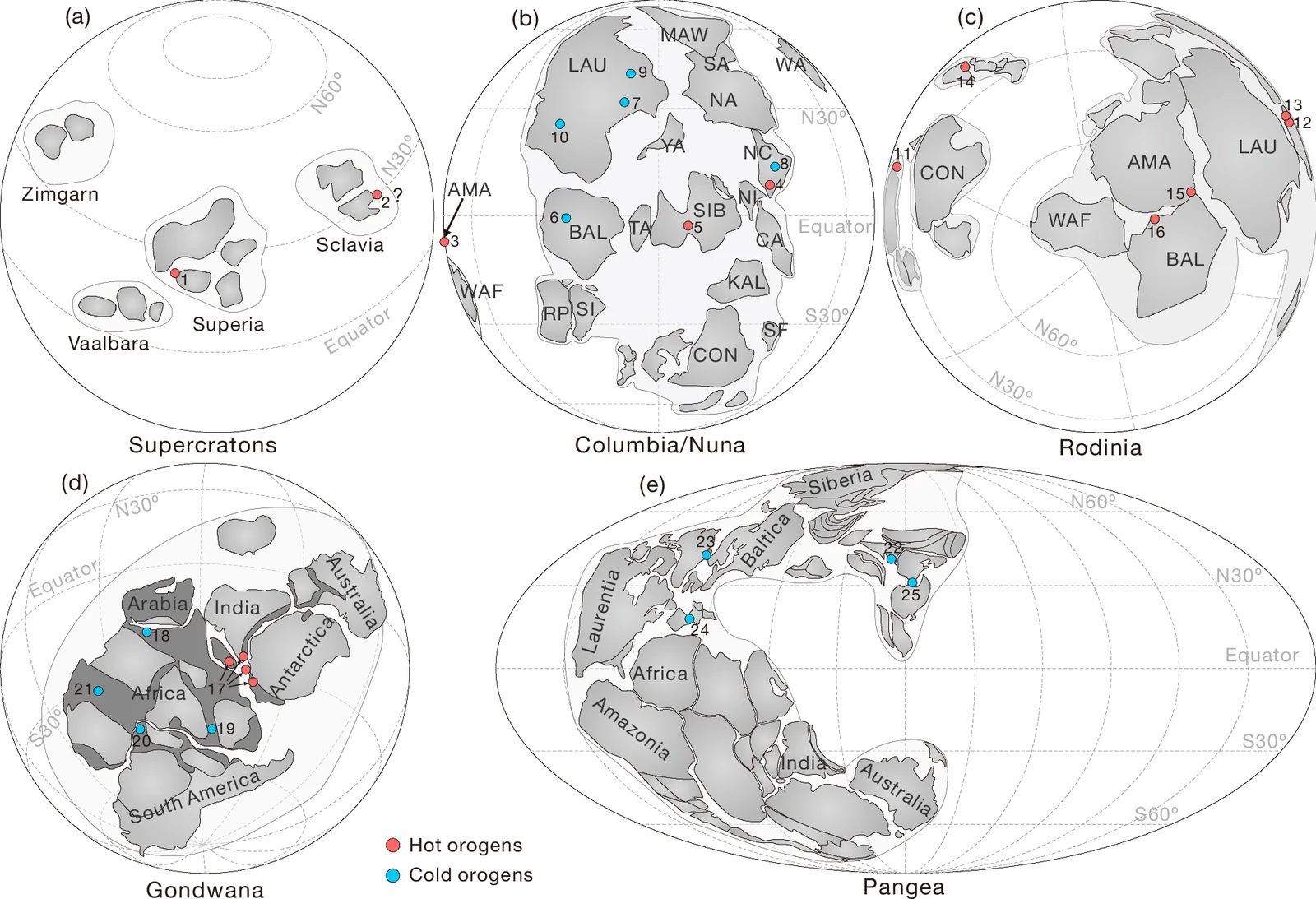
Reconstruction of supercontinents/supercratons/megacontinents from Neoarchaean to Phanerozoic. **a** Neoarchaean Supercratons. This map is adapted and colourised from ref. ^97^, with permission from Elsevier. **b**, **c** Palaeoproterozoic Columbia/Nuna and Neoproterozoic Rodinia supercontinents. Both maps are adapted and colourised from ref. ^78^, with permission from Elsevier. **d** The Cambrian Gondwana megacontinent. This map is adapted and colourised from ref. ^98^, with permission from the Geological Society of America. **e** The Triassic Pangea supercontinent. This map is adapted and colourised from ref. ^99^, with permission from Elsevier. LAU Laurentia, AMA Amazonia, SF São Francisco, BAL Baltica, RP Rayner Province, MAW Mawson, SA South Australia, NA North Australia, WA West Australia, YA Yangtze, CA Cathaysia, NC North China, SIB Siberia, TA Tarim, SI South India, NI North India, CON Congo, KAL Kalahari, WAF West Africa. The numbers in the figure represent the orogenic belt numbers compiled in this study, and each number corresponds to that in Supplementary Data 5.

Supercontinent/supercraton cycle (Fig. 1), characterised by planetary-scale and horizontal tectonic motion, provides a long-term record of lithospheric behaviour (e.g., rigidity and mobility)^13^. The temporal and thermal evolution of the lithosphere can provide key insights into Earth’s tectonic regimes^14,15^. Geological records and geodynamic modelling indicate that Precambrian (Neoarchaean-Proterozoic) orogenic belts are distinct from Phanerozoic orogenic belts^9,16^. Specifically, Precambrian belts have slow rates of exhumation constrained by both thermochronology and diffusion chronometry^17^, a predominance of high (e.g., ultrahigh-temperature [UHT; *T*_max_ ≥ 900 °C] granulites) and moderate (e.g., high-pressure [HP] granulites) d*T*/d*P* metamorphism^18,19^, and the widespread development of large-scale (> 500 km^2^; based on the temperature mapping results) and sustained (UHT duration ≥ 50 Myr) UHT metamorphism^20,21^, pointing to a markedly contrasting lithospheric character and tectonic regime compared to Phanerozoic counterparts^8,22–24^. Under such extreme UHT conditions during Precambrian hot orogeny, mineral reactions tend to proceed to completion, resulting in extensive major-element re-equilibration^25,26^ and typically erase petrofabric, mineral-chemical, and geochronological evidence of the early prograde path^20,21^. Consequently, high-precision age constraints on the initial collision stage and duration of Precambrian hot orogens are notably lacking, especially for the critical Columbia/Nuna assembly (ca. 2.0–1.8 Ga). This gap fundamentally limits our understanding of the long-term evolution of the supercontinent cycles and Earth’s tectonic regimes.

Recent studies suggest that certain trace elements in garnet (e.g., heavy rare earth elements [HREE], Ti, and Sc) diffuse remarkably slowly^27,28^, potentially preserving compositional zoning related to prograde growth and thus providing insights into the early thermal history^29,30^. Furthermore, combined with the cutting-edge in situ garnet Lu–Hf dating technique, the notably high closure temperature (e.g., UHT conditions) of the garnet Lu–Hf system enables the direct acquisition of early prograde metamorphic age information from UHT rocks^31–33^. Concurrently, porphyroblastic garnet can encapsulate prograde-stage accessory minerals (e.g., monazite, zircon) during its growth, shielding them from subsequent UHT overprinting and thereby preserving geochronological information of the prograde evolution^34,35^. These developments offer a promising avenue for reconstructing the complete UHT metamorphic evolution and constraining the duration and thermal state of Precambrian hot orogens.

The North China Craton (NCC), as an important component of the Columbia/Nuna supercontinent (Fig. 1b; ref. ^36^), formed through the amalgamation of several Archaean blocks along Palaeoproterozoic orogenic belts at ca. 1.95–1.8 Ga^37^. In particular, the Khondalite Belt (Supplementary Fig. 1), situated in the western part of the NCC, is characterised by widespread high and moderate d*T*/d*P* granulites containing sapphirine-bearing mineral assemblages (e.g., ref. ^38,39^). Its > 3000 km^2^ (based on the mapping of Zr-in-rutile thermometer) UHT metamorphic terranes and protracted (ca. 50 Myr) UHT thermal regimes^40,41^ represent a typical hot orogen, serving as a representative research area for constraining its Palaeoproterozoic tectonic regime. However, the eastern Khondalite Belt was strongly affected by intense mantle-derived magmatism, experiencing multiphase cooling, reheating, and UHT events^40,42^. Consequently, it fails to reflect the typical erosional exhumation processes of a hot orogen. In contrast, the western Khondalite Belt remained largely unaffected by such magmatic activity, recording a simple clockwise pressure–temperature (*P*–*T*) path, with an early stage involving decompression and heating from HP granulite facies to UHT conditions, followed by near-isobaric cooling^43–46^. These well-preserved records not only provide critical insights into the early prograde evolution of UHT granulites but also offer robust constraints on the duration and thermal state of Palaeoproterozoic hot orogenesis.

To constrain the duration of collisional orogenesis, we combined textural–chemical zoning analysis of garnet with in situ garnet Lu–Hf dating and U–Pb geochronology of monazite inclusions for the UHT granulite from the western Khondalite Belt (Supplementary Fig. 1b, c). This multi-method approach precisely identifies prograde garnet growth and tightly constrains the complete collisional orogenic evolution. Integrated with the supercontinent cycle dataset and one-dimensional thermal modelling, our results reveal a non-monotonic evolution in collisional orogenic duration and shed light on the evolutionary styles of the Earth’s tectonic regimes during the Neoarchaean-Proterozoic.

## Results and discussion

### Palaeoproterozoic ca. 220 Myr collisional orogenic duration

**Fig. 2:**
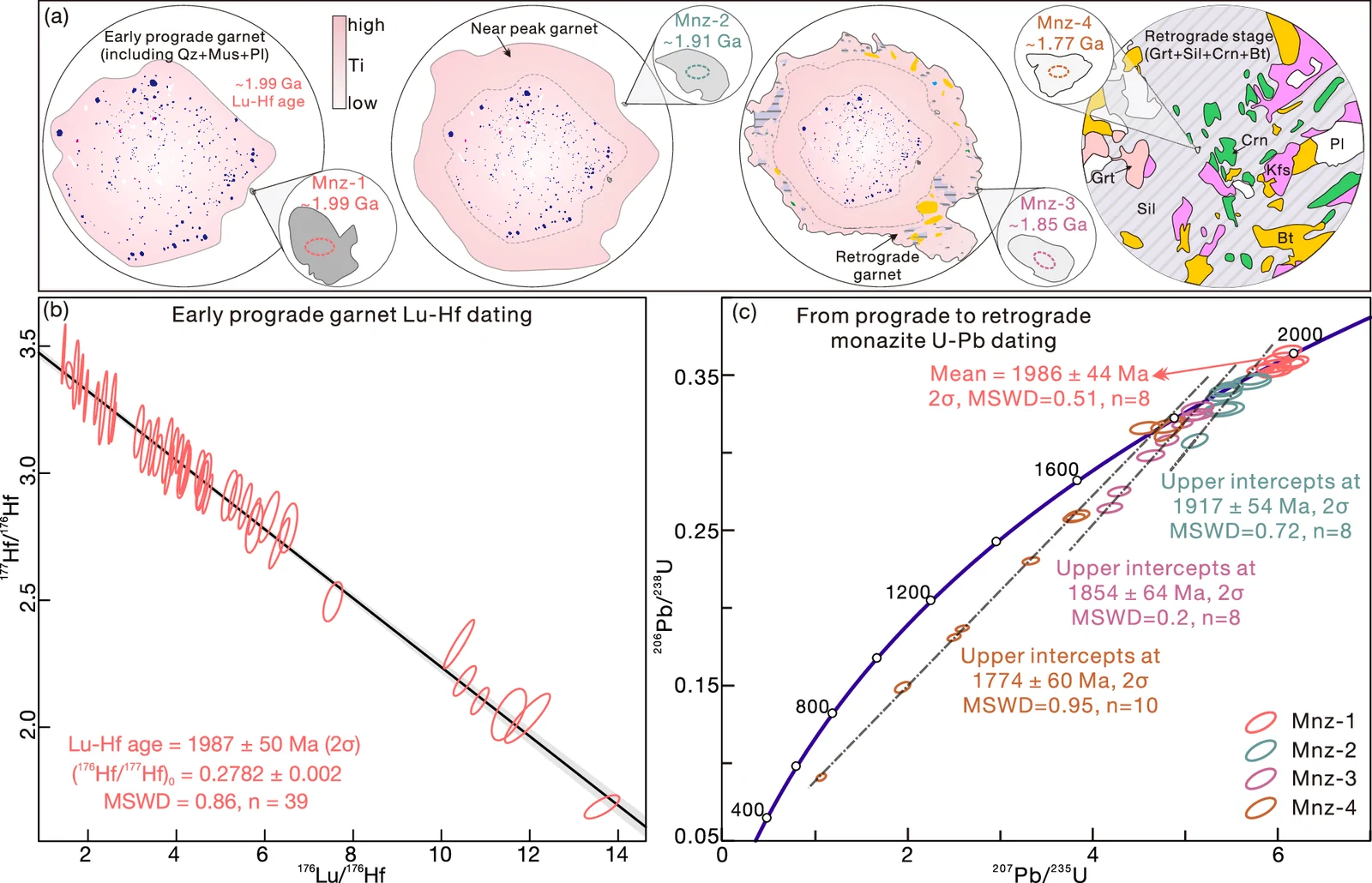
Structural relationships and geochronological results between garnet and monazite. **a** Structural relationships among the garnet core-mantle-rim and four-type monazite microdomains. **b** Lu–Hf isochron plots for porphyroblastic garnet core from the pelitic granulite samples (the raw data are presented in Supplementary Data 2). **c** Concordia diagram of the four-type monazite microdomains U–Pb dating (the raw data are presented in Supplementary Data 4). Mineral abbreviations: Mnz monazite, Qz quartz, Mus muscovite, Pl plagioclase, Grt garnet, Sil sillimanite, Crn corundum, Bt biotite, Kfs K-feldspar.

Based on extensive screening and detailed structural and trace element analysis (Supplementary Data 1 and Supplementary Figs. 2–4), we have identified a three-stage growth (including early prograde stage [garnet core], near peak stage [garnet mantle], and retrograde cooling stage [garnet rim and fine grain]) of garnet in UHT granulite samples (detailed description see Supplementary material). The latest garnet in situ Lu–Hf geochronology method yielded a garnet core age of 1987 ± 50 Ma (2σ, MSWD = 0.86, *n* = 39) (Fig. 2a, b and Supplementary Data 2). This age is interpreted as representing the timing of the early prograde stage (near initial collision) due to slow diffusion rates^32,33,47^ for the Lu–Hf system (ca. 950–1000 °C closure temperature, considering garnet grain radius of 2–4 mm; Supplementary Fig. 5d). The peritectic garnet mantle, with a Lu–Hf age of 1957 ± 152 Ma (Supplementary Fig. 6a; the large error caused by the low Lu contents, which provides key mineral chemical evidence distinguishing the garnet mantle from the core), is interpreted as potentially corresponding to the HP granulite-facies stage (ca. 1.96–1.95 Ga^38,44,48,49^; peak collision) in the western Khondalite Belt.

Monazites from the UHT granulite samples occur in four microdomains (Fig. 2a and Supplementary Fig. 6–8) that record metamorphic ages from early prograde to retrograde cooling stage (Supplementary Data 3–4). First-domain monazites (Mnz-1), texturally occurring as inclusions in porphyroblastic garnet core-mantle transitions (Fig. 2a and Supplementary Fig. 7a, b), exhibit high Y + HREE concentrations (Supplementary Fig. 6b, c), indicating that their growth may commence slightly earlier than the garnet core. Nevertheless, their weighted mean age (1986 ± 44 Ma and 1977 ± 16 Ma; Fig. 2c and Supplementary Fig. 8b) is consistent within error with the Lu–Hf age from garnet core. Second domain monazites (Mnz-2) at garnet rims or overgrowing Mnz-1 (Fig. 2a and Supplementary Fig. 7b) show depleted Y + HREE signatures (Supplementary Fig. 6b, c) and give an upper-intercept age of 1917 ± 54 Ma (Fig. 2c). This age aligns with regional UHT events at ca. 1.92–1.91 Ga^39,45,50,51^. Third and fourth domain monazites (Mnz-3 and Mnz-4) collectively record the late-stage cooling (1.85–1.77 Ga; Fig. 2c), evidenced by the extreme Y and HREE depletion, the textural entrapment within outermost garnets or in retrograde minerals (sillimanite or biotite) (Fig. 2a, Supplementary Figs. 6b, c and 7c, d).

Collectively, combining our multi-chronometer framework and the previous metamorphic (a single UHT event^43,52^) and geochronological data, reveals a collisional orogenic duration of ca. 220 Myr for the western Khondalite Belt during the Palaeoproterozoic. Collision commenced at ca. 1.99–1.98 Ga (this study), progressed to peak collision by ca. 1.96–1.95 Ga^38,44,48,49^, culminating in the thermal peak at ca. 1.92–1.91 Ga (e.g., ref. ^45,50,53,54^ and this study) and concluded with gradual cooling until ca. 1.8–1.77 Ga (e.g., ref. ^43–45^ and this study). These critical ages constrain the duration and thermal state of a Palaeoproterozoic hot orogen and provide a robust foundation for deciphering the Earth’s long-term thermal and tectonic regime evolutionary styles.

### Non-monotonic evolution of collisional orogenic duration

Metamorphic *P*–*T* paths recorded by rocks can reveal the complete evolution of an orogenic belt, including the superposition of multiple tectonic events, and effectively constrain its tectonic and thermal evolution^18,55^. Furthermore, the chronometer minerals within these rocks, such as zircon and monazite, which have relatively fixed closure temperatures (e.g., for the U–Pb system) or growth temperatures, can be used to pinpoint the initiation and termination of orogeny by dating the ages at which the orogenic belt reached those specific temperatures during its heating and cooling history^56,57^. Consequently, the time interval between these two ages (i.e., the collisional orogenic duration) can reflect the thermal state of the orogen (or lithosphere). Variations in orogenic duration during different supercontinent cycles can reveal the Earth’s long-term thermal and tectonic regime evolution.

**Fig. 3:**
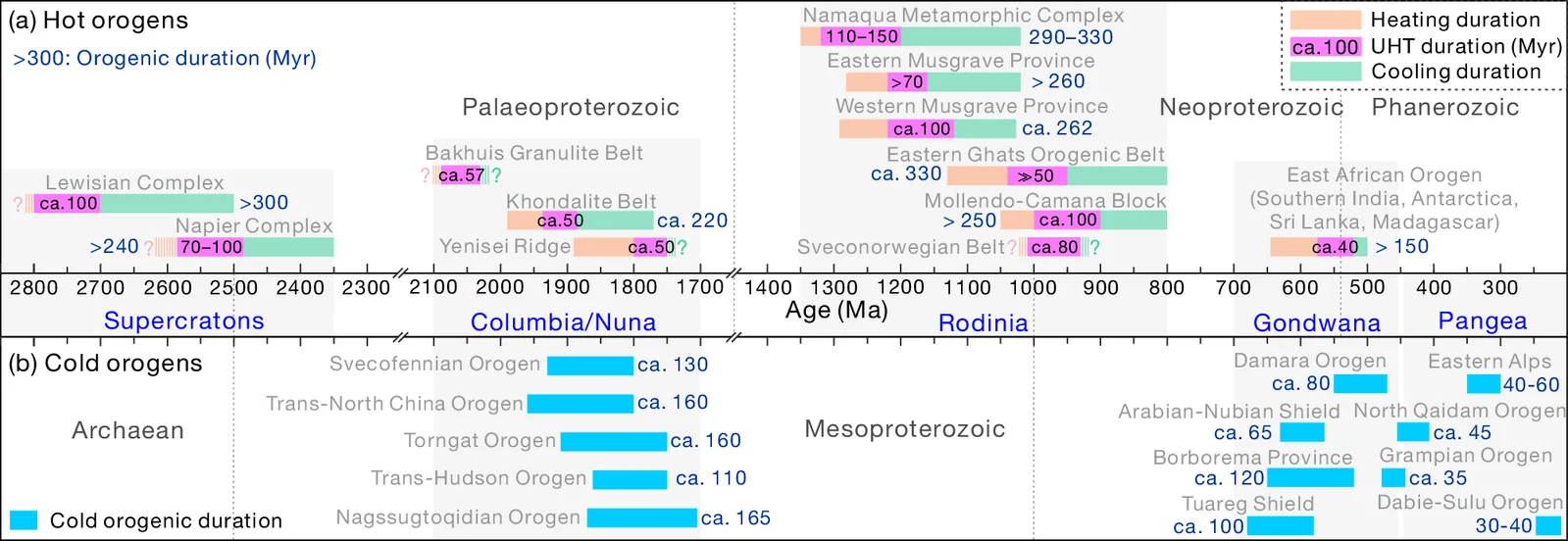
Thermal and temporal evolution for hot and cold orogens from Neoarchaean Supercratons to Phanerozoic Pangea assembly. **a** Thermal evolution from heating to cooling and orogenic duration of representative hot orogens from the Neoarchaean to the Neoproterozoic, showing that the thermal peak and orogenic duration shortened significantly from the Neoarchaean to the Palaeoproterozoic Columbia assembly, became anomalously prolonged during the Mesoproterozoic Rodinia assembly, and then abruptly shortened again during the Neoproterozoic Gondwana assembly. **b** Representative cold orogens developing during the Palaeoproterozoic Columbia assembly, the Neoproterozoic Gondwana assembly, and the Phanerozoic Pangea assembly, exhibiting significantly shorter orogenic duration. The detailed data and references are in Supplementary Data 5.

To study the secular evolution of orogenic duration, data for representative orogenic cycles from each period must be selected based on the same criteria: consistent geochronological methods, similar sample types, and unified standards—particularly important for the different types (e.g., hot [elevated thermal structures^21,22^ and cold [the appearance of low d*T*/d*P*^18^ metamorphic mafic rocks defined in this study]) of orogens. Accordingly, we have chosen representative hot orogens from four key Precambrian supercontinent/supercraton cycles, including the Kenor supercratons, Columbia assembly, Rodinia assembly, and the Gondwana megacontinent (Fig. 1). Using compiled zircon and monazite U–Pb ages and garnet Lu–Hf data, we statistically analyze collisional orogenic cycles and their evolutionary trends (Fig. 3 and Supplementary Data 5).

In the Neoarchaean Napier Complex, UHT metamorphism developed over an extensive area and persisted for 70–100 Myr (ca. 2.59–2.49 Ga; ref. ^58,59^), followed by slow cooling exhumation exceeding 150 Myr, collectively archiving a collisional duration lasting over 240 Myr^21^ (Fig. 3a). Similarly, the Lewisian Complex recorded the UHT and orogenic durations of ca. 100 Myr and over 300 Myr, respectively^60,61^. From the Neoarchaean to the Palaeoproterozoic, both the UHT duration (ca. 50 Myr) and total timescales (ca. 220 Myr) of hot orogens apparently shortened (Fig. 3a). Subsequently, during the Mesoproterozoic-Neoproterozoic, six representative hot orogens exhibit an anomalously extended orogenic duration of ca. 260–330 Myr (Fig. 3a). The recorded UHT metamorphism endured well over 50 Myr, extending to 100 Myr, with post-peak cooling exhumation also spanning over 100–150 Myr (Fig. 3a). The Neoproterozoic-Cambrian East African Orogen, including Southern India, Antarctica, Sri Lanka, and Madagascar, again documents a significantly shortened collisional cycle of over 150 Myr, including ca. 40–60 Myr heating, ca. 40–50 Myr UHT duration, and over 50 Myr cooling period (Fig. 3a; ref. ^62–64^).

Furthermore, limited occurrences of cold orogens associated with the Columbia assembly exhibit markedly shorter orogenic durations than their hot counterparts—predominantly clustering ca. 160 Myr, with some as brief as ca. 110 Myr (Fig. 3b). In contrast, cold orogens became more widespread during the Gondwana megacontinent, with durations further shortened to ca. 65–120 Myr (Fig. 3b). For the Phanerozoic, the orogenic duration sharply decreased to ca. 30–60 Myr (Fig. 3b).

**Fig. 4:**
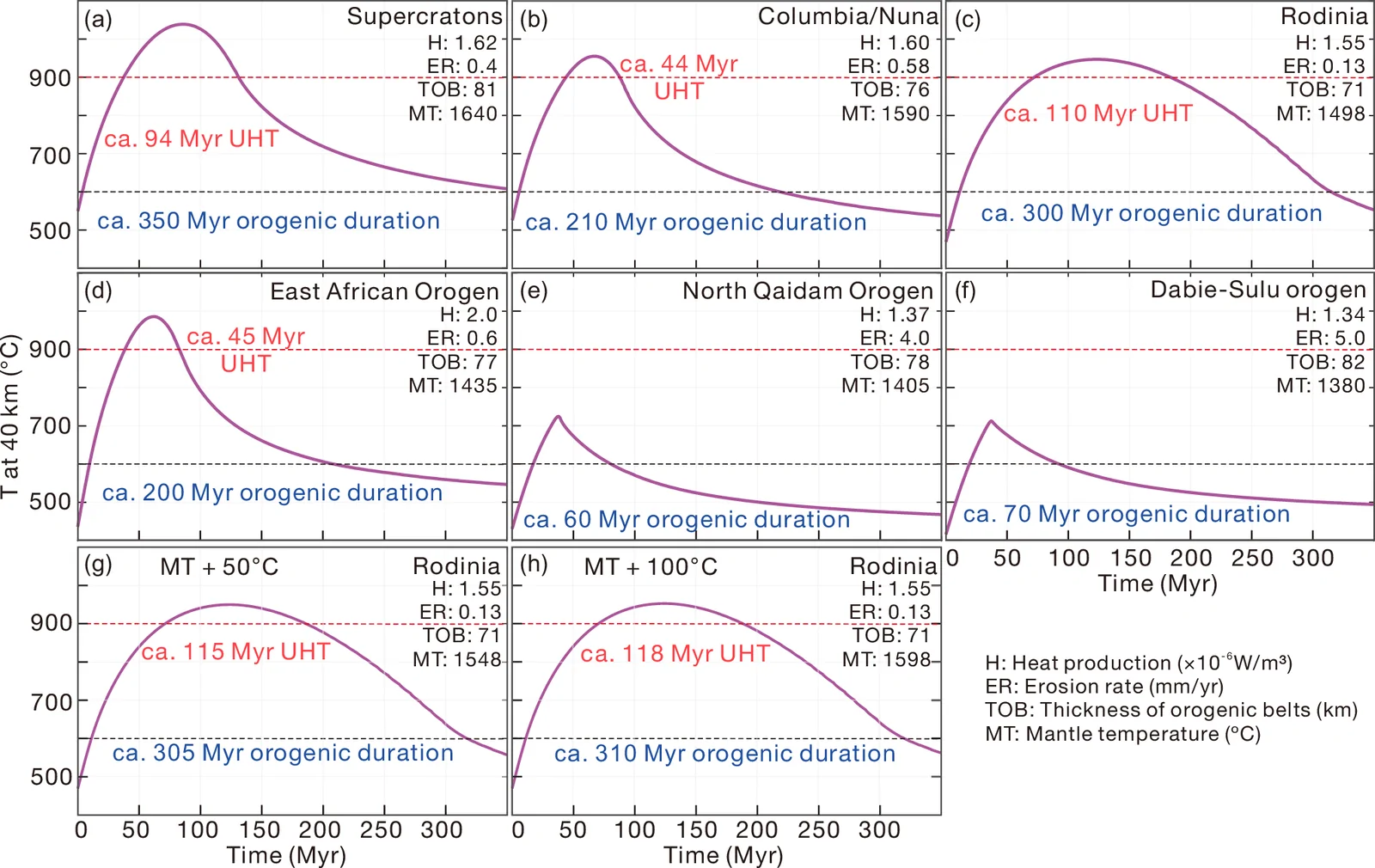
The thermal and temporal evolution of Precambrian hot orogens and Phanerozoic cold orogens, calculated by the one-dimensional thermal simulation (the parameters are presented in Supplementary Data 6). **a–d** Modelled thermal and temporal evolution of hot orogens from the Neoarchaean to the Neoproterozoic, showing results consistent with geological records. **e–f** Modelled thermal and temporal evolution of representative Phanerozoic cold orogens—the North Qaidam Orogen and the Dabie-Sulu Orogen—displaying similarly cold and rapid characteristics as recorded in the geological record. **g–h** Influence of mantle temperature warming during the Mesoproterozoic Rodinia assembly on the thermal and temporal evolution of orogens. From Columbia/Nuna (**b**) to Rodinia (**c**) assembly, both radiogenic heat productivity and mantle temperature decrease, yet the modelled orogenic duration (increasing from ca. 210 Myr to ca. 300 Myr) and thermal state (UHT duration increasing from ca. 44 Myr to ca. 110 Myr) increase significantly. This indicates that the slow erosional rate during the Rodinia period was the dominant controlling factor. In contrast, with the same radiogenic heat productivity, erosional rate, and orogenic thickness as the Rodinia period, an increase in mantle temperature (MT) by 50 °C (**g**) and 100 °C (**h**) only prolongs the orogenic and UHT durations by 5 Myr and 8–10 Myr, respectively, indicating a relatively minor effect.

Heat distribution of an orogen is governed by conduction, advection, and radiogenic production^65^. The contributions of these three heat components are primarily controlled by factors such as mantle potential temperature, lithospheric and crustal thickness, erosion rate, and radiogenic heat production^7,8,65^. Extensive geological evidence has revealed that these factors followed distinct evolutionary trends throughout Earth’s history (Supplementary Fig. 9). Hence, one-dimensional thermal simulations (the specific methods of simulation are described in the “Methods”) incorporating these controlling factors as key parameters (we employed the model of Tang et al.^66^ to calculate crustal thickness, although some researchers^67^ also posed several limitations on this model, since the Eu anomaly is controlled by multiple complex factors) were conducted, with the aim of probing the thermal and temporal evolution of orogenic belts. Our modelling results successfully reproduce the thermal states and temporal durations of collisional orogenesis from the geological records during the early Precambrian (hot orogens) to the Phanerozoic (Fig. 4). The simulation results indicate that the duration of collisional orogenesis (over 600 °C) decreased from ca. 350 Myr (Neoarchaean) to ca. 210 Myr (Palaeoproterozoic), then increased to ca. 300 Myr (Mesoproterozoic), and finally shortened again to ca. 200 Myr (Neoproterozoic) (Fig. 4a–d). The duration of UHT conditions exhibited a consistent variation, correspondingly decreasing from ca. 94 Myr to ca. 44 Myr, then rising to ca. 110 Myr, before declining again to ca. 45 Myr (Fig. 4a–d).

**Fig. 5:**
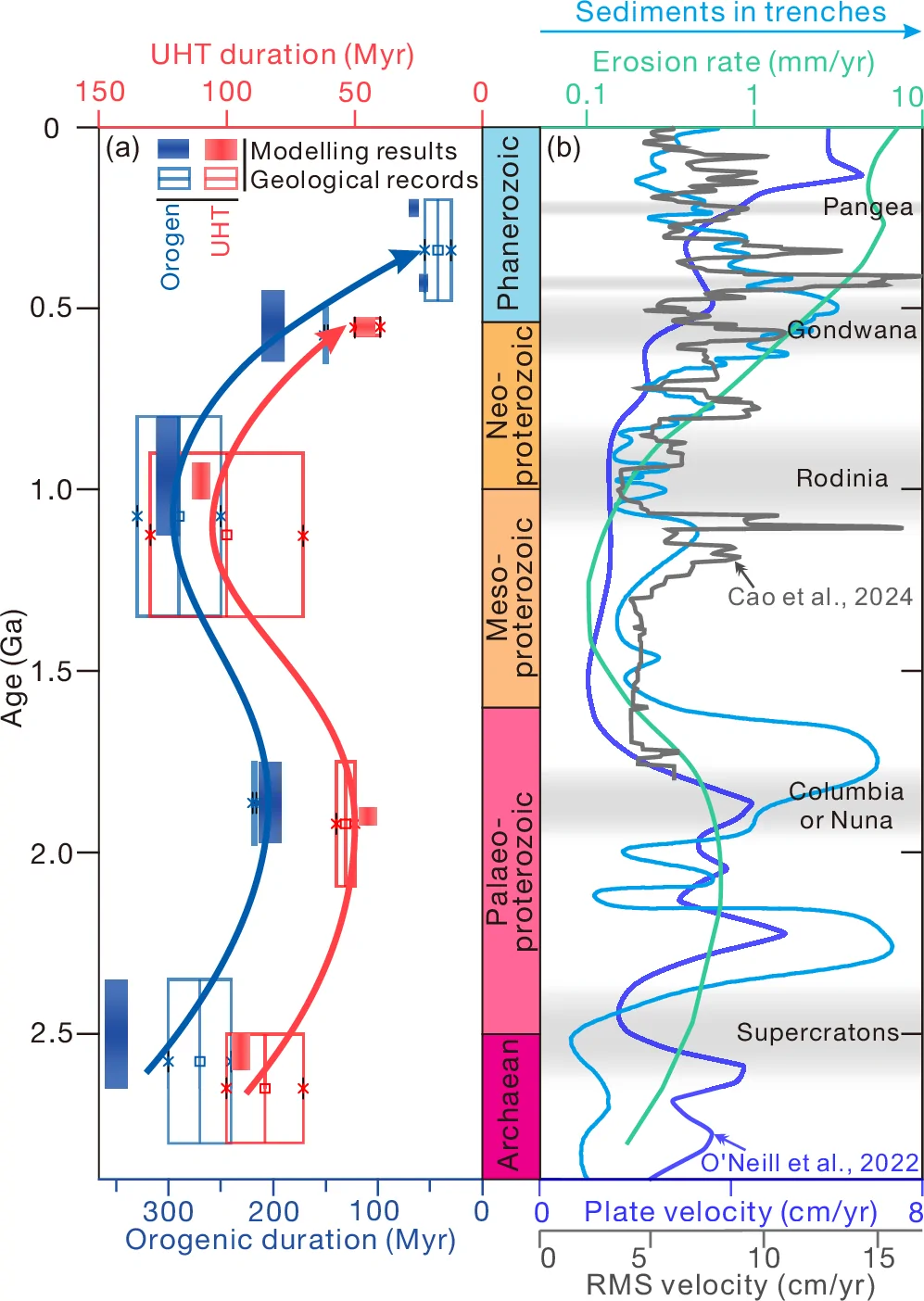
Secular evolution of orogenic thermal, temporal, and geodynamic parameters during supercontinent cycles, Neoarchaean to Phanerozoic. **a** The non-monotonic evolution of the duration of collisional orogenic activity and UHT metamorphism for representative orogens from each period, based on the geological records (the raw data are in Supplementary Data 5) and thermal simulation results. **b** Secular trends of the erosion rate^17^, plate velocities^72,78^, and sediments in trenches^83^. Plate velocities after 1.8 Ga primarily cite results from the full-plate reconstruction models^78^, whereas plate-velocity curves before 1.8 Ga are from the reconstructed models based on passive margin record^72^, due to the lack of full-plate reconstruction data. RMS-root mean square.

Geodynamic modelling and geological records indicate that orogenic belts in the early Precambrian (Archaean-Proterozoic) exhibited hotter thermal structures and longer durations compared to modern systems^7–9,16^. However, the temporal evolution pattern of these hot orogens during the early Precambrian remains unclear. Numerical models, based on a secular decline in mantle potential temperature since the Neoarchaean^68^ (Supplementary Fig. 9a), predict a progressive cooling and acceleration of orogenic thermomechanical processes throughout this period^7,9^. In contrast, both compiled geological data and thermal modelling results reveal a non‑monotonic evolutionary trend (Fig. 5a). Specifically, duration of hot orogenic activity and UHT metamorphism initially shortened from the Neoarchaean to the Palaeoproterozoic assembly of Columbia, then lengthened during Mesoproterozoic Rodinia assembly, before undergoing rapid shortening (Fig. 5a).

### Alternating tectonic regimes during the Archaean-Proterozoic

Collisional orogens are primary products of Earth’s tectonic activity, and the evolution of their duration and thermal state reflects the plate properties and dynamic processes of the lithosphere^14,15^. The non-monotonic cyclical evolution of orogenic belts revealed in this study may reflect alternating tectonic regimes during the Archaean-Proterozoic. This alternating thermal evolution is likely a response to changing lithospheric coupling parameters such as the plateness and mobility (e.g., Fig. 5), which are an overall expression of the tectonic regimes under which the Earth has operated through its history^1^. Plateness refers to the degree of rigidity of tectonic plates^1,2^. Plate tectonics is characterised by high degrees of plateness (i.e., rigid plates with deformation focused at plate boundaries). In contrast, non-plate tectonic mobile lid regimes (e.g., sluggish lid) may involve broader zones of plate deformation, and hence lower plateness. Sluggish lid regimes will be facilitated by magmatism and UHT metamorphism, which will decrease, at least locally, plate rigidity^1,4^. This will also likely lead to non-uniform rates of plate motion. Thus, the non-monotonic evolution of orogenic belts can be viewed in response to changing plateness and mobility, leading to an alternation between sluggish and active plate-tectonic states. The Neoarchaean is characterised by likely sluggish lid setting progressing to an active plate tectonic phase in the Palaeoproterozoic, then a return to sluggishness in the Mesoproterozoic, which corresponds with Earth’s Middle Age^69^, and finally a transition to contemporary active plate tectonics from the Neoproterozoic into the Phanerozoic.

During the Neoarchaean, under high mantle temperatures^68^, the hot and weak lithosphere during orogenesis favored broadly distributed tectonic deformation zones^7,21,70^, accompanied by prolonged orogenic cycles and a high-thermal state (ca. 100 Myr UHT duration) (Fig. 5a). It is noteworthy that the earliest appearance of HP to eclogite-facies metapelites (ref. ^61,71^) under low d*T*/d*P* ratios during this period may record the transition from some pre-plate tectonic regime to the plate tectonics^1,3,10,61^. However, widespread high to moderate d*T*/d*P* metamorphism^55^, combined with extensive broad deformation domains and prolonged high-thermal states, implies relatively low plateness and surface mobility^2^ consistent with inferred plate-velocity proxies^72^ (Fig. 5b), revealing a relatively sluggish tectonic regime^10^.

As mantle temperatures gradually cooled (Supplementary Fig. 9a), the durations of orogeny and UHT conditions are substantially shortened during the Palaeoproterozoic (Fig. 5a). Collisional orogeny at this time was distinguished by localized compressional deformation along major shear zones^7–9^, similar geometries to Phanerozoic orogens, manifesting as the development of numerous linear orogenic belts within short time intervals^36^, which implies significantly increased lithospheric plateness^2^. Although metamorphism was still dominated by high to moderate d*T*/d*P* types^55^, low d*T*/d*P* metamorphic products, such as eclogite^73–75^, began to appear more frequently in localized areas. These limited occurrences of low d*T*/d*P* rocks are all distributed within orogenic belts along supercontinental margins and formed in subduction environments^76^, suggesting that cold subduction similar to the present day was widespread during the Palaeoproterozoic^73–75^. Eclogitization of mafic rocks (e.g., oceanic crust) distinctly increases rock density, generating negative buoyancy that enhances slab pull and facilitates sustained subduction^77^. This process strengthens coupling between the lithospheric lid and mantle convection, thereby markedly increasing surface mobility of the lithosphere^2^, which is consistent with the concurrent acceleration in plate velocities^72^ (Fig. 5b). Furthermore, the emergence of the short-duration cold orogens (Fig. 3b) further supports the inference that the lithospheric plateness and mobility were significantly enhanced during Columbia assembly. Collectively, these lithospheric signatures signify the initial establishment, at least locally, of a modern plate tectonic regime^1,10^.

Despite continued mantle cooling, the Mesoproterozoic collisional orogeny exhibited prolonged duration and elevated thermal states (Fig. 5a). This period lacks low d*T*/d*P* metamorphic records^55^, retains UHT durations similar to the Neoarchaean, and shows persistently slow plate motion^72,78^ (Fig. 5b), indicating significantly reduced plate mobility. Although mantle warming (ca. 50–100 °C) has been invoked to explain these hot orogens^17,66,79,80^, it contradicts secular mantle cooling trends recorded by the mantle-derived rocks^68^. Our one-dimensional thermal modelling shows that remarkably low erosion rates^17^ (ca. 0.13 mm/yr) led to ca. 270 Myr orogenic exhumation (Supplementary Fig. 10f), enabling protracted crustal heating^65^ and anomalous durations and thermal states (Fig. 4c). In contrast, the effect of mantle warming (50–100 °C) is very limited (UHT and orogenic durations increased by ca. 5–10 Myr; Fig. 4g, h). The remarkably low erosion rates may be linked to the distinctive breakup mode of the Columbia supercontinent, which limited passive continental margins^81^ and maintained largely major continental stability during Rodinia assembly^69,78,80^. This resulted in prolonged continental convergence, continuously thin active continental crust^66^, slow exhumation-cooling rates^17^, and sediment starvation^81–83^ (Fig. 5b). This lithospheric coupling directly contributed to the remarkably long and hot collisional orogenic processes, revealing that the accelerating plate tectonic regime that developed during Columbia assembly reverted to a relatively sluggish state during Rodinia assembly.

Into the Neoproterozoic-Phanerozoic, orogenic records resumed a trend of mantle cooling and plate acceleration (Fig. 5a). This corresponds to the widespread occurrence of cool subduction records—such as blueschist and HP–UHP metamorphism^55^—along narrow tectonic deformation zones since the Neoproterozoic^76^, implying a rapid increase in lithospheric plateness and mobility^2^. Seismic profile data reveal a widespread absence of mafic lower crust in Phanerozoic orogenic belts compared to the Archaean-Proterozoic^84^. This indicates that extensive eclogitization of mafic lower crust during Phanerozoic continental collision facilitated its gravitational foundering into the deep mantle and drove extensive uplift and erosion of the continental lithosphere. This process drove Phanerozoic deep continental subduction (e.g., the Dabie-Sulu Orogen^85^), thereby significantly accelerating lithospheric plate velocities and shortening collisional orogenic durations (Fig. 5). These observations mark the full establishment of the modern plate tectonic regime^10^, signifying Earth’s global transition from radiogenic heat‑buffered systems toward mechanically efficient tectonic regimes capable of rapid energy redistribution^22^.

In summary, our study constrains the duration of a hot orogen in the Palaeoproterozoic. Compiling data on the temporal and thermal evolution of orogenic belts (i.e., hot versus cold orogens) across supercontinent/supercraton cycles from the Neoarchaean to the Phanerozoic, we delineate a non-monotonic cyclical evolutionary trend. This trend, integrated with lithospheric proxy records such as plate motion and tectonic deformation features, reveals that Earth underwent alternating tectonic regimes^1,12^. During the Neoarchaean-Proterozoic, the lithosphere‑mantle system alternated between a relatively sluggish, non-rigid mobile lid state and an active rigid plate-tectonic behaviour, ultimately evolving into the mature modern plate tectonic regime by the late Neoproterozoic.

## Methods

### Trace element analysis of garnet

Garnet trace element analysis (Supplementary Data 1) was conducted via laser ablation inductively coupled plasma mass spectrometry (LA-ICP-MS) at Wuhan Sample Solution Analytical Technology Co., Ltd. (Wuhan, China). The operating conditions for the laser ablation system, ICP-MS instrument, and data reduction followed ref. ^86^. Laser sampling was performed using a GeolasPro 193 nm ArF excimer laser system coupled to an Agilent 7700e ICP‑MS. Helium served as the carrier gas and argon as the make‑up gas, mixed via a T-connector before introduction into the ICP. A “wire” signal smoothing device was included in this laser ablation system^87^. Analyses employed a 50 µm spot size at a 6 Hz repetition rate. Trace element compositions were calibrated against BHVO-2G, BCR-2G, and BIR-1G without internal standardization^88^. Each analysis comprised ~20–30 s background acquisition followed by 50 s sample ablation. Data processing, including background correction, signal integration, time‑drift correction, and quantitative calibration, was performed using ICPMSDataCal software^88^.

### In situ garnet Lu–Hf analysis

In situ Lu–Hf analysis (Supplementary Data 2) of garnet was performed by LA-ICP-MS at the John de Laeter Center, Curtin University, Australia. Analytical procedures followed ref. ^31^, using a Resolution 193 nm excimer laser coupled to an Agilent 8900 ICP-QQQ. A reaction gas mixture (20% NH_3_, 80% He) was employed. Analyses used a spot size of 130 µm at 10 Hz with a fluence of ~2.5 J·cm^−2^. Garnet reference materials GWA-1 and GWA-2 yielded Lu–Hf ages of 1267 ± 15 Ma (initial ^176^Hf/^177^Hf = 0.2816 ± 0.0032) and 933 ± 39 Ma (initial ^176^Hf/^177^Hf = 0.2808 ± 0.0025), in agreement with published isotope‑dilution values^89^. Data reduction and age calculation via inverse isochron regression were performed using Iolite4^90^ and IsolotR^91^ to minimize error correlation between ^176^Lu/^177^Hf and ^176^Hf/^177^Hf.

### Monazite U–Pb dating

Geochronologic analysis of monazite comprised both separated-grain (Supplementary Data 3) and texturally controlled (Supplementary Data 4) in situ U–Pb dating. Separated grains were embedded in epoxy and polished to expose their midsections, whereas texturally controlled grains in thin section were identified via TIMA scanning. Chemical zoning in both monazite types was characterised by backscattered electron imaging and X-ray mapping using a CAMECA SX51 electron microprobe at the State Key Laboratory of Lithospheric and Environmental Coevolution, Institute of Geology and Geophysics, Chinese Academy of Sciences, Beijing, China. U–Pb dating was performed by LA-ICP-MS at Wuhan SampleSolution Analytical Technology Co., Ltd. (Wuhan, China). Analyses used a GeolasPro 193 nm ArF excimer laser system coupled to an Agilent 7700e ICP-MS. Helium served as the carrier gas and argon as the make-up gas, mixed via a T-connector before introduction into the ICP; a signal-smoothing device was employed to ensure stable signals at low repetition rates^87^, which is advantageous for dating high-U minerals^92^. Laser spot sizes and repetition rates were set to 24 μm (separated grains) or 16 μm (texturally controlled grains) and 2 Hz, respectively. The monazite standard 44069 and the glass NIST610 were used as external standards for U–Pb dating and trace-element calibration, respectively. Each analysis included a 20–30 s background measurement followed by 50 s of sample ablation. Data reduction, including background correction, time-drift correction, and quantitative calibration, was carried out with the Excel-based software ICPMSDataCal^88,93^. Concordia diagrams and weighted-mean ages were calculated using IsoplotR^91^.

### 1D thermal modelling

A simplified one-dimensional thermal model, applied to vertical columns through the lithosphere, was used to reconstruct the geothermal gradient during the processes of collisional thickening and erosion. This model incorporates both non-linear heat diffusion and radiogenic heat production. The governing heat flow equation is expressed as:

1 $$\rho \frac{\partial \left[{Cp}\left(T\right)T\right]}{\partial t}=\frac{\partial }{\partial z}\left[k\left(T\right)\frac{\partial T}{\partial z}\right]-\rho v(z,t)\frac{\partial \left[{Cp}\left(T\right)T\right]}{\partial z}+A\left(z,t\right)$$

Where *T* (K) is temperature, *t* (s) is time, *ρ* (kg/m^3^) is density, *Cp* (J/kg/K) is heat capacity, *k* (W/m/K) is thermal conductivity, *z* (m) is depth, *v* (mm/year) is the vertical velocity (incorporating both thickening and erosion effects), and *A* (W/m^3^) represents radiogenic heat production. This equation was numerically solved using an explicit finite difference method in MATLAB, and the source code refers to ref. ^94^. The boundary conditions for the model were set with a constant surface temperature of 0 °C and a fixed temperature at the base of the lithosphere. Parameters such as the lithospheric thickness, crustal thickness (the thickness of active continental crust^66^ plus the thickness of average continental crust of 30 km), temperature at the base, radiogenic heat production in the crust, erosion rate, erosion delay time, and the initial geothermal gradient are listed in Supplementary Data 6 for each orogen. The crust and mantle densities were set to 2800 and 3300 kg/m³, respectively. The model assumes that the crust experiences an instantaneous doubling of thickness through homogeneous deformation during collision. Temperature-dependent thermal conductivity and heat capacity values for the crust were derived from ref. ^95^, while the parameters for the lithospheric mantle were taken from ref. ^96^.

The 1D thermal modelling is simplified to capture the primary thermal evolution of a vertically thickened lithospheric column during collision and erosion. Assumptions including instantaneous crustal thickening and a fixed basal temperature are idealizations and do not represent the full thermos-mechanical complexity of collisional orogenesis. Compared to 1D modelling, the 2D thermos-mechanical models (e.g., ref. ^7^) can explicitly resolve lateral heat transfer, evolving collision geometry, spontaneous convergence, slab breakoff, and exhumation processes. However, our approach is not intended to reconstruct the full dynamics of orogens, but to provide a consistent comparative framework for evaluating how secular variations in lithospheric structure, basal thermal conditions, radiogenic heat production, and erosion rate affect the duration of elevated thermal conditions. The model results are therefore interpreted as primary thermal scaling relationships rather than full geodynamic reconstructions.

## Supplementary information

Supplementary Information

Description of Additional Supplementary Files

Peer Review File

## Source data

Source Data

## Data Availability

All data generated in this study are provided in the Supplementary Source Data file. Source data are provided with this paper.

## Code availability

The source code of the 1D thermal modelling during the study is available at https://github.com/LIU-Jiahui/Thermal_Modeling_Liuetal2024 and https://doi.org/10.5281/zenodo.13987165.
