# Supplementary material for: Secular change in orogenic duration reveals alternating tectonic regimes during the Archaean-Proterozoic
Source: Nat Commun. 2026 Aug 11;17:9621. doi: 10.1038/s41467-026-76530-3 (PMC13554081; doi:10.1038/s41467-026-76530-3)
Supplement: Supplementary file 1 [file 41467_2026_76530_MOESM1_ESM.pdf]

Secular change in orogenic duration reveals alternating tectonic regimes during the Archaean-Proterozoic

Lei Zou, Guangyu Huang*, Jinghui Guo, Peter A. Cawood, Jia-Hui Liu, Pinghua Liu, Shujuan Jiao, Xiao-Ping Xia, Huijuan Li, Jun-Bo Zhang, Meiyun Huang, Lifei Zhang, Guochun Zhao*

*Corresponding authors: huangguangyu@mail.iggcas.ac.cn; gzhao@hku.hk

Supplementary Information

1. Sample description and garnet genesis

UHT granulite in the western Khondalite Belt is garnet-rich, and records peak conditions of 890–940°C at 7.5–9.8 kbar as evidenced by the characteristic garnet + corundum + spinel assemblage[1]. Garnet in the granulite occurs in two morphotypes: (1) 4–8 mm porphyroblasts displaying core-mantle-rim zoning and (2) irregular fine-grained crystals (Supplementary Fig. 2a–b).

The porphyroblastic cores preserve an early prograde muscovite-plagioclase-quartz assemblage (Supplementary Fig. 2c–e). Their bell-shaped Y+HREE zoning (Supplementary Fig. 3) and outwardly decreasing Yb/Dy and Yb/Er ratios (Supplementary Fig. 4a–b) are diagnostic of Rayleigh fractionation during equilibrium garnet growth[2,3]. Slightly increasing Sc and Cr contents (Supplementary Fig. 4c–d) suggest garnet core growth was concurrent with or slightly later than biotite due to the preferential partitioning of Sc and Cr into biotite[4]. Critically, the slightly outward-increasing Ti, Zr, and P concentrations (Supplementary Fig. 4e–f) signify growth during initial heating under subsolidus conditions, where partial melting was negligible, and accessory minerals remained stable[5,6]. These lines of evidence robustly constrain the garnet cores to the early prograde subsolidus stage (ca. 7–8 kbar/600–700 °C; ref.[1]).

A distinct shift in metamorphic regime is recorded by the garnet mantle. The sharp increase in Sc and Cr (Supplementary Fig. 4c–d) unambiguously signals the breakdown of biotite via dehydration melting reactions[7], consistent with a temperature rise from ca. 830 °C to ca. 950 °C inferred from the concomitant Ti increase (Supplementary Fig. 4e). The significant enrichment of Zr and P in the mantle (Supplementary Figs. 3g and 4f) directly links to the dissolution of zircon and monazite during anatexis[8]. Therefore, the garnet mantle unequivocally formed through peritectic reactions during the heating stage approaching UHT conditions.

The porphyroblastic rims and fine-grained garnet are characterised by retrograde sillimanite + biotite inclusions, decreased Ti zoning, and inverted Ca/Ti ratios (Supplementary Fig. 4i–l), indicating their growth during post-peak retrograde cooling[9] from ca. 950 °C to ca. 800 °C (Supplementary Fig. 4i).

2. Supplementary Figures

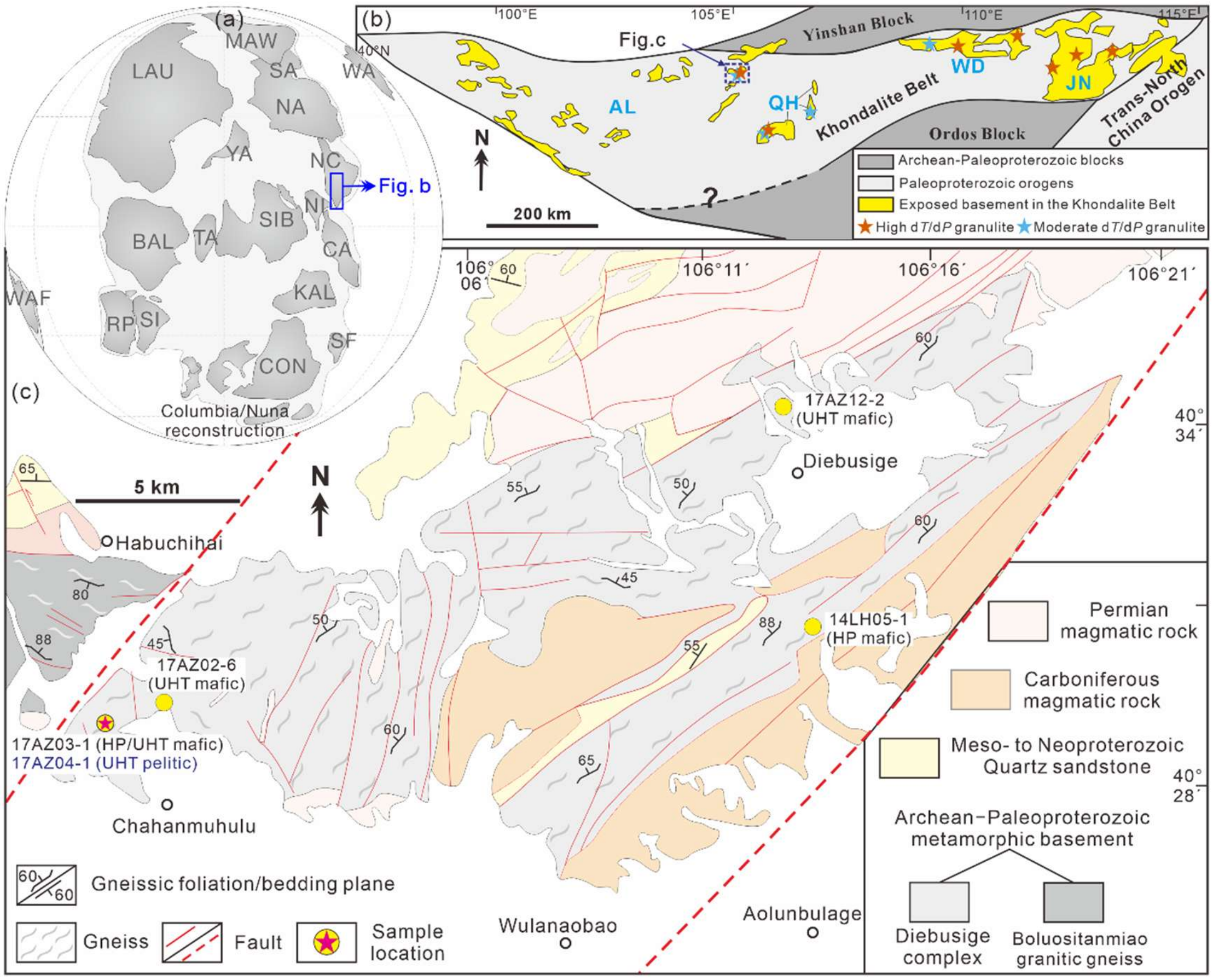

Supplementary Figure 1. Geological sketch map of the metamorphic basement of the Khondalite Belt, supercontinent reconstruction, and sample collection locations. (a) Reconstruction of Columbia/Nuna supercontinent (this map is adapted and colorized from ref.[10] with permission from Elsevier), including the position of North China Craton (NC). LAU-Laurentia, AMA-Amazonia, SF-São Francisco, BAL-Baltica, RP-Rayner Province, MAW-Mawson, SA-South Australia, NA-North Australia, WA-West Australia, YA-Yangtze, CA-Cathaysia, NC-North China, SIB-Siberia, TA-Tarim, SI-South India, NI-North India, CON-Congo, KAL-Kalahari, WAF-West Africa. (b) Exposed basement and high to moderate dT/dP granulites in Khondalite Belt (after ref.[11] and ref.[12]). AL: Alxa terrane; QH: Qianlishan-Helanshan terrane; WD: Wulashan-Daqingshan terrane; JN: Jining-Liangcheng terrane. (c) Simplified geological map of the Diebusige area in the eastern Alxa terrane. Both maps (b and c) are adapted and colorized from ref.[13] with permission from John Wiley and Sons.

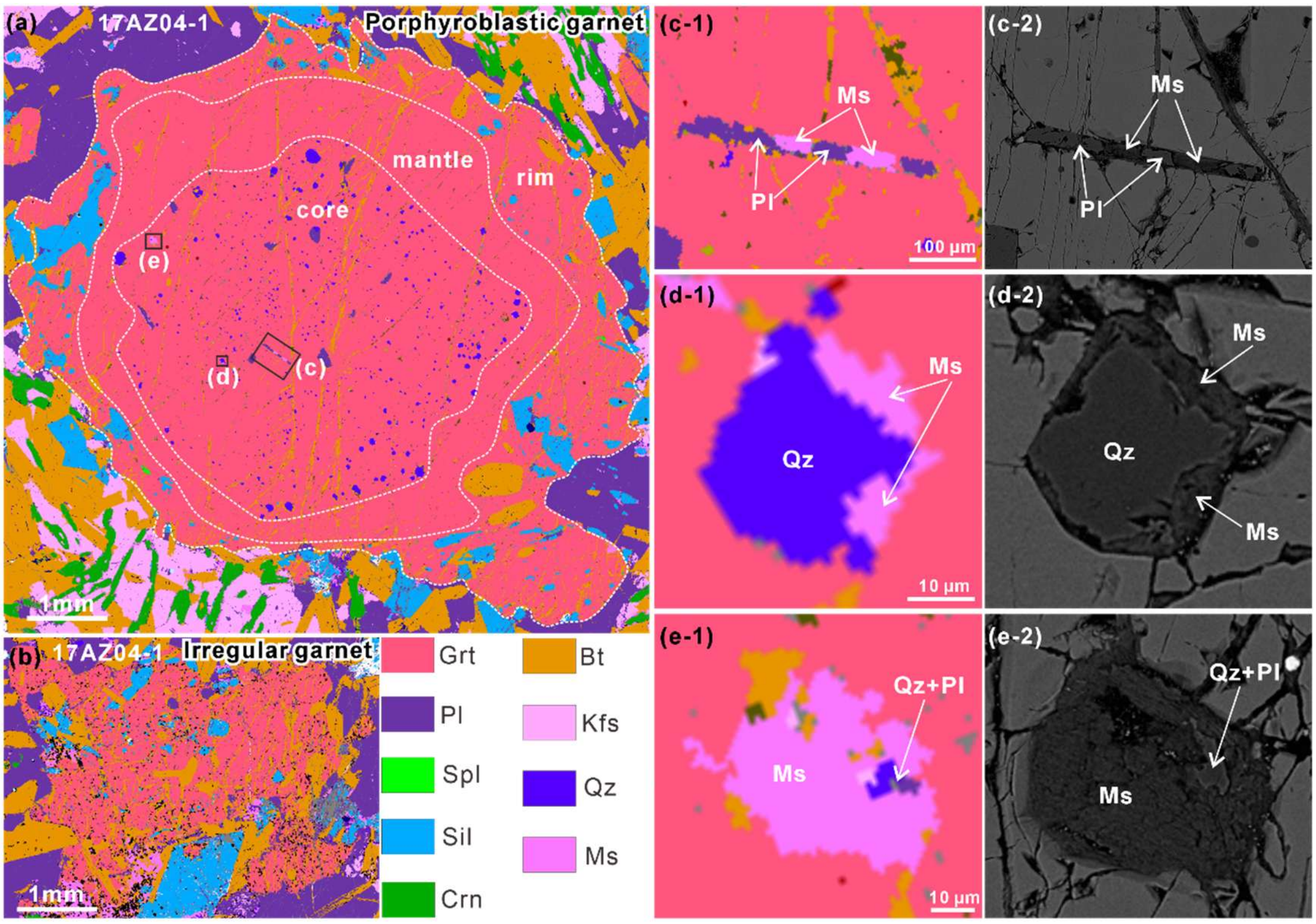

Supplementary Figure 2. TIMA mapping images of garnet and its internal inclusion characteristics. (a) The morphological characteristics, textural features, inclusion assemblages, and delineation of microdomains in coarse-grained porphyroblastic garnet from the pelitic UHT granulite sample 17AZ04-1. (b) The morphological and inclusion-assemblages characteristics of fine-grained irregular garnet. (c–e) The prograde metamorphic mineral assemblage of muscovite + plagioclase + quartz in the core of the porphyroblastic garnet. Grt, garnet; Sil, sillimanite; Bt, biotite; Pl, plagioclase; Kfs, K-feldspar; Crn, corundum; Spl, spinel; Qz, quartz; Ms, muscovite.

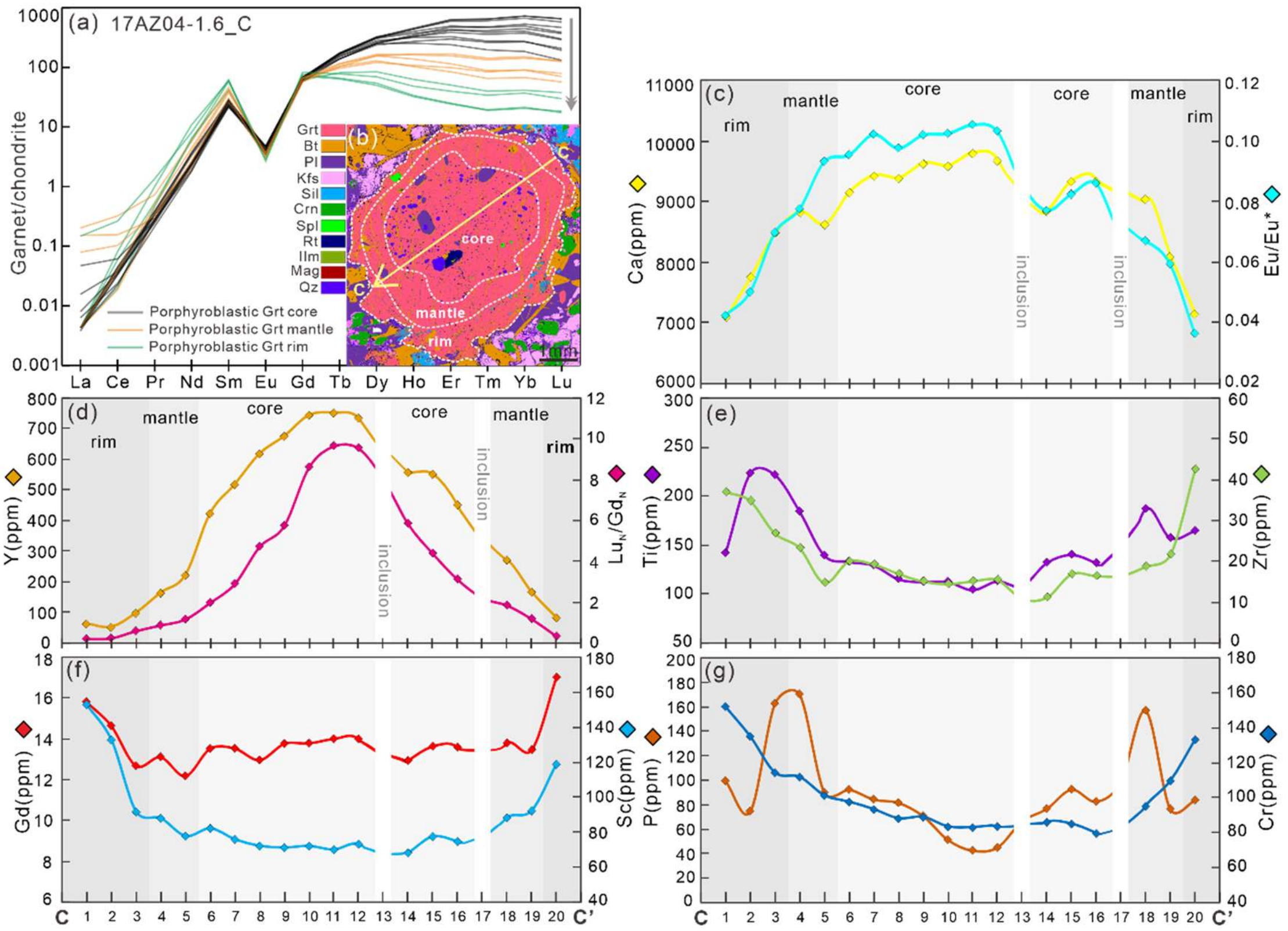

Supplementary Figure 3. REE distribution patterns and Ca and trace element profiles in garnet. (a) Chondrite-normalised REE patterns for porphyroblastic garnet from the pelitic granulite sample 17AZ04-1. The chondrite values are from ref.[14]. (b) TIMA image showing the analysed garnet in sample 17AZ04-1.6 with core-mantle-rim texture. The yellow arrow shows the path of the analytical traverse. (c–g) Ca and trace elemengs zoning along the analysed traverse, showing the typical bell-shaped distribution for the Y and Lu_N/Gd_N and the bowl-shaped distribution for the Ti and Zr. Grt, garnet; Sil, sillimanite; Rt, rutile; Bt, biotite; Pl, plagioclase; Kfs, K-feldspar; Crn, corundum; Spl, spinel; Ilm, ilmenite; Mag, magnetite; Qz, quartz.

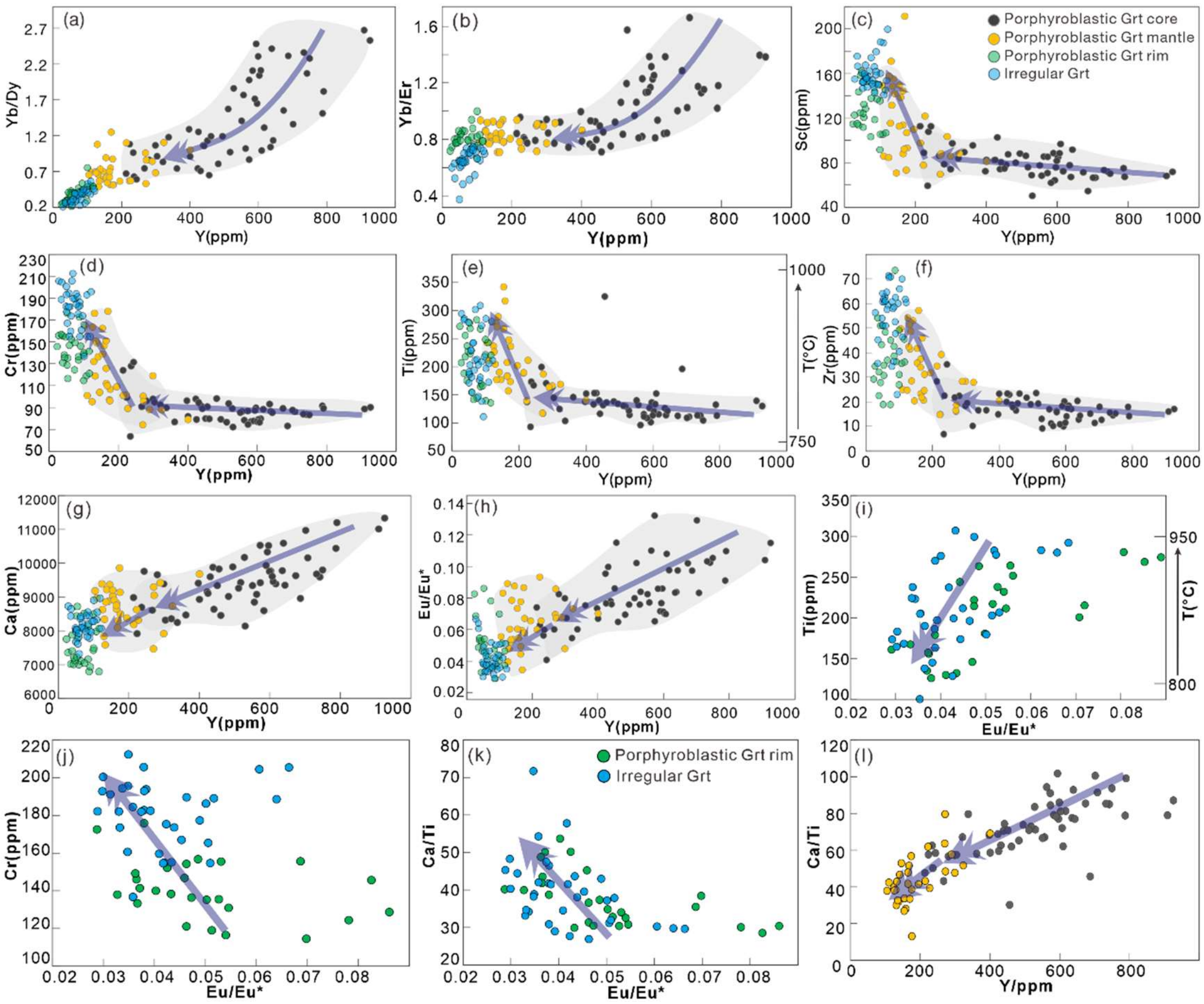

Supplementary Figure 4. The Ca and trace element chemical features in terms of Y and Eu/Eu* for garnet from the pelitic granulite sample 17AZ04-1. The arrows represent changes of trace elements during progressive garnet growth.

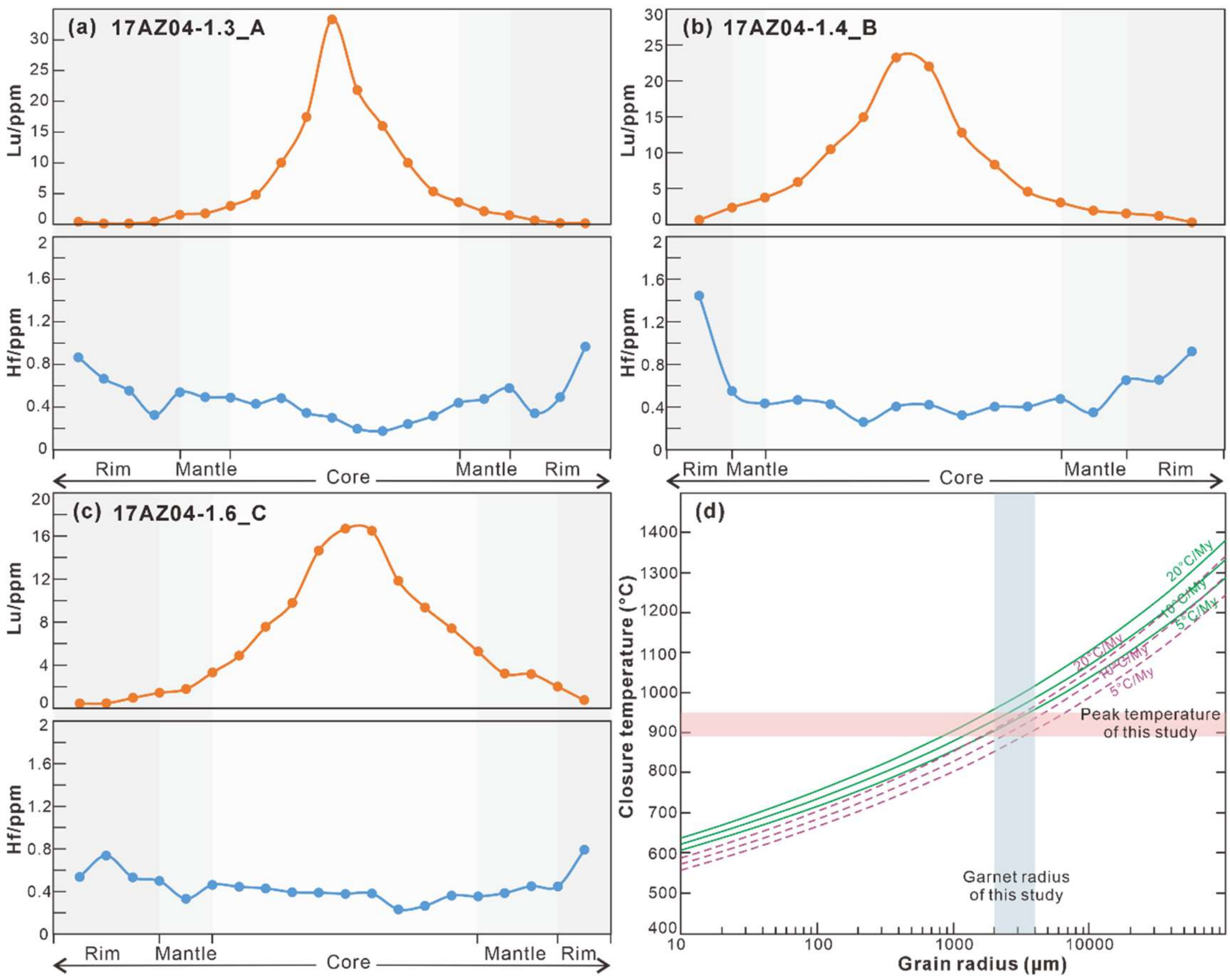

Supplementary Figure 5. Profiles of Lu and Hf elements in garnet and calculated closure temperature of the Lu-Hf system. (a–c) The Lu and Hf compositional zoning of porphyroblastic garnet from the pelitic granulite sample. (d) Theoretical calculation of Lu-Hf closure temperature in garnet based on the method of ref.[15] using the diffusion coefficients of ref.[16] and assuming the cooling rate of 5, 10, and 20 °C/Myr. The green solid lines and the purple dashed lines represent the closure temperature curves of the Hf and Lu elements, respectively.

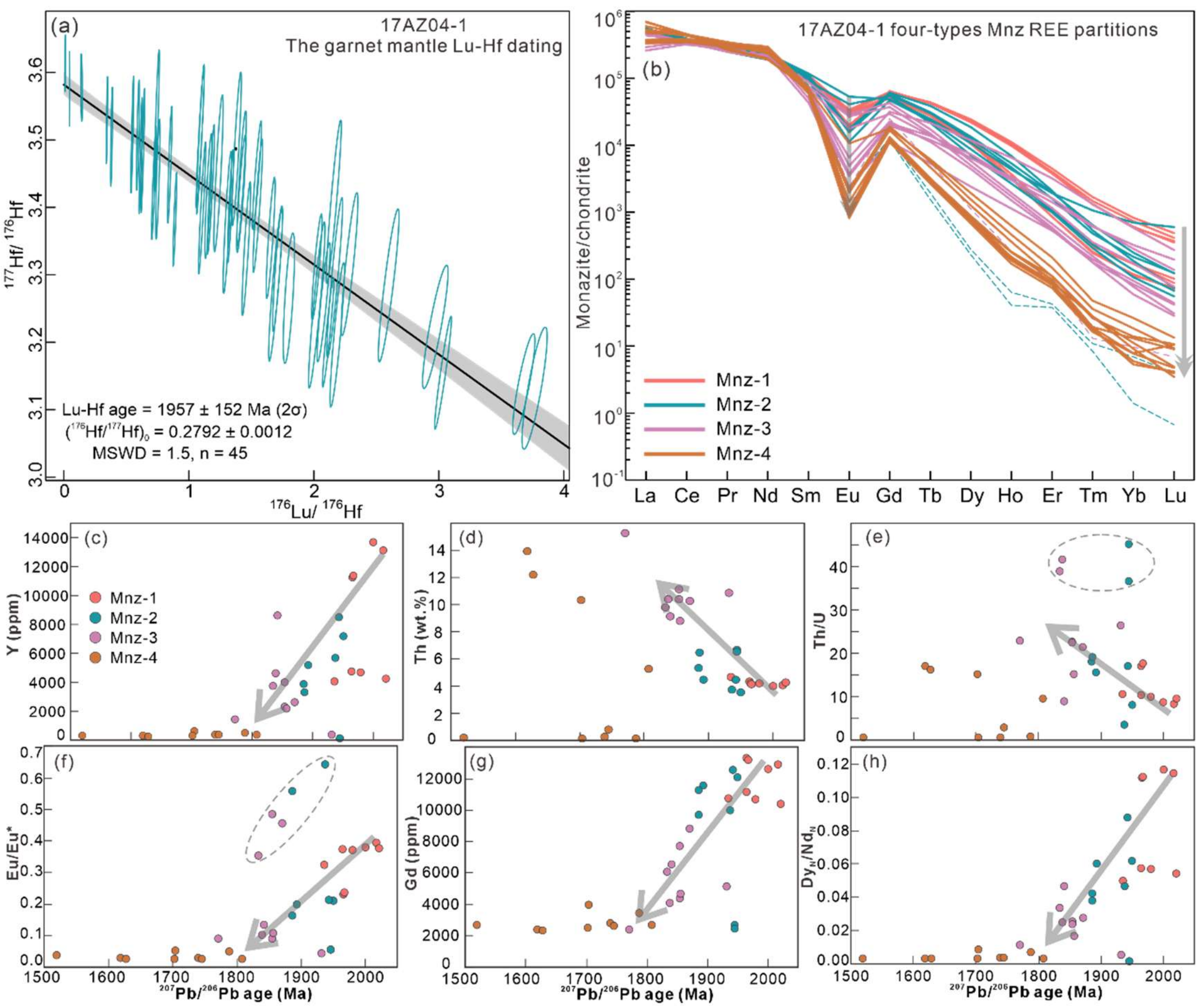

Supplementary Figure 6. Garnet and monazite geochronology and geochemistry results. (a) Lu-Hf isochron plots for porphyroblastic garnet mantle from the pelitic granulite samples; (b) Chondrite-normalised REE patterns of the texturally controlled four-types monazite microdomains; (c–h) Compositional characteristics of the texturally controlled four-types monazite microdomains from the pelitic granulite samples. The arrows represent the variation trend of chemical composition with reducing $^{207}Pb/^{206}Pb$ ages.

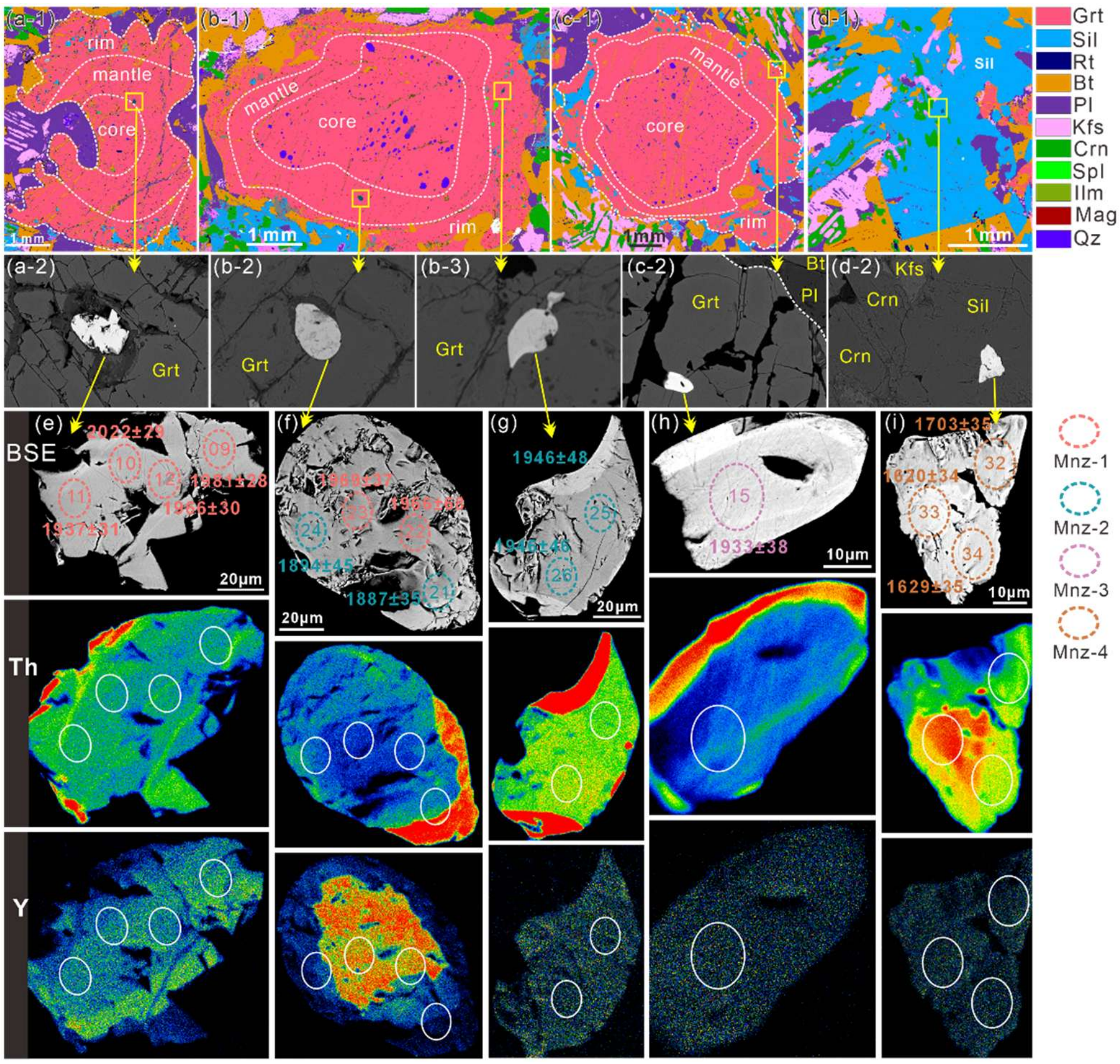

Supplementary Figure 7. TIMA image, BSE image, and X-ray chemical map (Th and Y) showing internal structures of monazite inclusion in porphyroblastic garnet and sillimanite of the pelitic granulite sample 17AZ04-1. The texturally controlled monazites are divided into four types of microdomains (Mnz-1, Mnz-2, Mnz-3, and Mnz-4). Grt, garnet; Sil, sillimanite; Rt, rutile; Bt, biotite; Pl, plagioclase; Kfs, K-feldspar; Crn, corundum; Spl, spinel; Ilm, ilmenite; Mag, magnetite; Qz, quartz.

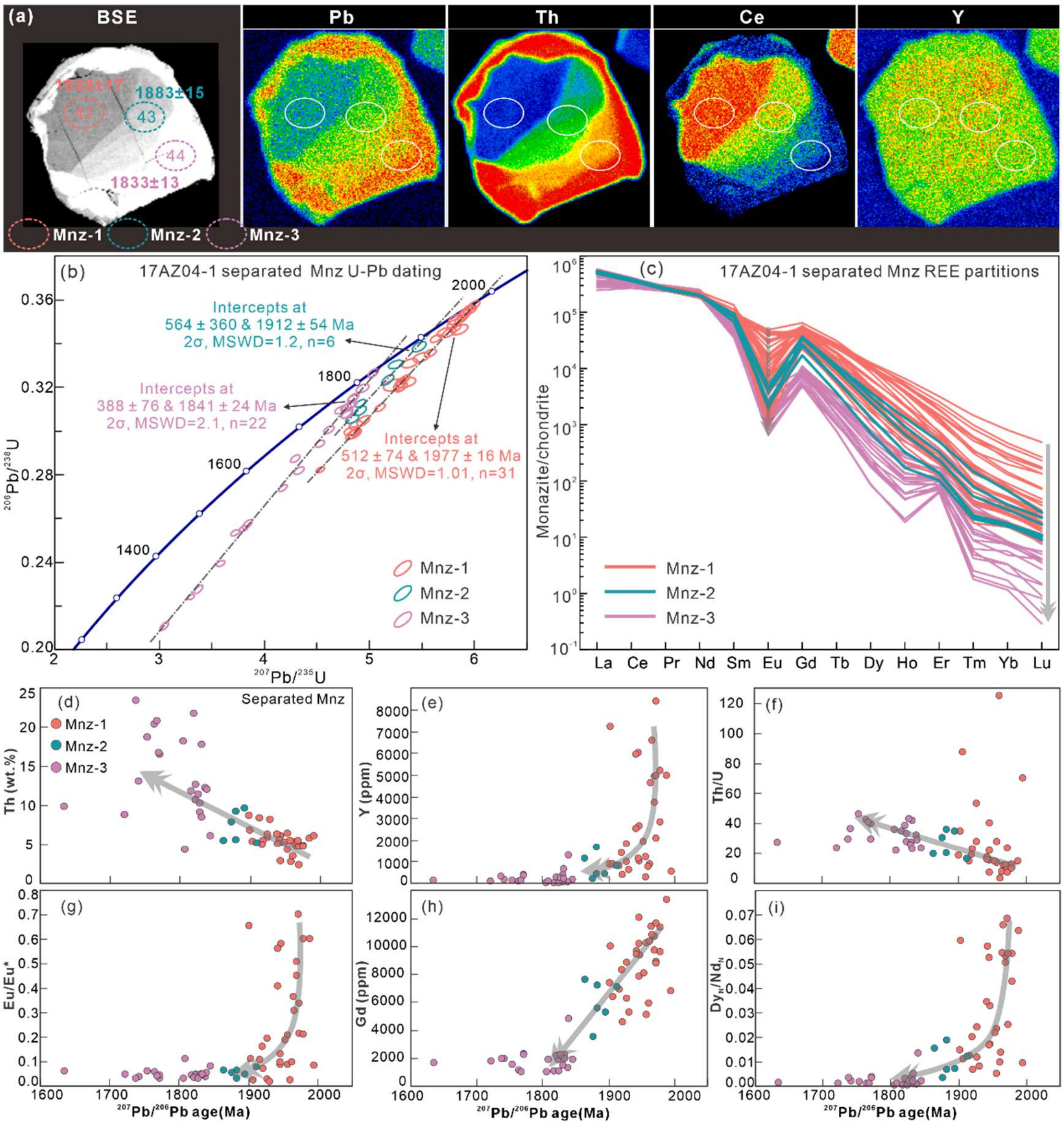

Supplementary Figure 8. The elemental mapping images, geochronology, and geochemistry results from the separated monazite. (a) The BSE images and the X-ray mapping of elements (Pb, Th, Ce, and Y) for separated monazite from the pelitic granulite samples, showing three types of microdomains (Mnz-1, Mnz-2, and Mnz-3); (b–c) Concordia diagram and Chondrite-normalised REE patterns of the separated three-types monazites U-Pb dating; (d–i) Compositional characteristics of the separated three-types monazite microdomains from the pelitic granulite samples. The arrows represent the variation trend of chemical composition with reducing $^{207}Pb/^{206}Pb$ ages.

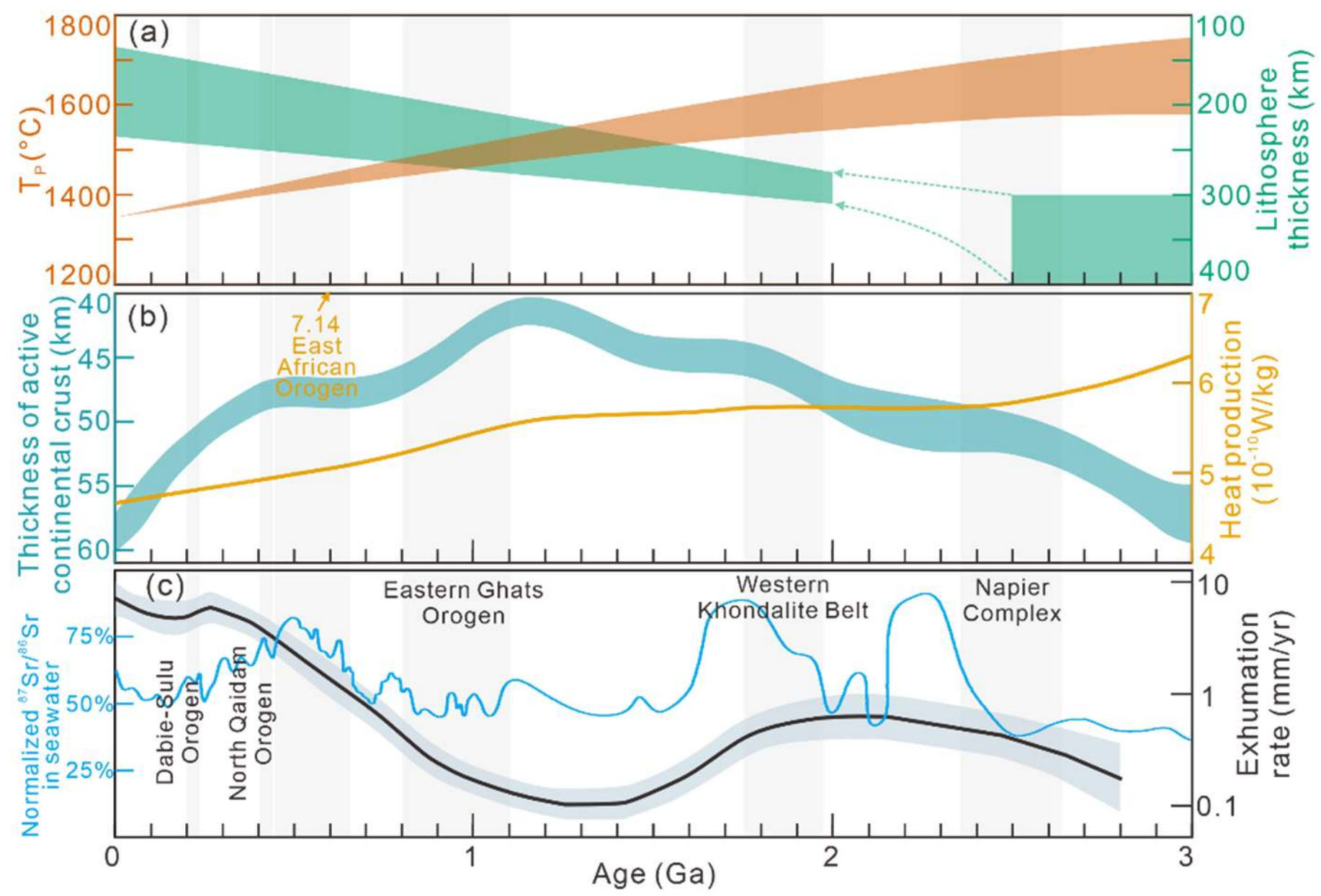

Supplementary Figure 9. Variation trend curves of various geological parameters. Secular trends of the mantle potential temperature[17] and lithosphere thickness[18] (a), the thickness of active continental crust[19] and heat production[20] (b), and the exhumation rate[21] and normalized $^{87}Sr/^{86}Sr$ in seawater[22] (c).

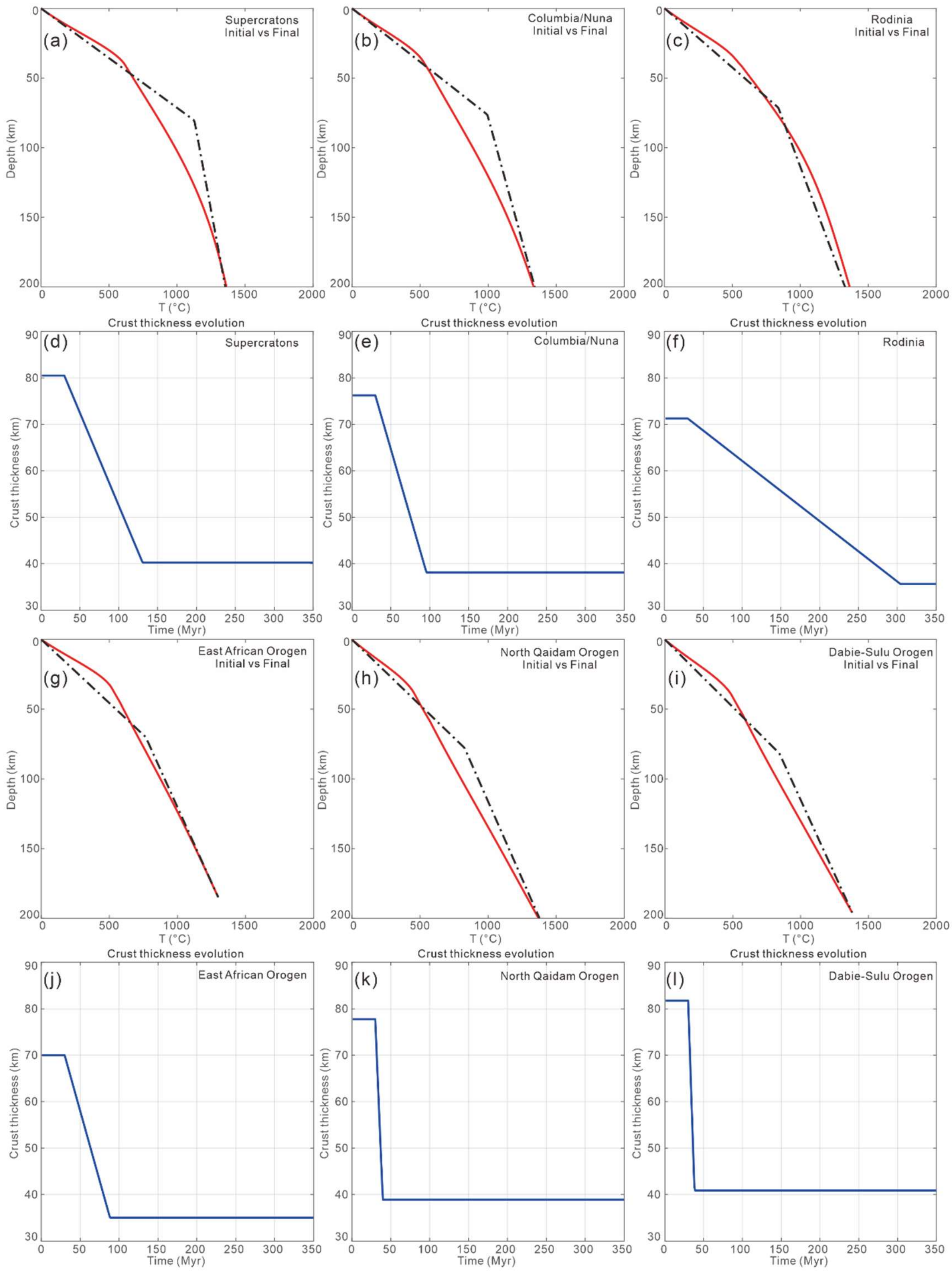

Supplementary Figure 10. Thermal and crustal thickness evolution modelled the Precambrian hot orogens and Phanerozoic cold orogens.

Supplementary References

1 Zou, L. *et al.* Paleoproterozoic ultrahigh-temperature metamorphism in the Alxa Block, the Khondalite Belt, North China Craton: Petrology and phase equilibria of quartz-absent corundum-bearing pelitic granulites. *Journal of Metamorphic Geology* **40**, 1159-1187 (2022). https://doi.org/10.1111/jmg.12661

2 Otamendi, J. E., de la Rosa, J. D., Douce, A. E. P. & Castro, A. Rayleigh fractionation of heavy rare earths and yttrium during metamorphic garnet growth. *Geology* **30**, 159-162 (2002). https://doi.org/10.1130/0091-7613(2002)030<0159:Rfohre>2.0.Co;2

3 Rubatto, D. *et al.* Identification of growth mechanisms in metamorphic garnet by high-resolution trace element mapping with LA-ICP-TOFMS. *Contributions to Mineralogy and Petrology* **175** (2020). https://doi.org/10.1007/s00410-020-01700-5

4 Nash, W. P. & Crecraft, H. R. Partition-coefficients for trace-elements in silicic magmas. *Geochimica et Cosmochimica Acta* **49**, 2309-2322 (1985). https://doi.org/10.1016/0016-7037(85)90231-5

5 Hermann, J. & Rubatto, D. Relating zircon and monazite domains to garnet growth zones: age and duration of granulite facies metamorphism in the Val Malenco lower crust. *Journal of Metamorphic Geology* **21**, 833-852 (2003). https://doi.org/10.1046/j.1525-1314.2003.00484.x

6 Spear, F. S. & Kohn, M. J. Trace element zoning in garnet as a monitor of crustal melting. *Geology* **24**, 1099-1102 (1996). https://doi.org/10.1130/0091-7613(1996)024<1099:Teziga>2.3.Co;2

7 Lebreton, N. & Thompson, A. B. Fluid-absent (dehydration) melting of biotite in metapelites in the early stages of crustal anatexis. *Contributions to Mineralogy and Petrology* **99**, 226-237 (1988).

8 Kelsey, D. E., Clark, C. & Hand, M. Thermobarometric modelling of zircon and monazite growth in melt-bearing systems: examples using model metapelitic and metapsammitic granulites. *Journal of Metamorphic Geology* **26**, 199-212 (2008). https://doi.org/10.1111/j.1525-1314.2007.00757.x

9 Jiao, S. *et al.* Establishing the P-T path of UHT granulites by geochemically distinguishing peritectic from retrograde garnet. *American Mineralogist* **106**, 1640-1653 (2021). https://doi.org/10.2138/am-2021-7681

10 Cao, X. *et al.* Earth's tectonic and plate boundary evolution over 1.8 billion years. *Geoscience Frontiers* **15** (2024). https://doi.org/10.1016/j.gsf.2024.101922

11 Zhao, G. C., Sun, M., Wilde, S. A. & Li, S. Z. Late Archean to Paleoproterozoic evolution of the North China Craton: key issues revisited. *Precambrian Research* **136**, 177-202 (2005). https://doi.org/10.1016/j.precamres.2004.10.002

12 Zhang, J., Gong, J., Yu, S., Li, H. & Hou, K. Neoarchean-Paleoproterozoic multiple tectonothermal events in the western Alxa block, North China Craton and their geological implication: Evidence from zircon U-Pb ages and Hf isotopic composition. *Precambrian Research* **235**, 36-57 (2013). https://doi.org/10.1016/j.precamres.2013.05.002

13 Zou, L. *et al.* Paleoproterozoic ultrahigh-temperature mafic granulites with a high-pressure prograde path from the Alxa Block: Implications on the tectonic evolution of

the Khondalite Belt, North China Craton. *Journal of Metamorphic Geology* **42**, 551-581 (2024). https://doi.org/10.1111/jmg.12764

14 McDonough, W. F. & Sun, S. s. The composition of the Earth. *Chemical Geology* **120**, 223-253 (1995). https://doi.org/https://doi.org/10.1016/0009-2541(94)00140-4

15 Dodson, M. H. Closure Temperature in Cooling Geochronological and Petrological Systems. *Contributions to Mineralogy and Petrology* **40**, 259-274 (1973).

16 Bloch, E. & Ganguly, J. ^{176}Lu–^{176}Hf geochronology of garnet II: numerical simulations of the development of garnet–whole-rock ^{176}Lu–^{176}Hf isochrons and a new method for constraining the thermal history of metamorphic rocks. *Contributions to Mineralogy and Petrology* **169** (2015). https://doi.org/10.1007/s00410-015-1115-x

17 Herzberg, C., Condie, K. & Korenaga, J. Thermal history of the Earth and its petrological expression. *Earth and Planetary Science Letters* **292**, 79-88 (2010). https://doi.org/10.1016/j.epsl.2010.01.022

18 Sun, C. & Dasgupta, R. Thermobarometry of CO_2-rich, silica-undersaturated melts constrains cratonic lithosphere thinning through time in areas of kimberlitic magmatism. *Earth and Planetary Science Letters* **550** (2020). https://doi.org/10.1016/j.epsl.2020.116549

19 Tang, M., Chu, X., Hao, J. & Shen, B. Orogenic quiescence in Earth's middle age. *Science* **371**, 728-731 (2021). https://doi.org/10.1126/science.abf1876

20 Ptacek, M. R., Dauphas, N. & Greber, N. D. Chemical evolution of the continental crust from a data-driven inversion of terrigenous sediment compositions. *Earth and Planetary Science Letters* **539** (2020). https://doi.org/10.1016/j.epsl.2020.116090

21 Zou, Y. *et al.* Surface evolution during the mid-Proterozoic stalled by mantle warming under Columbia-Rodinia. *Earth and Planetary Science Letters* **607** (2023). https://doi.org/10.1016/j.epsl.2023.118055

22 Chen, X., Zhou, Y. & Shields, G. A. Progress towards an improved Precambrian seawater ^{87}Sr/^{86}Sr curve. *Earth-Science Reviews* **224**, 103869 (2022). https://doi.org/https://doi.org/10.1016/j.earscirev.2021.103869
