# Supplementary material for: Secular change in orogenic duration reveals alternating tectonic regimes during the Archaean-Proterozoic
Source: Nat Commun. 2026 Aug 11;17:9621. doi: 10.1038/s41467-026-76530-3 (PMC13554081; doi:10.1038/s41467-026-76530-3)
Supplement: Supplementary file 2 [file 41467_2026_76530_MOESM2_ESM.pdf]

Description of Additional Supplementary Information

Supplementary Data 1: Trace elements' compositions of garnet from the pelitic granulite samples.

Supplementary Data 2: LA-ICPMS/MS garnet Lu-Hf isotopic data for the pelitic granulite samples.

Supplementary Data 3: LA-ICP-MS U-Pb isotopic data and REE compositions of the separated monazite from the pelitic granulite samples.

Supplementary Data 4: LA-ICP-MS U-Pb isotopic data and REE compositions of the texturally controlled monazite from the pelitic granulite samples.

Supplementary Data 5: Secular thermal and temporal evolution for the Precambrian hot orogens and Phanerozoic orogens.

Supplementary Data 6: The parameters used for the 1D thermal modelling.
