# Supplementary material for: Secular change in orogenic duration reveals alternating tectonic regimes during the Archaean-Proterozoic
Source: Nat Commun. 2026 Aug 11;17:9621. doi: 10.1038/s41467-026-76530-3 (PMC13554081; doi:10.1038/s41467-026-76530-3)
Supplement: Supplementary file 3 [file 41467_2026_76530_MOESM3_ESM.pdf]

nature portfolio

Peer Review File

Secular change in orogenic duration reveals alternating tectonic regimes during the Archaean-Proterozoic

Corresponding Author: Dr Guangyu Huang

This file contains all reviewer reports in order by version, followed by all author rebuttals in order by version.

Version 0:

Reviewer comments:

Reviewer #1

(Remarks to the Author)
Journal: Nature Communications
Manuscript Number: NCOMMS-19-02747
Secular change of orogenic duration reveals “alternating” tectonic regimes during the Archaean-Proterozoic by Lei Zou et al.
Reviewer: K. Sajeev
The study investigates the temporal evolution of collisional orogens from the Neoarchaean to the Neoproterozoic using high-precision in-situ garnet Lu–Hf and monazite U–Pb geochronology applied to ultrahigh-temperature (UHT) granulites from the Khondalite Belt, North China Craton. The authors constrain a ~220 million year-long Paleoproterozoic collisional cycle during the Columbia/Nuna assembly, substantially exceeding the duration of a typical cold orogen, which is about 120–160 million years. Through synthesis of global datasets and one-dimensional thermal modeling, they identify a non-monotonic evolution in orogenic duration that correlates with alternating phases of lithospheric plate and plate mobility. The paper proposes an alternating tectonic regime that operated during the Neoarchaean and Mesoproterozoic, became very active during the Palaeoproterozoic (Columbia/Nuna) and Neoproterozoic (Rodinia breakup), marking progressive establishment of modern-style plate tectonics.
Major Comments
Conceptual strength
The integration of in situ Lu–Hf and microdomain monazite dating on UHT granulites is technically rigorous and globally relevant. The linkage between orogenic duration and plate-tectonic processes is innovative and impactful for deep-time geodynamics. However, the term alternating tectonic regime would benefit from a clearer mechanistic definition. Is alternation a reflection of mantle-driven oscillations or lithospheric coupling parameters that need to be explained? Explicitly relate this to the cited models those are discussed in the manuscript.
The garnet Lu–Hf system yields closure at ~950–1000 °C, but uncertainties in diffusion parameters and the possibility of partial resetting during UHT conditions should be quantitatively evaluated. A diffusion modeling figure or sensitivity analysis for Hf diffusion with respect to grain size and temperature would strengthen the reliability of the 1.99–1.98 Ga prograde age.
The textural linkage between Mnz-1/2/3 and garnet zones is compelling but requires explicit back-scatter and element maps (supplementary figures S4–S5 are too small). Request clearer cross-referenced photomicrographs showing monazite domains in context.
Duration estimates and statistical compilation
The compiled age v.s. The global supercraton cycles (Figure 3) dataset integration may have precision issues due to variations in the closure temperatures and chronometers. The paper claims consistent geochronological standards, but methodological heterogeneity will persist (zircon U–Pb vs. monazite U–Th–Pb vs. garnet Lu–Hf). It is very useful to provide a Supplementary Table listing all included studies, including the closure temperature and mineral system, to demonstrate comparability.
In Figure 5a, for orogenic duration trends, quantify statistical variance and confidence bands, or use box-and-whisker plots rather than mean lines alone.
Thermal modeling
The 1-D thermal model (using Equation 1) reproduces general duration patterns, but its boundary assumptions, like instantaneous crustal doubling or fixed basal temperature, are highly idealized. The authors should discuss the limitations of their approach relative to more realistic 2-D thermo-mechanical models (e.g., Sizova 2014). Also, clarify the calibration: how were the erosion rates in Figure S7 chosen and validated against geological proxies?
Figures 4g–h test mantle-temperature perturbations of 100 °C, yet the authors conclude these are very limited. Quantify the

effect magnitude numerically in the caption and discuss sensitivity to radiogenic heat production.
Interpretation and overreach
The extrapolation from orogenic duration to plate velocity and plateness in Figure 5b is speculative. The correlation is plausible but not loosely proven. Suggest rephrasing: ‘implies enhanced plateness consistent with inferred plate-velocity proxies’ rather than asserting direct evidence.
Likewise, the claim of ‘proto-modern plate tectonics’ during Columbia assembly (see Abstract lines 42–44) should be modified unless independent kinematic or paleomagnetic constraints are cited.
Figure modifications:
Figure 2: Add clear subpanel lettering (a–c) and enlarge Lu–Hf isochron plots for readability.
Figure 3: Use a uniform temporal scale; mark transitions between Kenor, Columbia, Rodinia, Gondwana, and Phanerozoic clearly on the x-axis.
Figure 4: Label simulated temperature curves with parameter sets (erosion rate, crustal thickness).
Figure 5: Add uncertainty shading and specify data sources for plate velocities (O’Neill 2022).
I am recommending a moderate revision and hope the manuscript will be eventually accepted and published in Nature Communications,
Best Wishes

Reviewer #2

(Remarks to the Author)
Thanks for the opportunity to read and comment on this interesting manuscript. I congratulate the authors on putting together a good data-focussed paper and then using this as a step to look at the larger Earth system. It is always difficult to justify using a single example as a general case for a wider Earth system and the authors have attempted this well, although not totally convincingly in my opinion. As you will see form the comments below and the annotations on the manuscript, the supplementary figures and the supplementary text attached, I suggest that:
1) the data can be interpreted just as convincingly (i would suggest more convincingly) as a shorter duration event, or possibly a number of events..
2) The errors need to be quoted at a 2 standard deviation level - by quoting them at 1 standard deviation it makes them look more precise than they are.
3) I'm afraid that I am not convinced by the age ranges of the orogens that are discussed. These are all quite arbitary and when looked at in detail become much more complicated and much more similar to Phanerozoic orogens.
4) Please use more recent reconstructions of supercontinents and I suggest using full-plate models such as Cao et al. 2024 - for consistency and because these are much more constrained than the old continental-drift models.

Specific points (more are in the annotated files)

Lines 69-71
I'm not aware of any regions of this sort of size that show pervasive UHT metamorphism. UHT metamorphism is restricted to small regions where the indicative minerals have been recognised. It is possible that large areas have been effected by these extreme temperatures, but the mineralogy of these regions does not preserve this. Despite the claims in Jiao et al 2023, the Musgraves orogen, the Eastern Ghats Orogen and most of the other <500 km2 orogens they list, do not have demonstrated evidence for UHT metamorphism over the area scale that are quoted - others - e.g. Holder et al for the Anoysyen Domain, are associated with extensive magmatism advecting heat into the orogen.

Despite the claims in Jiao et al 2023, the Musgraves orogen, the Eastern Ghats Orogen and most of the other <500 km2 orogens they list, do not have demonstrated evidence for UHT metamorphism over the area scale that are quoted - others - e.g. Holder et al for the Anoysyen Domain, are associated with extensive magmatism advecting heat into the orogen. The garnet core likely grew over these pre-garnet monazites.

Line 84
I think it only fair that you refer to Simpson et al. 2021 here - as they pioneered this technique - https://www.sciencedirect.com/science/article/pii/S0009254121002436

Line 131
I would suggest that the high HREE concentrations here suggest that the monazite wasn't growing in competition with garnet... so perhaps the garnet grew later (note the large errors on the garnet ages making this plausible). The garnet core likely grew over these pre-garnet monazites.

Lines 141-143
I'm afraid that i don't see that your data can really justify this - you have early garnet that is probably most precisely dated by the M2 monazites at 1917 +/- 52 Ma (2 s.d.) growing over pre-garnet monazites. Then a later garnet growth at ca. 1800 Ma [not super well constrained as many of the monazites show Pb loss and mixed zones - see fig. S5a here for a nice example].

Lines 151-154
Yes, but, as the study above suggests, these minerals often grow sub-closure temperature - meaning that the ages do not directly reflect the closure temperature.

Lines 180-181
I'm not quite sure how this is calculated - there is accretionary orogenesis here lasting from 1080 Ma to 650 Ma when there is

a major collision and crustal thickening - then a second collision and crustal thickening at ca. 580 Ma with peak UHT metamorphism at ca. 550 Ma and cooling to ca. 500 degrees by 520 Ma.

Line 196
So looking at the parameters used a major control is the thickness of crust that you take from Tang et al. 2021. Note that this paper excludes S-type granites, which means that continent-continent collisional orogens are excluded from the dataset! i.e. the modern Himalaya would not be visible here.

Line 226
Note that with full-plate tectonic reconstructions you don't see any notable slow down - e.g. Cao et al 2014 - Figure 15. The confusion is that previous studies are based on continental drift models that only consider continents.

Lines 258
Rather than 'incomplete' break up - I think more accurate is that the breakup didn't lead to an extraversion - with break up of Laurentia and Australia/NChina/EAntarctica then forming an ocean that closed by introversion... see Cao et al. 2024

Line 261-263
The plate velocities that you have in your model are just continent plates, these will not be affected by friction changes at the margins of the supercontinent - as the plates affected are the oceanic plates being subducted. I don;t think that this line is needed here.

Fig 1
The plate velocities that you have in your model are just continent plates, these will not be affected by friction changes at the margins of the supercontinent - as the plates affected are the oceanic plates being subducted. I don;t think that this line is needed here.

Fig 2c
This spread of data could also be interpreted simply as ca. 1986 Ma monazite that lost radiogenic Pb at ca. 1800 Ma [or even with mixed analyses of different monazite domains recording these two ages]

Fig. 3
I'm afraid that I would argue that most of these time lines are gross simplifications - sorry... For example, the Eastern Ghats. There is very little evidence for pre-950 Ma UHT metamorphism (a few monazites in inclusions) and extremely little evidence for the so-called heating stage.
Also, the East African orogen, at 650 Ma there was an earlier continental-collisional orogeny (the East African Orogeny) - there was basin formation (extension?) between that and the subsequent collision at ca. 580 Ma (the Malagasy Orogeny) that caused UHT metamorphism at ca. 550 Ma.

Fig. 5
Note that Cao et al. 2024 have an updated plate velocity curve based on a full earth full-plate tectonic model - this model you have here is based on continent velocities only.

Fig S03
I'm not sure what has happened to Mnz-4 here - firstly, the colours don't seem to match the legend (brown versus orange). Secondly, you seem to suggest that the Mnz-4 here has the largest Eu anomaly, yet in figure (f) below, Mnz-4 has no Eu anomaly??? While Mnz-1 has the largest anomaly - that is NOT what this shows here....

Fig S04
It is interesting how this monazite has itself a core and rim even though it is contained as an inclusion in the garnet mantle....

Also, you can see the 'ghost' of the original extent of the early monazite in the Y and Th maps - suggesting that you have mixed ages in your data.

Fig S05a
I think another interpretation of these images and data are that this reflects an early monazite age of ca. 1930 Ma that is then overprinted by a later monazite of ca. 1830 Ma with a mixed region between the two - this looks pretty clear with the Pb map in particular.... In fact in the Pb, Th and Ce maps, it looks like you can see the early monazite 'ghost' in preserved even below analysis spot 44 - suggesting that the 'real' later age is probably younger than 1830 Ma... and represented by the deep red (Th) and blue (Ce maps)

Fig S05c and g
Similar issue here - increasing Eu anomaly from Mnz-1 to Mnz-3 yet this is plotted inversely in (g).

Regards
Alan Collins

Reviewer #3

(Remarks to the Author)

The manuscript by Zou et al. uses new (monazite U-Pb and garnet Lu-Hf) and compiled data to study hot-orogens in the Paleoproterozoic. The text is well written, and the figures and data are of great quality.

Below are some comments:

1) Fig. 1: Numbers plotted in the figure are not mentioned in the legend. What do the numbers 1, 2, 3, 19, etc. mean?

2) Lines 141 – 142: "Collision age at ca. 1.99 – 1.98 Ga progressing to peak conditions at ca. 1.96 – 1.95 Ga". I'm not sure where these ages came from, since they do not match the garnet Lu-Hf or monazite U-Pb ages.

I'm guessing 1.96 – 1.95 Ga is the age of zircons previously cited, but I'm not sure.

This part needs some rewriting to make this discussion and interpretation clearer and based on literature and/or the new data exposed in the text.

3) Fig. S6 / Line 193: Fig. 6 uses crust thickness based on Eu anomalies (Tang et al., 2021).

This is a problem that is well constrained in the literature now.

Eu anomalies are controlled by plagioclase proportion and are a function of pressure, temperature, rock composition, and changes in oxygen fugacity.

So now we know that Eu anomalies in zircon should not be used as a proxy for crustal thickness (Yakymchuk et al., 2023).

If the authors used this proxy to model the results shown in Figure 4 and Figure S07, they should acknowledge this important limitation. I personally believe that the authors should not use Eu anomalies to estimate crustal thickness and should not use these results for modelling. Using the Eu anomaly proxy could yield a wrong model, and, subsequently, all results and discussions will be questionable.

Yakymchuk, C., Holder, R. M., Kendrick, J., & Moyen, J. F. (2023). Europium anomalies in zircon: A signal of crustal depth?. Earth and Planetary Science Letters, 622, 118405. https://doi.org/10.1016/j.epsl.2023.118405

4) Line 234 – 238: Huang et al (2026) dated eclogite (low T/P) in the transition between the Mesoarchean and Neoarchean - 1.5 – 2.5 GPa and temperatures of 580 – 660 °C (low T/P conditions of 230 – 440 °C/GPa).
Also, there are the classical Brown and Johnson graphs (and other authors as well; https://doi.org/10.1144/jgs2024-091?ref=pdf&rel=cite-as&jav=VoR; https://doi.org/10.1016/j.precamres.2023.107195; https://doi.org/10.1038/s41598-024-56691-1) compiling high, moderate, and low T/P data from the metamorphic record worldwide. They have shown a series of low T/P eclogites in the Paleoproterozoic and even in the Archean.
Also, potential evidence of low T/P metamorphism before ca. 2.5 Ga can be also found in North China (Ning et al., 2022), and Bundelkhand Craton (India; Saha et al., 2011)
All these information should be acknowledged in the manuscript. Will this change the interpretations of this manuscript?

5) Line 286: "Alternating tectonic regime evolution". It is not clear why the authors concluded this. The authors did not mention low T/P (eclogite) data in the Archean/Paleoproterozoic to support these alternating tectonic regimes.
I believe this interpretation is based on the existence of low T/P (i.e., plate tectonics / mobile-lid) between periods of hot orogenesis. But this is not clear through the text: several authors dated low T/P rocks in the Archean/Paleoproterozoic.

Thank you!

Best regards

Version 1:

Reviewer comments:

Reviewer #2

(Remarks to the Author)
Thanks for considering all the points I made in the original review. I'm more than satisfied with your responses and

congratulate you on putting together an excellent, and usefully controversial, paper.

Reviewer #3

(Remarks to the Author)
Thanks for the great opportunity to read, comment, and contribute a little bit to this manuscript. In my opinion, this manuscript is in great shape and quality and ready for publication.

The main concerns raised in the first round of revision were all carefully addressed and inserted in the manuscript.

I acknowledge and congratulate the authors for putting together a great paper and use it as an example to understand a broader problem in the geosciences.

Best wishes

Rodrigo Irineu Cerri

Open Access This Peer Review File is licensed under a Creative Commons Attribution 4.0 International License, which permits use, sharing, adaptation, distribution and reproduction in any medium or format, as long as you give appropriate credit to the original author(s) and the source, provide a link to the Creative Commons license, and indicate if changes were made.
In cases where reviewers are anonymous, credit should be given to 'Anonymous Referee' and the source.
The images or other third party material in this Peer Review File are included in the article's Creative Commons license, unless indicated otherwise in a credit line to the material. If material is not included in the article's Creative Commons license and your intended use is not permitted by statutory regulation or exceeds the permitted use, you will need to obtain permission directly from the copyright holder.
To view a copy of this license, visit https://creativecommons.org/licenses/by/4.0/

Responses in blue.

“**Revisions** in red”.

Line numbers refer to “Revised manuscript with tracked changes”.

Reviewer #1 (Remarks to the Author):

Secular change of orogenic duration reveals “alternating” tectonic regimes during the Archaean-Proterozoic by Lei Zou et al.
Reviewer: K. Sajeev
The study investigates the temporal evolution of collisional orogens from the Neoarchaean to the Neoproterozoic using high-precision in-situ garnet Lu–Hf and monazite U–Pb geochronology applied to ultrahigh-temperature (UHT) granulites from the Khondalite Belt, North China Craton. The authors constrain a ~220 million year-long Paleoproterozoic collisional cycle during the Columbia/Nuna assembly, substantially exceeding the duration of a typical cold orogen, which is about 120–160 million years. Through synthesis of global datasets and one-dimensional thermal modeling, they identify a non-monotonic evolution in orogenic duration that correlates with alternating phases of lithospheric plate and plate mobility. The paper proposes an alternating tectonic regime that operated during the Neoarchaean and Mesoproterozoic, became very active during the Palaeoproterozoic (Columbia/Nuna) and Neoproterozoic (Rodinia breakup), marking progressive establishment of modern-style plate tectonics.

Major Comments

Conceptual strength

Comment 1.1

(1) The integration of in situ Lu–Hf and microdomain monazite dating on UHT granulites is technically rigorous and globally relevant. The linkage between orogenic duration and plate-tectonic processes is innovative and impactful for deep-time geodynamics. However, the term alternating tectonic regime would benefit from a clearer mechanistic definition. Is alternation a reflection of mantle-driven oscillations or lithospheric coupling parameters that need to be explained? Explicitly relate this to the cited models those are discussed in the manuscript.

Thank you for your constructive comment. We fully agree that the term regarding “alternating tectonic regime” requires a clearer definition and elaboration for readers’ understanding. Accordingly, we have added an explanation and clarification of this tectonic regime in the Discussion section of the manuscript (Lines 234–250).

“This alternating thermal evolution is likely a response to changing lithospheric coupling parameters such as the plateness and mobility (e.g., Fig. 5), which are an overall expression of the tectonic regimes under which the Earth has operated through its history[1]. Plateness refers to the degree of rigidity of tectonic plates[1,2]. Plate tectonics is characterized by high degrees of plateness (i.e., rigid plates with deformation focused at plate boundaries). In contrast, non-plate tectonic mobile lid regimes (e.g., sluggish lid) may involve broader zones of plate deformation, and hence lower plateness. Sluggish lid regimes will be facilitated by magmatism and UHT metamorphism, which will decrease, at

least locally, plate rigidity[1,69]. This will also likely lead to non-uniform rates of plate motion. Thus, the non-monotonic evolution of orogenic belts can be viewed in response to changing plateness and mobility, leading to an alternation between sluggish and active plate-tectonic states. The Neoarchean is characterized by likely sluggish lid setting progressing to an active plate tectonic phase in the Paleoproterozoic, then a return to sluggishness in the Mesoproterozoic, which corresponds with Earth's Middle Age[70], and finally a transition to contemporary active plate tectonics from the Neoproterozoic into the Phanerozoic."

Comment 1.2

(2) The garnet Lu–Hf system yields closure at ~950–1000 °C, but uncertainties in diffusion parameters and the possibility of partial resetting during UHT conditions should be quantitatively evaluated. A diffusion modeling figure or sensitivity analysis for Hf diffusion with respect to grain size and temperature would strengthen the reliability of the 1.99–1.98 Ga prograde age.

Thank you for your comment. Indeed, the issue you raised is discussed in the Supplementary Figure (Fig. S02d) of our article. However, we recognize that this point was not clearly articulated. Accordingly, we have made appropriate modifications to address it (Lines 126–127).

"(ca. 950–1000 °C closure temperature, considering garnet grain radius of 2–4 mm; Fig. S2d)"

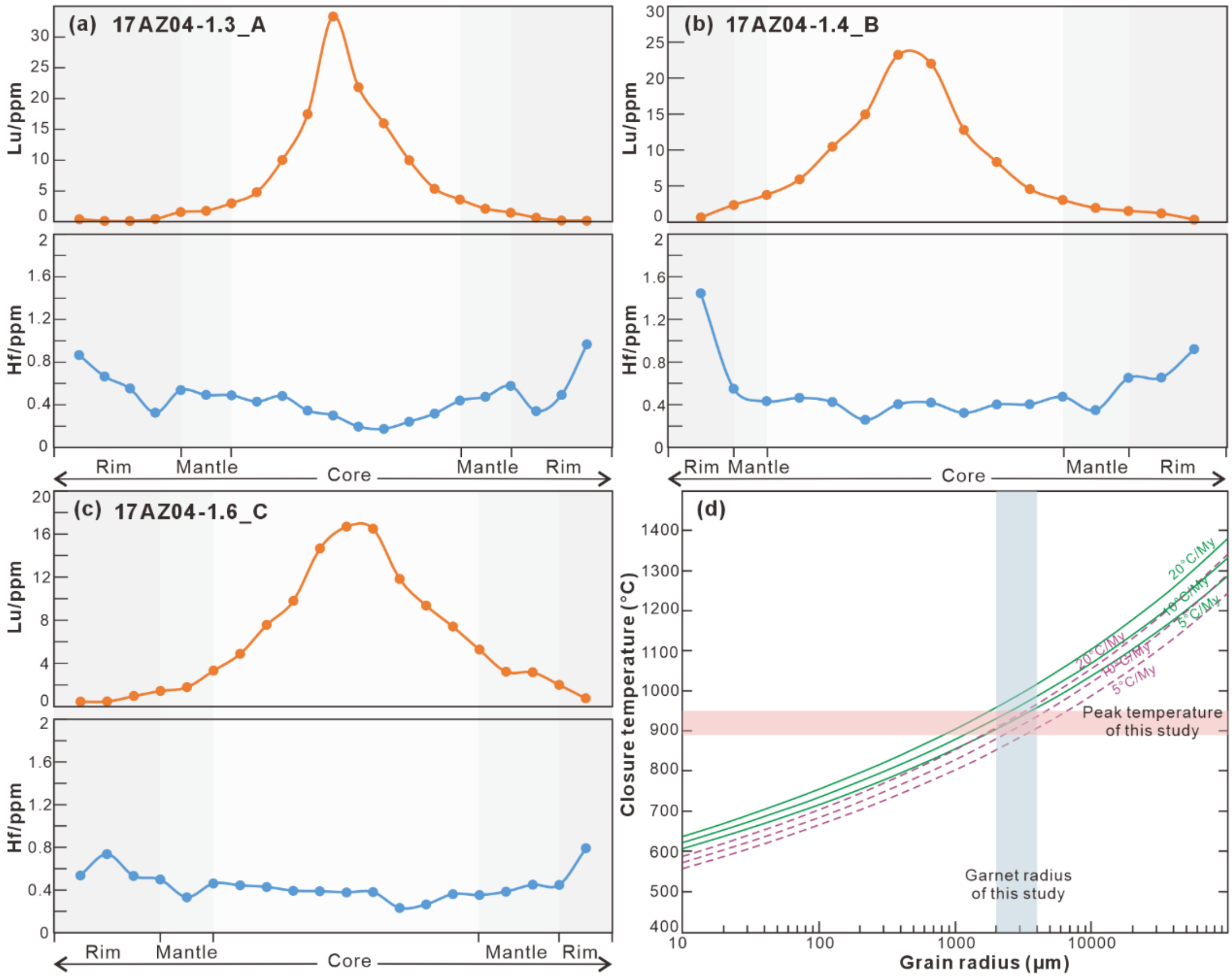

Comment 1.3

(3) The textural linkage between Mnz-1/2/3 and garnet zones is compelling but requires explicit back-scatter and element maps (supplementary figures S4–S5 are too small). Request clearer cross-referenced photomicrographs showing monazite domains in context.

Thank you for your comment. We recognize that the structural context of monazite within garnet, as shown in the supplementary figure, is too small for readers to clearly and intuitively discern its positional relationship with garnet zones. To address this, we have included the corresponding backscattered electron (BSE) images in Supplementary Figure S4, which more clearly display the location of monazite both within garnet and in the surrounding matrix.

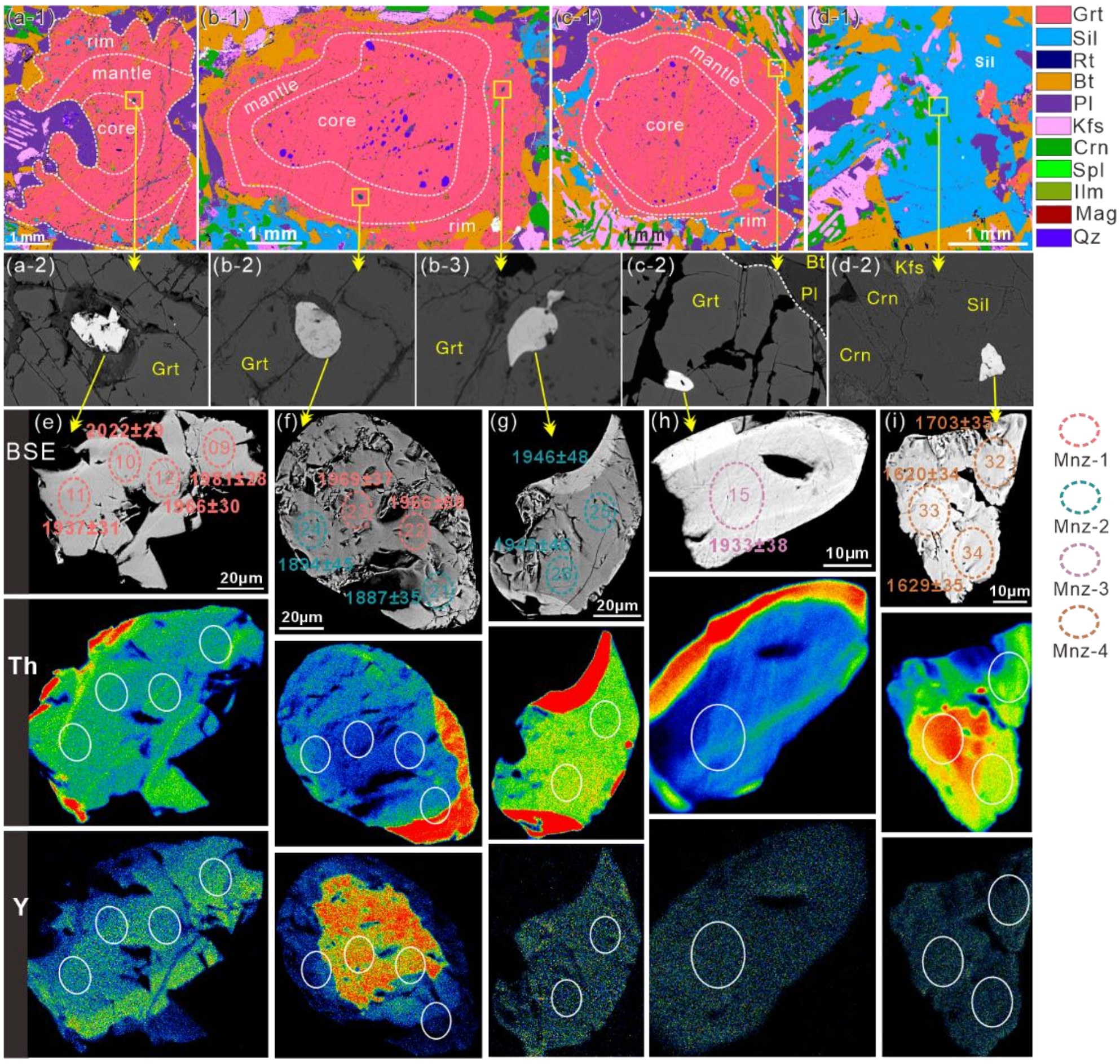

Duration estimates and statistical compilation

Comment 1.4

(1) The compiled age v.s. The global supercraton cycles (Figure 3) dataset integration may have precision issues due to variations in the closure temperatures and chronometers. The paper claims consistent geochronological standards, but methodological heterogeneity will persist (zircon U–Pb vs. monazite U–Th–Pb vs. garnet Lu–Hf). It is very useful to provide a Supplementary Table listing all included studies, including the closure temperature and mineral system, to demonstrate comparability.

We fully agree with your perspective. When compiling the geochronological data from global orogenic belts across supercontinent cycles, the closure temperatures recorded by different mineral isotopic systems may vary. Additionally, as highlighted by Reviewer #2,

zircon and monazite often continue to grow below their closure temperatures, recording ages corresponding to lower-temperature conditions. These discrepancies are closely relevant to the timescales and thermal evolution processes of the orogenic belts discussed in our study. Therefore, based on the original references and understanding of their geochronological data, we have provided a detailed compilation in Supplementary Table 5, listing the mineral systems used to constrain each orogenic timescale along with their corresponding closure or growth temperatures. This allows us to demonstrate the consistency among these datasets and properly evaluate their comparability.

Comment 1.5

(2) In Figure 5a, for orogenic duration trends, quantify statistical variance and confidence bands, or use box-and-whisker plots rather than mean lines alone.

Thank you very much for your constructive comment. We have revised the figure, presenting the orogenic belt timescale data for each supercontinent assembly period using box-and-whisker plots.

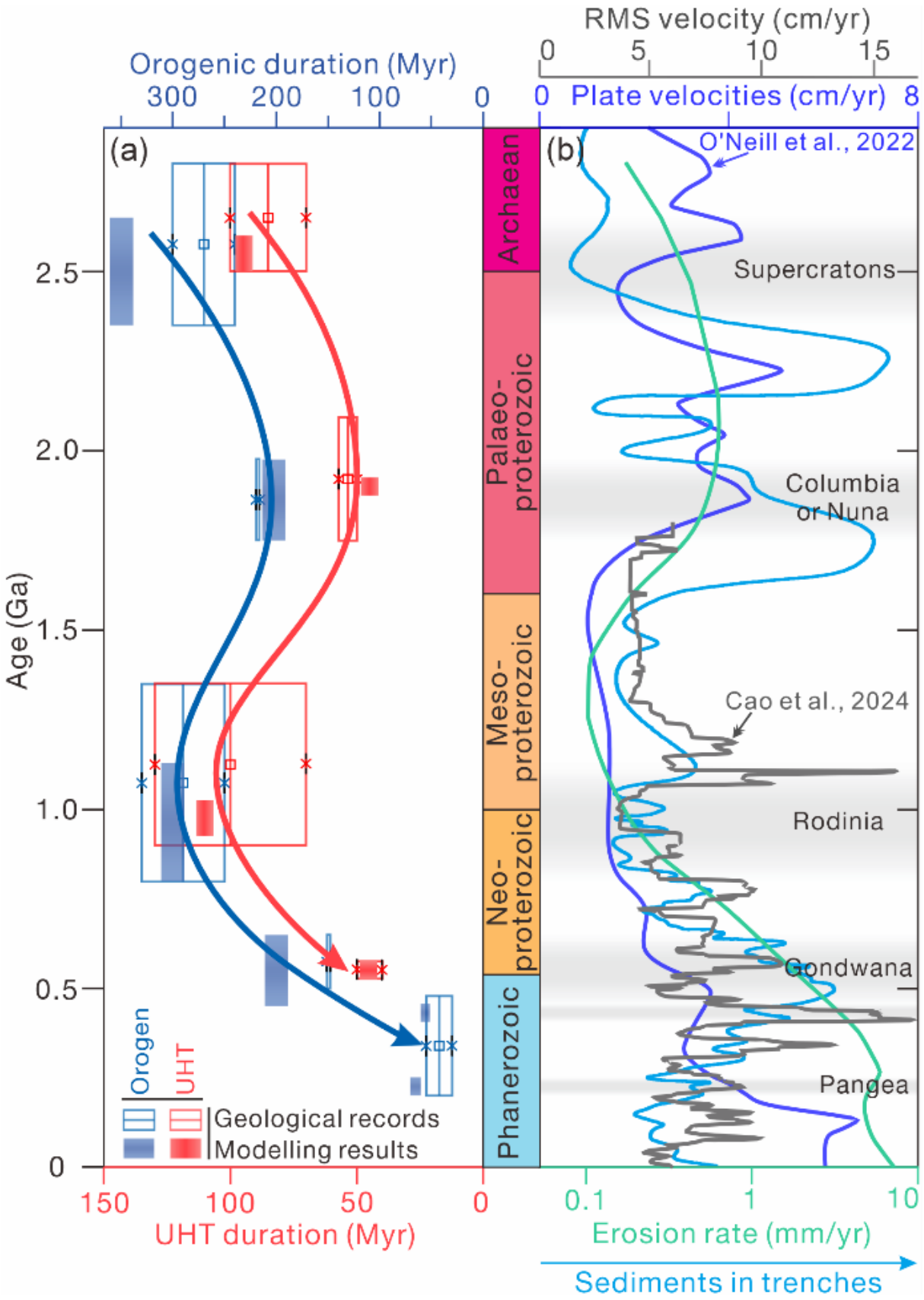

Thermal modeling

Comment 1.6

(1) The 1-D thermal model (using Equation 1) reproduces general duration patterns, but its boundary assumptions, like instantaneous crustal doubling or fixed basal temperature, are highly idealized. The authors should discuss the limitations of their approach relative to more realistic 2-D thermo-mechanical models (e.g., Sizova 2014).

Indeed, as you pointed out, some of the boundary conditions in our 1D thermal modeling are idealized. Compared to 2D thermos-mechanical models, the inherent characteristics of this method inevitably introduce certain limitations to the simulation results. Accordingly, we have added a relevant discussion in the manuscript (Lines 398–409).

"The 1D thermal modelling is simplified to capture the first-order thermal evolution of a vertically thickened lithospheric column during collision and erosion. Assumptions including instantaneous crustal thickening and a fixed basal temperature are idealizations and do not represent the full thermos-mechanical complexity of collisional orogenesis. Compared to 1D modelling, the 2D thermos-mechanical models (e.g., ref. [7]) can explicitly resolve lateral heat transfer, evolving collision geometry, spontaneous convergence, slab breakoff, and exhumation processes. However, our approach is not intended to reconstruct the full dynamics of orogens, but to provide a consistent comparative framework for evaluating how secular variations in lithospheric structure, basal thermal conditions, radiogenic heat production, and erosion rate affect the duration of elevated thermal conditions. The model results are therefore interpreted as first-order thermal scaling relationships rather than full geodynamic reconstructions."

Also, clarify the calibration: how were the erosion rates in Figure S7 chosen and validated against geological proxies?

Thank you for your comment. The erosion rates selected in Supplementary Figure 7 are based on the metamorphic records within the orogenic belt for the corresponding time periods (see Supplementary Figure S06c and Table S06; Zou et al., 2023 EPSL https://doi.org/10.1016/j.epsl.2023.118055)

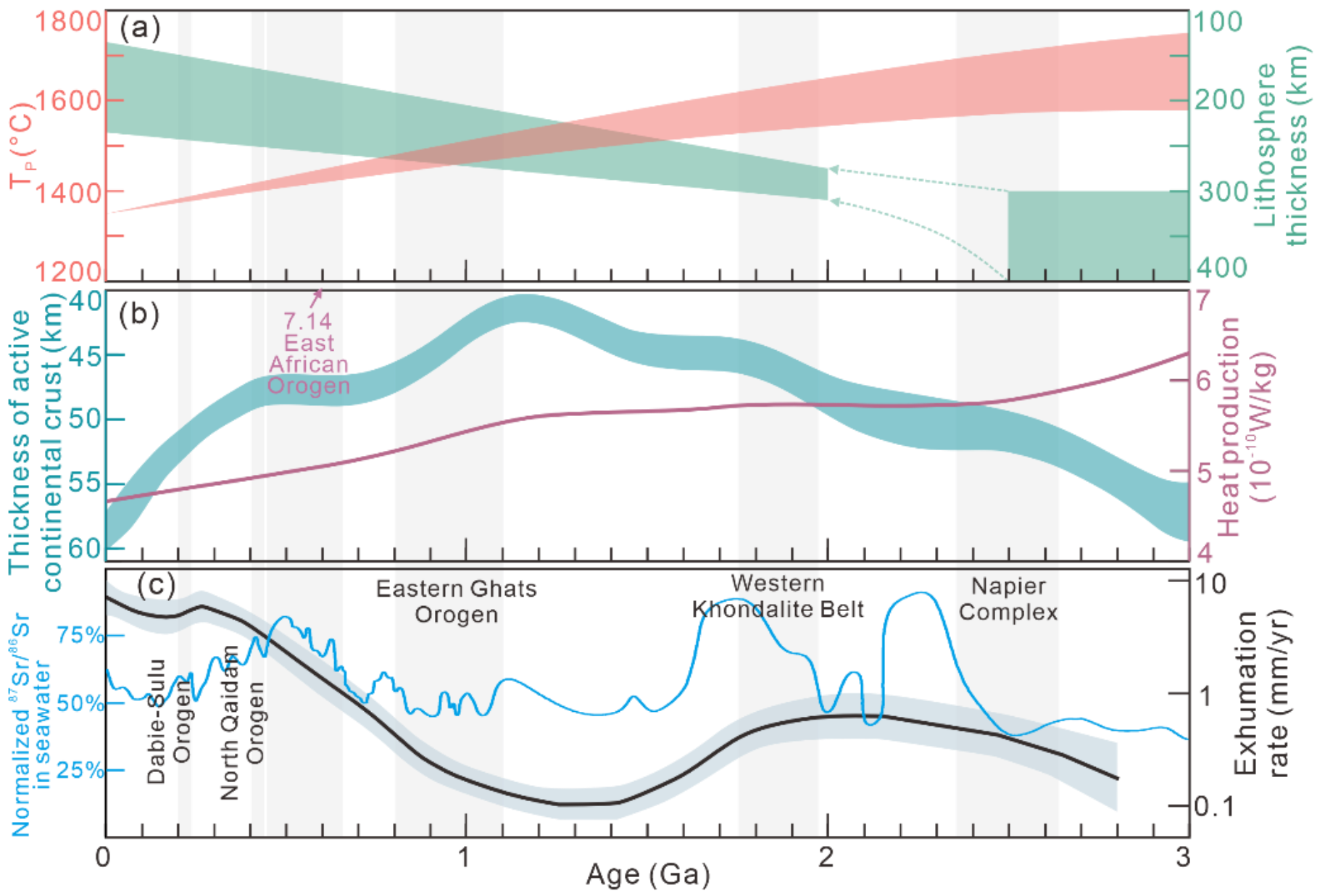

Comment 1.7

(2) Figures 4g–h test mantle-temperature perturbations of 100 °C, yet the authors conclude these are very limited. Quantify the effect magnitude numerically in the caption and discuss sensitivity to radiogenic heat production.

We recognized that our previous description did not adequately clarify the quantitative

impact of variations in mantle temperature and other parameters on the thermal state and orogenic duration, as revealed by the modeling results. Therefore, we have added corresponding explanations in the caption of Figure 4 and discussed the quantitative effects of variations in different parameters on the simulation outcomes (Lines 821–829).

"From Columbia/Nuna (b) to Rodinia (c) assembly, both radiogenic heat productivity and mantle temperature decrease, yet the modeled orogenic duration (increasing from ca. 210 Myr to ca. 300 Myr) and thermal state (UHT duration increasing from ca. 44 Myr to ca. 110 Myr) increase significantly. This indicates that the slow erosional rate during the Rodinia period was the dominant controlling factor. In contrast, with the same radiogenic heat productivity, erosional rate, and orogenic thickness as the Rodinia period, an increase in mantle temperature by 50 °C (g) and 100 °C (h) only prolongs the orogenic and UHT durations by 5 Myr and 8–10 Myr, respectively, indicating a relatively minor effect."

Interpretation and overreach

Comment 1.8

(1) The extrapolation from orogenic duration to plate velocity and plateness in Figure 5b is speculative. The correlation is plausible but not loosely proven. Suggest rephrasing: 'implies enhanced plateness consistent with inferred plate-velocity proxies' rather than asserting direct evidence.

Thank you for your constructive comment. We strongly agree with your perspective. Following your suggestion, we have revised the manuscript accordingly, specifically modifying the wording regarding the extrapolation from orogenic duration to plate velocity and plateness. (Lines 42–44 and 257–260)

(Lines 42–44) "This trend implies enhanced plate rigidity (plateness) and mobility during the Columbia assembly, followed by a marked decline during Rodinia assembly, consistent with inferred plate velocity proxies."

(Lines 257–260) "However, widespread high to moderate dT/dP metamorphism[55], combined with extensive broad deformation domains and prolonged high-thermal states, implies relatively low plateness and surface mobility[2] consistent with inferred plate-velocity proxies[73] (Fig. 5b), revealing a relatively sluggish tectonic regime[10]."

Likewise, the claim of 'proto-modern plate tectonics' during Columbia assembly (see Abstract lines 42–44) should be modified unless independent kinematic or paleomagnetic constraints are cited.

We agree with this perspective. In accordance with your suggestion, we have revised the relevant statement and removed the description of 'proto-modern plate tectonics'. (Lines 42–44)

"This trend implies enhanced plate rigidity (plateness) and mobility during the Columbia assembly, followed by a marked decline during Rodinia assembly, consistent with inferred plate velocity proxies."

Figure modifications:

Comment 1.9

(1) Figure 2: Add clear subpanel lettering (a–c) and enlarge Lu–Hf isochron plots for readability.

Thank you for your careful comment. We have enlarged the subpanel lettering (a–c) in Figure 2 to make them clearer. Regarding the Lu-Hf isochron diagram, the data points are distributed across the entire range of the diagram due to the significant variation in Lu/Hf isotope ratios. While the smaller error ellipses for individual data points reflect lower analytical uncertainties, they do indeed make the data appear less clear. We have now enlarged the overall image to improve its readability.

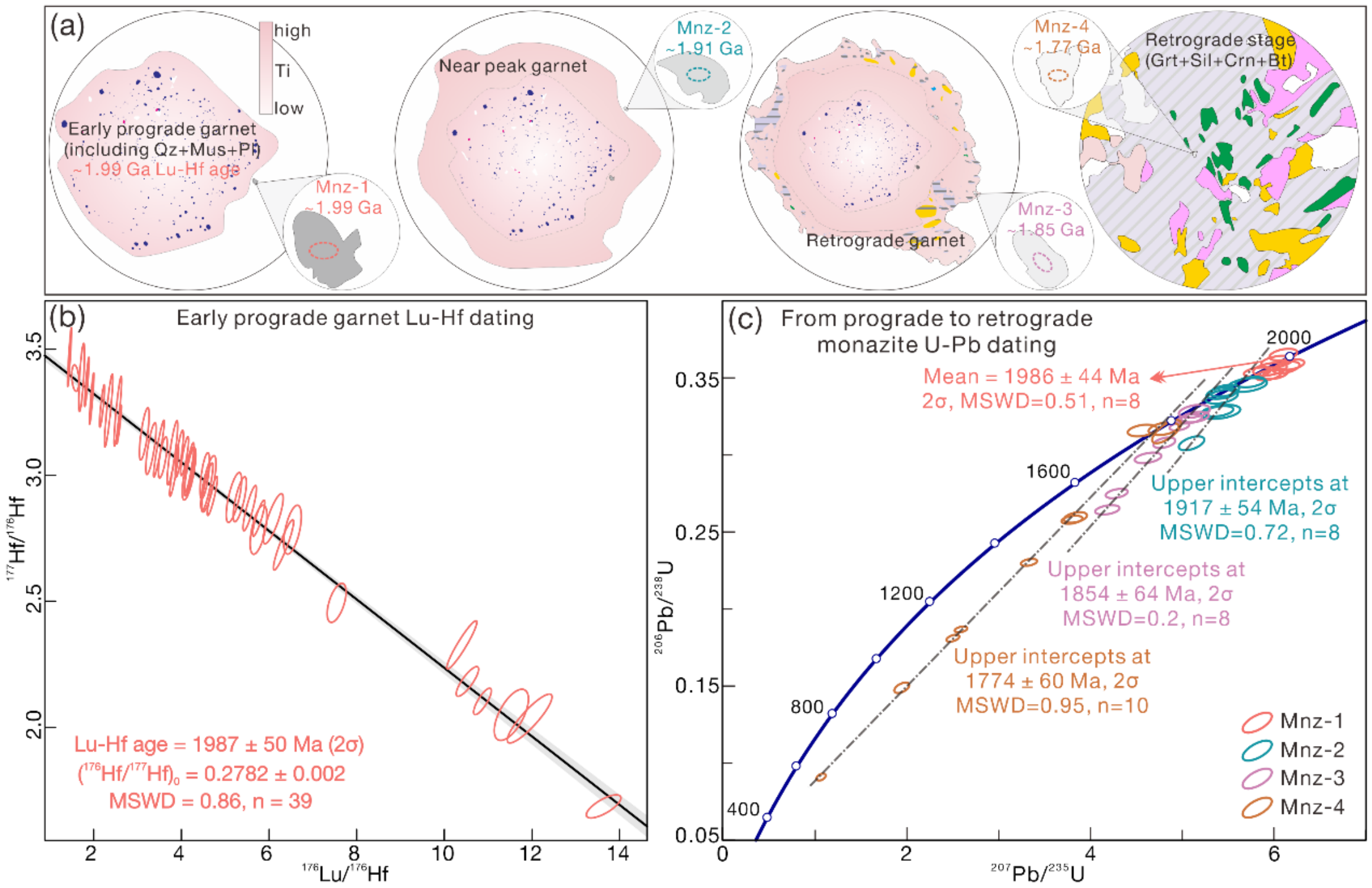

Comment 1.10

(2) Figure 3: Use a uniform temporal scale; mark transitions between Kenor, Columbia, Rodinia, Gondwana, and Phanerozoic clearly on the x-axis.

Thank you for your suggestion. We have revised Figure 3 by adopting a uniform time scale for the x-axis. This adjustment has enhanced the clarity and overall presentation of the figure.

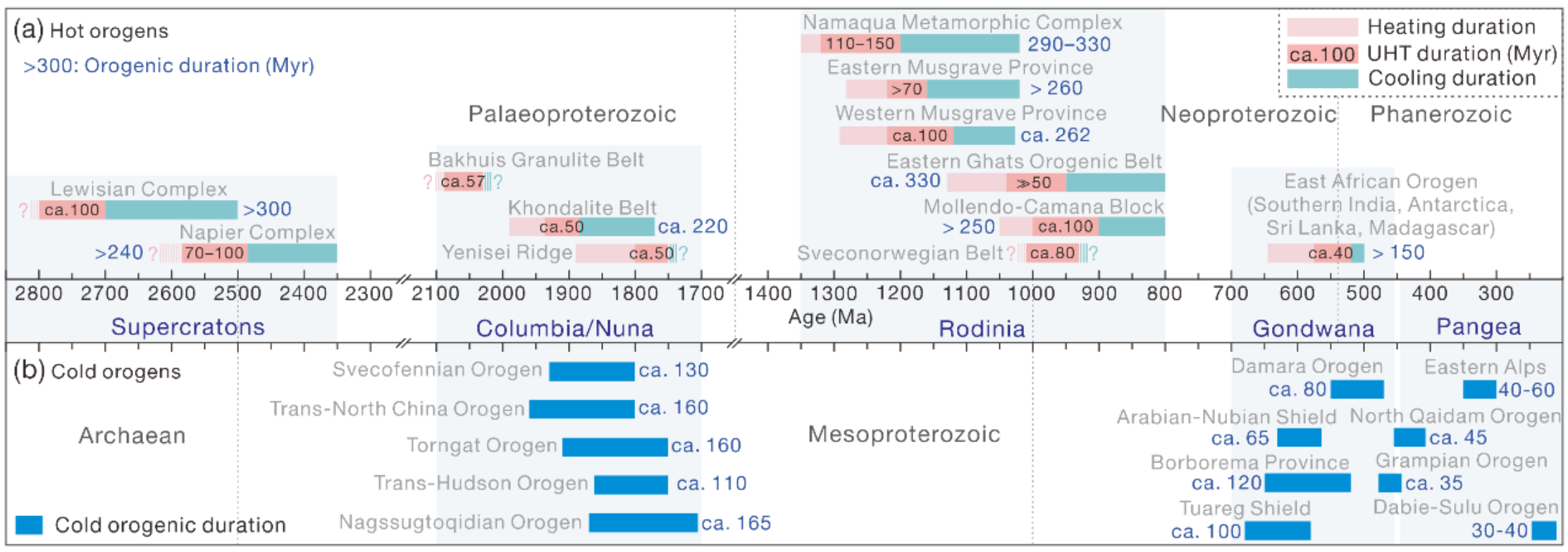

Comment 1.11

(3) Figure 4: Label simulated temperature curves with parameter sets (erosion rate, crustal thickness).

Thank you for your careful comment. As suggested, we have added the corresponding parameters, including the heat production, erosion rate, thickness of orogenic belts, and mantle temperature, to Figure 4.

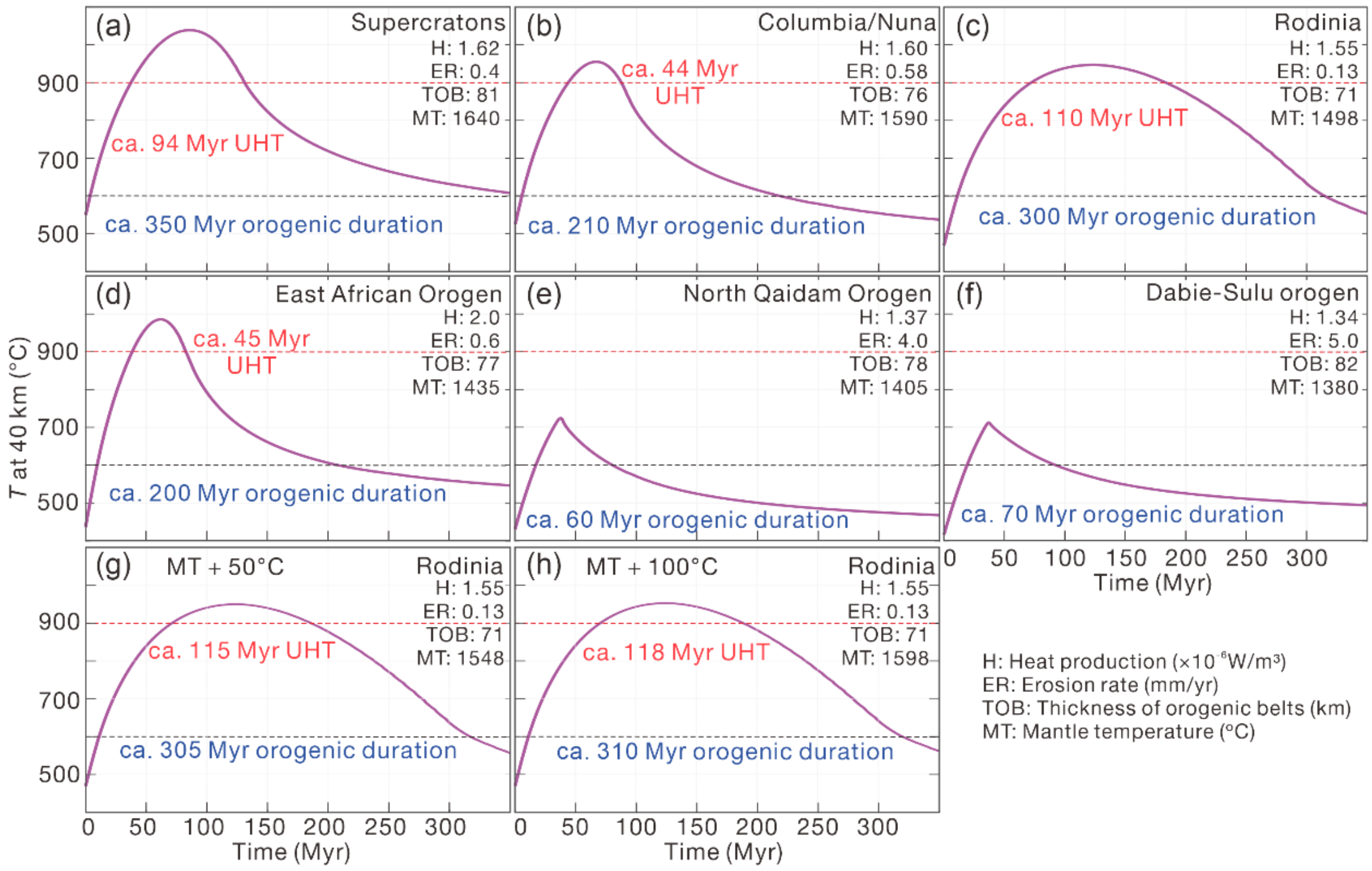

Comment 1.12

(4) Figure 5: Add uncertainty shading and specify data sources for plate velocities (O'Neill 2022).

Thank you very much for your constructive comment. We have revised the figure, presenting the orogenic belt timescale data for each supercontinent assembly period using box-and-whisker plots. We have also added the specified data sources for plate velocities (O'Neill et al., 2022) in Figure 5b.

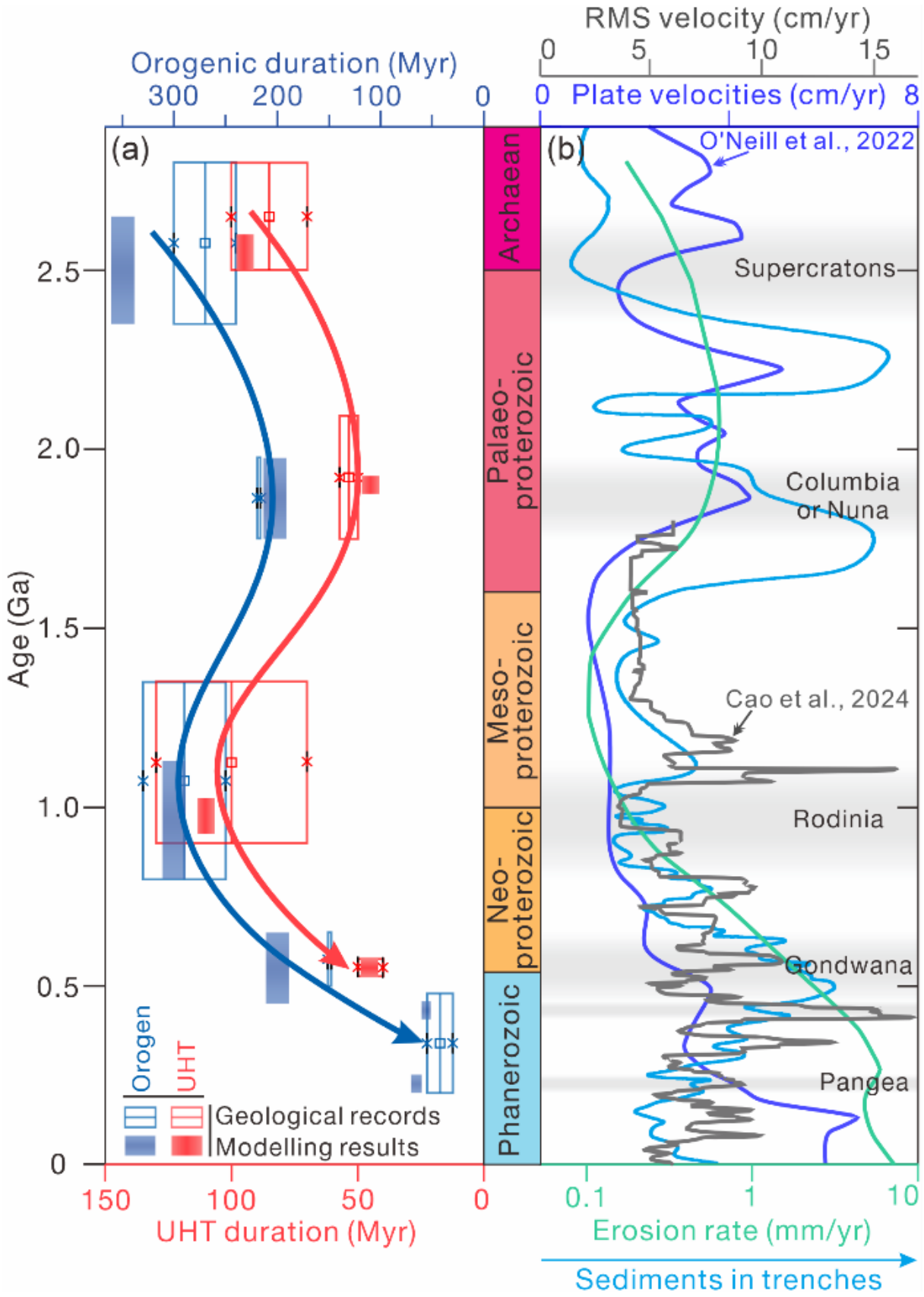

Orogenic duration (Myr)
300
200
100
0
(a)
Age (Ga)
2.5
2.0
1.5
1.0
0.5
0
Orogen
UHT
Geological records
Modelling results
150
100
50
0
UHT duration (Myr)
Archaean
Palaeo-proterozoic
Meso-proterozoic
Neo-proterozoic
Phanerozoic
RMS velocity (cm/yr)
0
5
10
15
0
Plate velocities (cm/yr)
8
(b)
O'Neill et al., 2022
Supercratons
Columbia or Nuna
Cao et al., 2024
Rodinia
Gondwana
Pangea
0.1
1
10
Erosion rate (mm/yr)
Sediments in trenches

Reviewer #2 (Remarks to the Author):

Thanks for the opportunity to read and comment on this interesting manuscript. I congratulate the authors on putting together a good data-focussed paper and then using this as a step to look at the larger Earth system. It is always difficult to justify using a single example as a general case for a wider Earth system and the authors have attempted this well, although not totally convincingly in my opinion. As you will see form the comments below and the annotations on the manuscript, the supplementary figures and the supplementary text attached, I suggest that:

Major comment 2.1

(1) the data can be interpreted just as convincingly (I would suggest more convincingly) as a shorter duration event, or possibly a number of events.

Thank you very much for your valuable comment. The issue you raised regarding the differing interpretations of orogenic duration is indeed pivotal, as it is central to our understanding of the evolutionary characteristics of orogenic belts. We recognize that the discussion of this relevant point lacked clarity in the original manuscript. We have revised the corresponding revisions to address this issue. (Lines 156–163)

"Metamorphic *P*–*T* paths recorded by rocks can reveal the complete evolution of an orogenic belt, including the superposition of multiple tectonic events, and effectively constrain its tectonic and thermal evolution[18,55]. Furthermore, the chronometer minerals within these rocks, such as zircon and monazite, which have relatively fixed closure temperatures (e.g., for the U–Pb system) or growth temperatures, can be used to pinpoint the initiation and termination of orogeny by dating the ages at which the orogenic belt reached those specific temperatures during its heating and cooling history[56,57]."

The detailed reconstruction of metamorphic *P*–*T*–t paths is a primary means by which we invert and decipher orogenic processes, enabling the identification of single or multi-phase metamorphic-tectonic events.

For instance, in the **eastern Khondalite Belt**, petrographic analysis reveals two occurrences of sapphirine within UHT granulites, recording two metamorphic stages (a near-isobaric cooling and a subsequent decompression-heating stage) (Jiao and Guo, 2020 JMG). Combined with chronology and Ti-in-zircon thermometry, Qi et al. (2024) identified two distinct UHT events, including two-stage heating and cooling. This was correlating with the contemporaneous mantle-derived magmatism. We avoided selecting the eastern region for this study because its strong mantle-derived magmatism fails to reflect the normal exhumational history of the orogen.

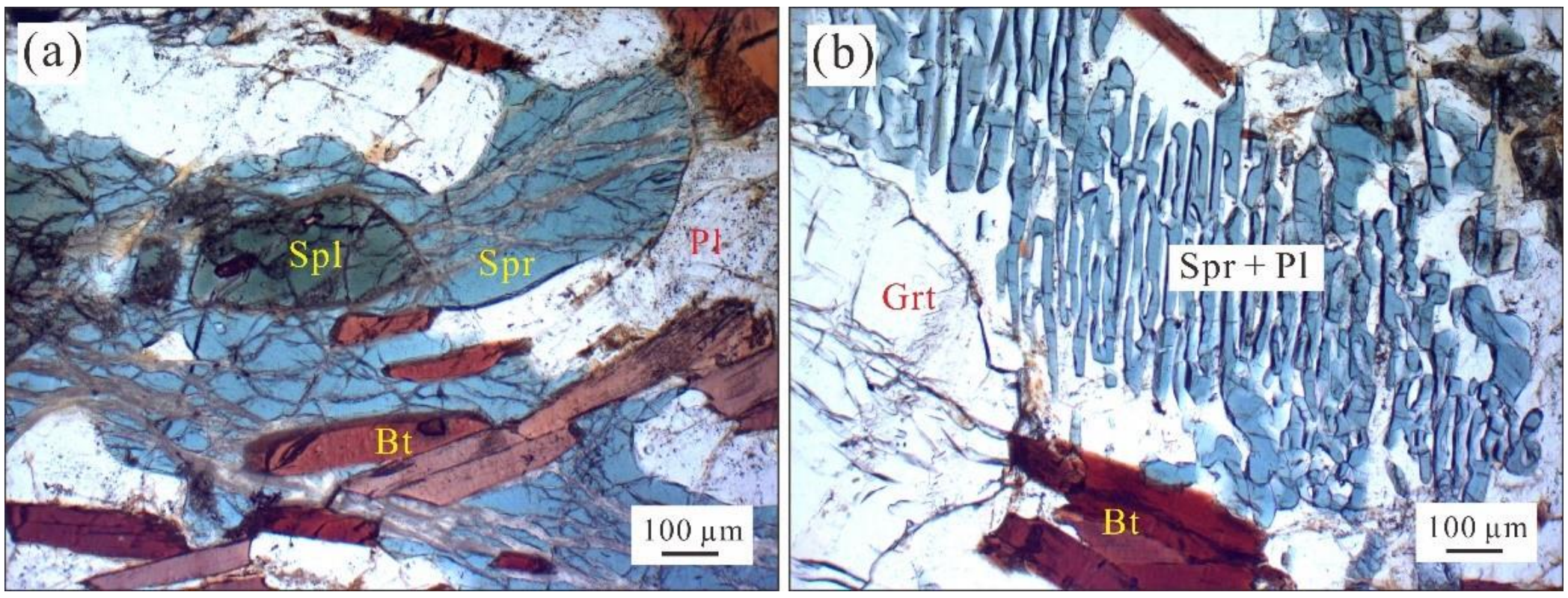

Two occurrences of the sapphirine in the UHT granulite of the eastern Khondalite Belt

(Jiao and Guo, 2020 JMG)

FIGURE REDACTED

The *P*–*T* path (purple, orange, and green arrows) recorded by the sapphirine-bearing UHT granulite of the eastern Khondalite Belt (Jiao and Guo, 2020 JMG), showing two distinct UHT metamorphic events.

In contrast, the UHT granulites from the **western Khondalite Belt** record a simple clockwise *P*–*T* path, with a decompression-heating stage from HP granulite facies to UHT conditions, followed by near-isobaric cooling (Zou et al., 2022, 2024, 2026). They did not record the secondary tectonic uplift observed in the east. Regionally, there is a scarcity of mantle-derived magmatism coeval with UHT metamorphism. Therefore, we conclude that the western Khondalite Belt preserves a more original and complete record of Paleoproterozoic hot orogeny, which is why this study focuses primarily on the western Khondalite Belt.

FIGURE REDACTED

The *P*–*T* path recorded by UHT granulites of the Western Khondalite Belt, showing a single, protracted, hot collisional orogenic process.

In summary, based on detailed metamorphic *P–T* path, we can discriminate between a single, continuous orogeny and multiphase superimposed ones. This capability represents a distinct advantage of using metamorphic petrology to constrain the orogenic events.

Based on your comment, we also recognize that the manuscript lacked a clear explanation regarding the differences between the eastern and western Khondalite Belt and the rationale for our focus on the western part. Accordingly, we have added a more explicit elaboration on this matter in the revised manuscript (Lines 98–107).

"However, the eastern Khondalite Belt was strongly affected by intense mantle-derived magmatism, experiencing multiphase cooling, reheating, and UHT events[40,42]. Consequently, it fails to reflect the typical erosional exhumation processes of a hot orogen. In contrast, the western Khondalite Belt remained largely unaffected by such magmatic activity, recording a simple clockwise temperature–pressure (*P–T*) path, with an early stage involving decompression and heating from HP granulite facies to UHT conditions, followed by near-isobaric cooling[43-46]. These well-preserved records not only provide critical insights into the early prograde evolution of UHT granulites but also offer robust constraints on the duration and thermal state of Paleoproterozoic hot orogenesis."

Jiao, S. J., & Guo, J. H. (2020). Paleoproterozoic UHT metamorphism with isobaric cooling (IBC) followed by decompression—Heating in the Khondalite Belt (North China Craton): New evidence from two sapphirine formation processes. Journal of Metamorphic Geology, 38, 357–378. https://doi.org/10.1111/jmg.12525

Qi, Y. et al. Heating-cooling-heating cycles within ca. 70 Myr recorded in UHT granulites in the Khondalite Belt, North China Craton. Precambrian Research 411 (2024). https://doi.org/10.1016/j.precamres.2024.107508

Zou, L., Guo, J. H., Jiao, S. J., Huang, G. Y., Tian, Z. H., & Liu, P. H. (2022). Paleoproterozoic ultrahigh-temperature metamorphism in the Alxa Block, the Khondalite Belt, North China Craton: Petrology and phase equilibria of quartz-absent corundum-bearing pelitic granulites. Journal of Metamorphic Geology, 40, 1159–1187. https://doi.org/10.1111/jmg.12661

Zou, L., Guo, J.H., Zhang, L.F., Huang, G.Y., Jiao, S.J., Tian, Z.H., Wang, D., and Liu, P.H. (2024), Paleoproterozoic ultrahigh-temperature mafic granulites with a high-pressure prograde path from the Alxa Block: Implications on the tectonic evolution of the Khondalite Belt, North China Craton: Journal of Metamorphic Geology, v. 42, p. 551–581, https://doi .org /10 .1111 /jmg .12764.

Zou, L. et al. (2026). The Paleoproterozoic ultrahigh-temperature metamorphic evolution of Alxa Block, North China Craton: Insights from phase equilibria modeling, garnet trace element chemistry, and monazite geochronology. Geological Society of America Bulletin. https://doi.org/10.1130/b38583.1

Major comment 2.2

(2) The errors need to be quoted at a 2 standard deviation level – by quoting them at 1 standard deviation it makes them look more precise than they are.

Thank you for your comment. We have revised the geochronological data for garnet and monazite throughout the text and figures, updating the reported uncertainties to two standard deviation level. (Lines 123–124, 128, 137–138, and 141; Figures 2, S3, and S5)

Major comment 2.3

(3) I'm afraid that I am not convinced by the age ranges of the orogens that are discussed. These are all quite arbitrary and when looked at in detail become much more complicated

and much more similar to Phanerozoic orogens.

Thank you for your comment. We recognize that the pronounced distinctions between Precambrian and Phanerozoic orogenic belts may not have been clearly articulated in the original manuscript. To address this, we have revised the relevant descriptions to explicitly highlight that early Precambrian orogenic belts are distinctly slower and hotter than their Phanerozoic counterparts (Lines 61–70). These differences are well corroborated by our 1D thermal modeling results presented herein and align closely with global geochronological statistics on orogenic durations.

"Geological records and geodynamic modelling indicate that Precambrian (Neoarchaean–Proterozoic) orogenic belts are distinct from Phanerozoic orogenic belts[9,16]. Specifically, Precambrian belts have slow rates of exhumation constrained by both thermochronology and diffusion chronometry[17], a predominance of high (e.g., ultrahigh-temperature [UHT; T_{max} ≥ 900 °C] granulites) and moderate (e.g., high-pressure [HP] granulites) d*T*/d*P* metamorphism[18,19], and the widespread development of large-scale (> 500 km^2; based on the temperature mapping results) and sustained (UHT duration ≥ 50 Myr) UHT metamorphism[20,21], pointing to a markedly contrasting lithospheric character and tectonic regime compared to Phanerozoic counterparts[8,22-24]."

It is important to note that our consideration of orogenic duration focuses primarily on the thermal evolution—calculated from the onset of crustal thickening and heating to the cooling of the orogen to a specific temperature—rather than solely on tectonic processes. For the hot orogens, the constraint of orogenic duration is challenging, because intense UHT metamorphism typically erases most early prograde records.

This study demonstrates that garnet can record prograde (initial collision) evolution by trace elements and encapsulating early inclusion (e.g., monazite). This allowed us to reconstruct a complete orogenic cycle for a hot orogen. Thus, our constraint on the age of initial collision relies on these sparse age data preserved within mineral inclusions.

To assess the orogenic duration obtained from the thermal ages, we have compiled the mineral isotopic systems and their closure temperatures or growth temperatures for the initial collisional and latest cooling ages in Supplementary Table 5.

Based on these works, the Precambrian hot orogens show significantly longer thermal durations than Phanerozoic orogens. Moreover, we identify a non-monotonic evolutionary trend among Precambrian hot orogens. These results align perfectly with the predictions of our 1D thermal modeling.

Major comment 2.4

(4) Please use more recent reconstructions of supercontinents and I suggest using full-plate models such as Cao et al. 2024 - for consistency and because these are much more constrained than the old continental-drift models.

We have revised the supercontinent reconstruction model and have now referenced the most recent model from Cao et al. (2024). (Figures 1 and S1)

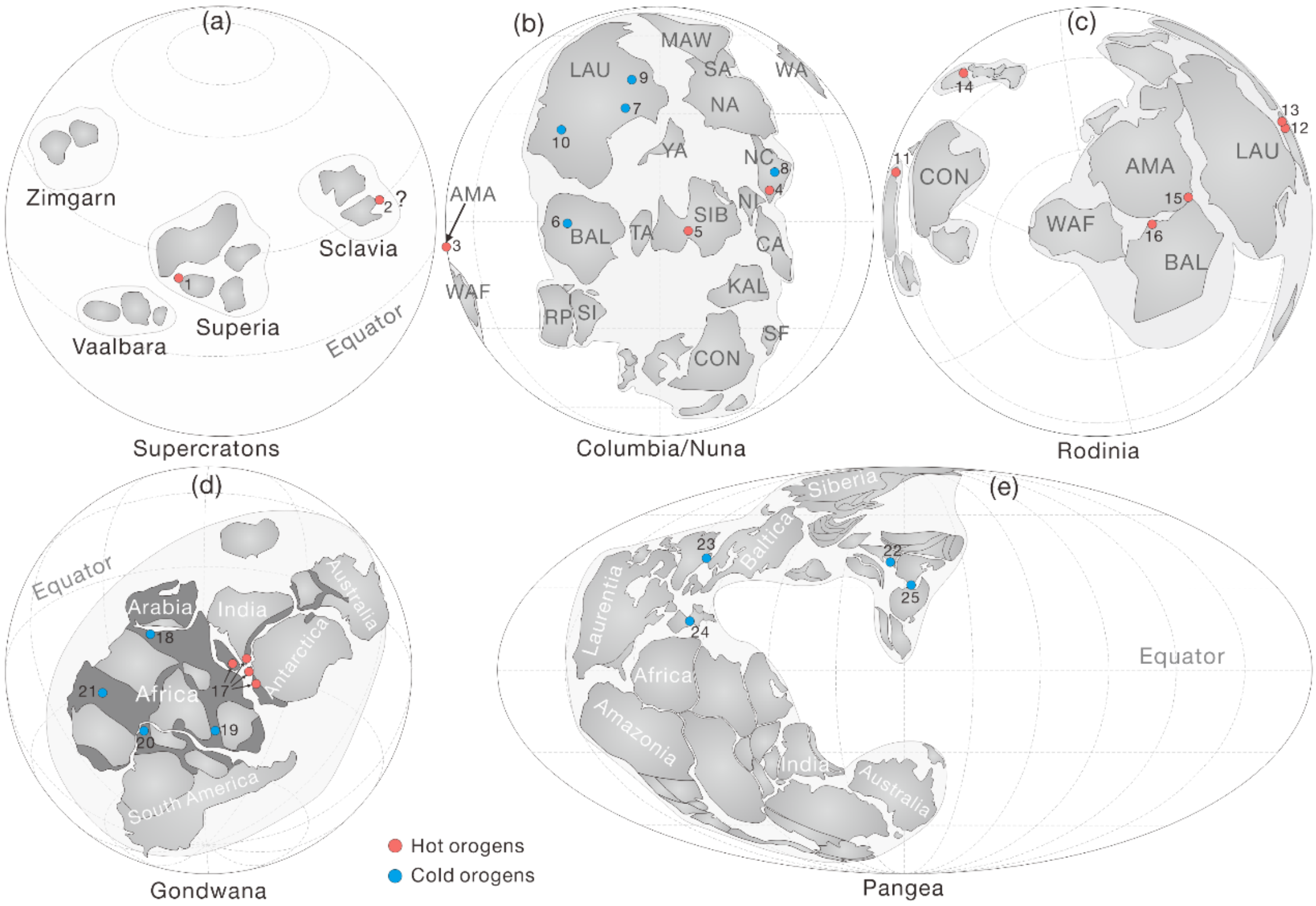

Figure 1

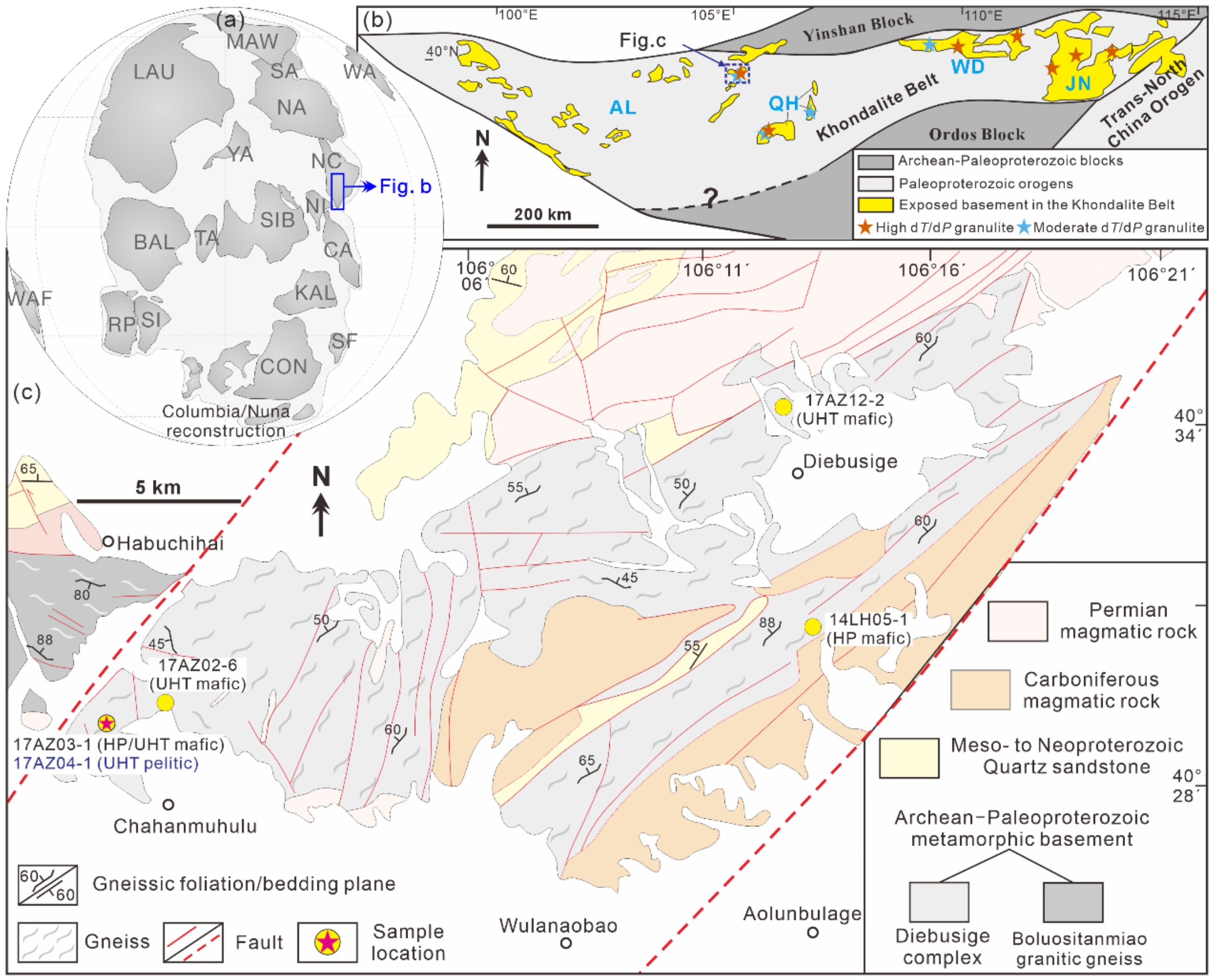

Figure S1

Specific points
Minor comment 2.1

Line 64. *the referenced studies don't really talk about duration of orogenesis - i suggest that this is something that the authors hypothesize here rather than an inferred outcome from previous studies*

Thank you for your careful comment. We searched for and updated the relevant references (Chowdhury and Chakraborty, 2019 JP; Chowdhury et al., 2021 GR) (Line 63). The two new citations integrate evidence from cooling rates in the rock record and results from numerical modeling, demonstrating that early Precambrian orogens had a significantly higher thermal state and experienced slower, more prolonged cooling compared to their Phanerozoic counterparts, reflecting longer orogenic durations.

9 Chowdhury, P., Chakraborty, S. & Gerya, T. V. Time will tell: Secular change in metamorphic timescales and the tectonic implications. Gondwana Research 93, 291-310 (2021). https://doi.org/10.1016/j.gr.2021.02.003

16 Chowdhury, P. & Chakraborty, S. Slow Cooling at Higher Temperatures Recorded within High-P Mafic Granulites from the Southern Granulite Terrain, India: Implications for the Presence and Style of Plate Tectonics near the Archean–Proterozoic Boundary. Journal of Petrology 60, 441-486 (2019). https://doi.org/10.1093/petrology/egz001

Minor comment 2.2

Lines 69-71*. I'm not aware of any regions of this sort of size that show pervasive UHT metamorphism. UHT metamorphism is restricted to small regions where the indicative minerals have been recognised. It is possible that large areas have been effected by these extreme temperatures, but the mineralogy of these regions does not preserve this. Despite the claims in Jiao et al 2023, the Musgraves orogen, the Eastern Ghats Orogen and most of the other <500 km2 orogens they list, do not have demonstrated evidence for UHT metamorphism over the area scale that are quoted - others - e.g. Holder et al for the Anosyen Domain, are associated with extensive magmatism advecting heat into the orogen. I would note that many authors comment on the short duration of UHT metamorphism - e.g. Holder et al.*

Thank you for your comment. We agree with your observation that UHT granulites containing diagnostic mineral assemblages (e.g., sapphirine + quartz) are only found in a limited number of localities within an orogenic belt. However, this does not imply that UHT metamorphism was restricted solely to these sites, as rocks bearing such diagnostic assemblages do not represent all UHT rocks.

Indeed, the diagnostic mineral assemblages of UHT rocks only occur in Mg-Al-rich rocks, and other rock compositions fail to grow (not preserve) diagnostic mineral assemblages even under UHT conditions. Furthermore, such specific Mg-Al-rich compositions are quite rare in nature. In contrast, the new thermometers (e.g., ternary feldspar, Zr-in-rutile/zircon, Ti-in-quartz thermometry) applicable to ubiquitous minerals (feldspar, quartz, zircon, rutile) more effectively identify UHT rocks and enable estimation of UHT metamorphic spatial extent through mapping.

→New text has added details on the mapping methods for identifying the acreages of UHT terranes (Lines 67–68 and 95–96).

For the Anosyen Domain in southern Madagascar, Horton et al. (2016) conducted a temperature mapping using the Zr-in-rutile and Ti-in-quartz thermometers and estimated the UHT area to be more than 20,000 km^2, highlighting the large-scale UHT events. Based on the significantly high concentrations of radioactive elements (locally exceeding 5 $\mu W/m^3$), Horton et al. (2016) demonstrated that the large-scale UHT metamorphism in the Anosyen Domain primarily resulted from radiogenic heating by 1D thermal modeling. A comparable scenario is reported in the Southern Granulite Terrane of southern India within the East African Orogen (Clark et al., 2015).

Certainly, within the regional UHT thermal regime generated by radiogenic heating in the Anosyen Domain, localized contributions from mantle-derived magmatism are also possible, as noted in Holder et al. (2018), which you mentioned. Holder et al. (2018) inferred a short prograde heating duration (<29 Myr) for UHT rocks in the Anosyen Domain, suggesting that mantle-derived (rapid) heat may have also contributed to the UHT metamorphism. However, Holder et al. (2018) also emphasized in their abstract that the high radiogenic heat production of the Anosyen Domain was the primary control on the regional UHT metamorphism.

Horton, F., Hacker, B. R., Kylander-Clark, A. R. C., Holder, R. M., & Jöns, N. (2016). Focused radiogenic heating of middle crust caused ultrahigh temperatures in southern Madagascar. Tectonics, 35, 293–314. https://doi.org/10.1002/2015TC004040

Clark, C., Healy, D., Johnson, T., Collins, A. S., Taylor, R. J., Santosh, M., & Timms, N. E. (2015). Hot orogens and supercontinent amalgamation: A Gondwanan example from southern India. Gondwana Research, 28(4), 1310-1328.

Holder RM, Hacker BR, Horton F, Rakotondrazafy AFM. (2018). Ultrahigh temperature osumilite gneisses in southern Madagascar record combined heat advection and high rates of radiogenic heat production in a longlived high-T orogen. J Metamorph Geol. 36: 855–880. https://doi.org/10.1111/jmg.12316

Minor comment 2.3

Line 84. I think it only fair that you refer to Simpson et al. 2021 here - as they pioneered this technique - https://www.sciencedirect.com/science/article/pii/S0009254121002436

Thank you for your comment. We completely agree with this point; it was an oversight on our part to have missed this key reference on the garnet *in situ* Lu-Hf dating technique. We have now added a citation to this article here. (Line 84)

31 Simpson, A. et al. In-situ Lu-Hf geochronology of garnet, apatite and xenotime by LA ICP MS/MS. Chemical Geology 577 (2021). https://doi.org/10.1016/j.chemgeo.2021.120299

Minor comment 2.4

Line 96. This seems quite an exaggeration, there are occurrences of UHT assemblages within the belt, but it is not clear that the whole belt experienced these temperatures (again, many UHT occurrences are associated with high-temperature plutons).

Thank you for your comment. As noted in the previous response (minor comment 2.2), due to the influence of bulk rock composition, diagnostic mineral assemblages alone are insufficient for comprehensively assessing the spatial extent of UHT metamorphism. In contrast, trace element thermometry mapping of more widespread minerals, such as quartz, zircon, and rutile, provides a more effective means of estimating the distribution of UHT metamorphism.

In the eastern Khondalite Belt, Qi et al. (2022 PR) conducted mapping using Zr-in-rutile thermometry and calculated a UHT area of over 3,000 km^2. Extrapolation to the entire Khondalite Belt estimates an area exceeding 7,000 km^2. We acknowledge that this extrapolation is imprecise. Thus, we have revised this to a more conservative estimate of over 3,000 km^2 and added the details on the mapping methods (Lines 95–96).

"Its > 3000 km^2 (based on the mapping of Zr-in-rutile thermometer) UHT metamorphic terranes……"

We agree with your observation regarding UHT occurrences associated with high-temperature plutons, but it may be locally limited. As noted in our earlier response (Major comment 2.3), Precambrian orogens (with slow exhumation and slow rates, high and moderate d*T*/d*P* metamorphism, and the large-scale and sustained UHT) are hotter and more protracted than Phanerozoic orogens. Such slow and extensive UHT metamorphism is generally considered to be primarily driven by radiogenic heat production, rather than mantle heat, characterised by short-lived and rapid heating (Harley, 2016, 2021). This is supported by our 1D thermal modeling results. However, within these predominantly radiogenically heated orogens, localized overprinting by high-temperature pluton activity may occur, as exemplified by the Anosyen Domain mentioned previously.

Qi, Y. et al. Thermal regime of the lower crust in the eastern Khondalite Belt, North China Craton, constrained by Zr-in-rutile thermometry mapping. Precambrian Research 377 (2022). https://doi.org/10.1016/j.precamres.2022.106720

Harley, S. L. A matter of time: The importance of the duration of UHT metamorphism. Journal of Mineralogical and Petrological Sciences 111, 50-72 (2016). https://doi.org/10.2465/jmps.160128

Harley, S. L. in Encyclopedia of Geology (Second Edition) (eds David Alderton & Scott A. Elias) 522-552 (Academic Press, 2021)

Minor comment 2.5

Line 97. the studies that are referred to (e.g. Qi et al 2024) justify this duration specifically state that there are heating and cooling cycles in that ca. 50 Ma duration - they also compare the orogen directly to the modern Himalaya... suggesting a continuity of orogenic processes, rather than a temporal change as proposed here.

We agree with the point you have noted that Qi et al. (2024) reveal two-stage heating and cooling events. In fact, as mentioned above (major comment 2.1), there is a notable difference between the eastern and western Khondalite Belt in the Paleoproterozoic tectonic evolution. We recognize that the manuscript lacked a clear explanation regarding this difference and the rationale for our focus on the western part. Accordingly, we have added an elaboration in the revised manuscript (Lines 98–107).

However, constraining the precise duration of UHT metamorphism remains highly challenging, as geochronology often cannot definitively bracket it. In this study, we estimated the UHT duration as follows: assuming constant heating and cooling rates, we calculated that the rock reached 900°C at ca. 1.935 Ga during heating from HP granulite

facies (~850°C; ca. 1.95 Ga) to peak stage (~950°C; ca. 1.92 Ga) and at ca. 1.88 Ga during cooling from peak stage to near-solidus conditions (~800°C; ca. 1.8 Ga). Consequently, the inferred UHT duration is ca. 55 Myr.

Qi, Y. et al. Heating-cooling-heating cycles within ca. 70 Myr recorded in UHT granulites in the Khondalite Belt, North China Craton. Precambrian Research 411 (2024). https://doi.org/10.1016/j.precamres.2024.107508

Minor comment 2.6

Line 121. Please don't quote 1 sigma errors - they underestimate the real error. The 2 standard deviation error here is 50 Ma and this should be quoted here.

We have revised the geochronological data for garnet and monazite throughout the text and figures, updating the reported uncertainties to two standard deviation level. (Lines 123–124, 128, 137–138, and 141; Figures 2, S3, and S5)

Minor comment 2.7

Line 124. note that the 2 standard deviation error here is 152 Ma - so the two garnet ages are very much within error of each other and probably shouldn't be separated here

Indeed, as you point out, the Lu-Hf ages obtained from the garnet mantle exhibit significantly large errors, making it difficult to convincingly differentiate between the garnet core and mantle based solely on the geochronological data. Therefore, prior to Lu-Hf dating, we conducted detailed textural and trace element analyses on the garnet microdomains, which formed the basis for their genetic classification (see Supplementary Material).

The garnet core: an early prograde inclusion assemblage of Ms + Qz + Pl; bell-shaped Y+HREE zoning; flat Sc, Cr, Ti, and Zr zoning. This suggests that it grew during an early prograde, subsolidus stage before the partial melting.

The garnet mantle: almost lacking inclusions; a significant decrease in Y+HREE content and a rapid increase in Sc, Cr, Ti, and Zr. This indicates that it formed during the heating stage prior to UHT conditions, associated with biotite peritectic reactions.

Therefore, the distinctly different textural and trace element characteristics of the garnet core and mantle reveal their different genetic stages, which is the primary reason for assigning them to different growth generations.

Regarding the Lu-Hf age of the garnet mantle, the relatively low Lu content (<4 ppm; Fig. S02) resulted in a limited range of $^{177}Hf/^{176}Hf$ vs. $^{176}Lu/^{176}Hf$ ratios for the analysis (Fig. S3a), consequently leading to a larger uncertainty in the isochron age. Nevertheless, in the western Khondalite Belt, the ages of ca. 1.96–1.95 Ga corresponds to the HP granulite-facies stage (Yin et al., 2009, 2011; Zou et al., 2024), which is broadly contemporaneous with the biotite peritectic stage (garnet mantle growth). Thus, we interpret the peritectic garnet mantle (1957 Ma) as potentially corresponding to the HP granulite-facies stage, despite its significant analytical uncertainty.

We have revised the relevant expressions in new text (Lines 127–131).

"The peritectic garnet mantle, with Lu-Hf age of 1957 ± 152 Ma (Fig. S3a; the large error caused by the low Lu contents, which provides key mineral chemical evidence distinguishing the garnet mantle from the core), is interpreted as potentially corresponding to the HP granulite-facies stage (ca. 1.96–1.95 Ga[38,44,47,48]; peak collision) in the western Khondalite Belt."

Yin, C. Q., Zhao, G. C., Guo, J. H., Sun, M., Xia, X. P., Zhou, X. W., & Liu, C. H. (2011). U-Pb and Hf isotopic study of zircons of the Helanshan Complex: Constrains on the evolution of the Khondalite belt in the Western Block of the North China Craton. Lithos, 122, 25–38. https://doi.org/10.1016/j.lithos.2010.11.010

Yin, C. Q., Zhao, G. C., Sun, M., Xia, X. P., Wei, C. J., & Leung, W. H. (2009). LA–ICP–MS U–Pb zircon ages of the Qianlishan Complex: Constrains on the evolution of the Khondalite Belt in the Western Block of the North China Craton. Precambrian Research, 174, 78–94. https://doi.org/10.1016/j.precamres.2009.06.008

Zou, L., Guo, J.H., Zhang, L.F., Huang, G.Y., Jiao, S.J., Tian, Z.H., and Liu, P.H. (2024). Metamorphic evolution of high-pressure and ultrahigh-temperature granulites from the Alxa Block, North China Craton: Implications for the collision and exhumation of Paleoproterozoic orogenic belts: Geological Society of America Bulletin, v. 136, p. 3103–3120, https://doi .org /10 .1130 /B37120 .1.

Minor comment 2.8

Line 130. Fig 2a?

Thank you for pointing this out. The correct reference should be Figure 2a rather than Figure 3a. We have now corrected this. (Line 135)

Minor comment 2.9

Line 131. I would suggest that the high HREE concentrations here suggest that the monazite wasn't growing in competition with garnet... so perhaps the garnet grew later (note the large errors on the garnet ages making this plausible). The garnet core likely grew over these pre-garnet monazites.

Thank you for your comment. We agree with your perspective on the growth relationship between Mnz-1 and the garnet core. We have revised this expression in new text (Lines 134–139).

"First-domain monazites (Mnz-1), texturally occurring as inclusions in porphyroblastic garnet core-mantle transitions (Figs. 2a and S4a–b), exhibit high Y+HREE concentrations (Fig. S3b–c), indicating that their growth may commence slightly earlier than the garnet core. Nevertheless, their weighted mean age (1986 ± 44 Ma and 1977 ± 16 Ma; Figs. 2c and S5b) is consistent within error with the Lu-Hf age from garnet core."

Minor comment 2.10

Line 135. So the chemistry suggest that this may date the growth of the early garnet (and is well within the errors of the Lu-Hf ages)

As mentioned above (minor comment 2.7), we have identified three stages of garnet growth based on the texture and trace element characteristics of garnet microdomains (see Supplementary Material). The garnet core and mantle grew before the UHT stage. For garnet rim, its inclusion assemblage is dominated by retrograde sillimanite + biotite. Moreover, the decreasing Ti zoning indicates that it grew during the retrograde cooling stage. Therefore, the growth of Mnz-2, which is enclosed within the garnet rim, should have occurred between the formation of the garnet mantle and rim.

Importantly, the age (1917 Ma) recorded by Mnz-2, which is similar to the previously reported UHT metamorphic ages (ca. 1.92–1.91 Ga; Zou et al., 2026 GSAB) in the region.

Thus, we interpret the age of Mnz-2 as representing the UHT stage.

The relatively depleted Y+HREE characteristics of Mnz-2 can be explained by monazite growth being nearly synchronous with or later than garnet growth, which is also consistent with our interpretation that monazite formation occurred between the garnet mantle and rim stages.

Zou, L. et al. (2026). The Paleoproterozoic ultrahigh-temperature metamorphic evolution of Alxa Block, North China Craton: Insights from phase equilibria modeling, garnet trace element chemistry, and monazite geochronology. Geological Society of America Bulletin. https://doi.org/10.1130/b38583.1

Minor comment 2.11

Lines 141-143. I'm afraid that i don't see that your data can really justify this - you have early garnet that is probably most precisely dated by the M2 monazites at 1917 +/- 52 Ma (2 s.d.) growing over pre-garnet monazites. Then a later garnet growth at ca. 1800 Ma [not super well constrained as many of the monazites show Pb loss and mixed zones - see fig. S5a here for a nice example].

Thank you for your comment, which has made us aware that our initial description and presentation of this part of the manuscript was inadequate, making it difficult for readers to comprehend. We have revised the text accordingly (Lines 146–152).

"Collectively, combining our multi-chronometer framework and the previous metamorphic (a single UHT event[43,52]) and geochronological data, reveals a collisional orogenic duration of ca. 220 Myr for the western Khondalite Belt during the Palaeoproterozoic. Collision commenced at ca. 1.99–1.98 Ga (this study), progressed to peak collision by ca. 1.96–1.95 Ga[38,44,48,49], culminating in the thermal peak at ca. 1.92–1.91 Ga (e.g., ref.[45,50,53,54] and this study) and concluded with gradual cooling until ca. 1.8–1.77 Ga (e.g., ref.[43-45] and this study)."

Here, by combining our multi-chronometer framework and the previous geochronological data, we reveal a collisional orogenic duration of ca. 220 Myr for the western Khondalite Belt during the Palaeoproterozoic.

In fact, the most crucial aspect of our work on the Khondalite Belt is the identification of the early prograde garnet microdomain (core) and the geochronological constraints on this stage (ca. 1.99–1.98 Ga) provided by garnet Lu-Hf and monazite U-Pb dating.

In addition, the youngest monazite (Mnz-4; 1774 Ma) is texturally associated with retrograde minerals (sillimanite and biotite), representing the latest cooling age recorded by the rock. This is consistent with the record of monazite, occurring in retrograde minerals (biotite), from the spinel-bearing UHT granulite in this region (Zou et al., 2026). Therefore, we conclude that the orogenic cooling record lasted until ca. 1.77 Ga.

Zou, L., Huang, G.Y., Jiao, S.J., Prasanth, M.M., Liu, P.H., and Guo, J.H., 2026. The Paleoproterozoic Ultrahigh-Temperature Metamorphic Evolution of Alxa Block, North China Craton: Insights from Phase Equilibria Modeling, Garnet Trace Element Chemistry, and Monazite Geochronology. Geological Society of America Bulletin, 138: https://doi.org/10.1130/B38583.1

Minor comment 2.12

I don't think that you can say that this was one continuum either - in fact Qi et al. 2024

suggest that there was a cooling and a reheating within this time frame

As mentioned above (Major comment 2.1 and minor comment 2.5), there is a notable difference between the eastern and western Khondalite Belt. Qi et al. (2024) revealed the two-stage heating and cooling process in the eastern Khondalite Belt, which is the result of the significant overprinting of mantle-derived mafic magmatism. In contrast, the western Khondalite Belt lacks the overprinting by mantle-derived magmatism. The *P*–*T*–t paths of UHT granulites record the single, sustained collision-orogeny process (Zou et al., 2024, 2026). We have added an explicit elaboration on this matter in the revised manuscript (Lines 98–107).

Given the significant differences between the eastern and western Khondalite Belt, the distinction in their metamorphic histories is critical. Therefore, we have also added relevant citations to metamorphic data in the text and emphasized that a single UHT event occurred (Lines 146–149).

"Collectively, combining our multi-chronometer framework and the previous metamorphic (a single UHT event[43,52]) and geochronological data, reveals a collisional orogenic duration of ca. 220 Myr for the western Khondalite Belt during the Palaeoproterozoic."

Minor comment 2.13

Lines 151-154. Yes, but, as the study above suggests, these minerals often grow sub-closure temperature - meaning that the ages do not directly reflect the closure temperature.

We fully agree with your view. Accessory minerals used for geochronology, such as zircon and monazite, often grow below their closure temperatures during prograde or retrograde metamorphic stages; consequently, the ages they record do not directly reflect these closure temperatures. This introduces uncertainty when we evaluate the timescales of individual orogenic belts. This point was also raised by Reviewer #1.

Accordingly, we have compiled in Supplementary Table 5 the mineral isotopic systems corresponding to the thermal record ages for each orogen, along with their respective closure temperatures or growth temperatures. By comparing these growth and closure temperatures, we are able to make a comprehensive assessment of the initial temperature conditions and the timescales recorded for each orogenic belt.

Minor comment 2.14

Line 162. you need to define these here

Thanks for your suggestion. We have added the definitions of both orogen types and the corresponding references to the text (Lines 171–172). We define cold orogenic belts based on the appearance of low d*T*/d*P* (~150–375 ºC/GPa; Brown and Johnson, 2018) metamorphic mafic rocks. This occurs because only when the orogenic thermal structure is sufficiently cold can the denser, low d*T*/d*P* subducted metamafic rocks be exhumed to the surface. This process profoundly impacts plateness and mobility.

"(e.g., hot [elevated thermal structures[21,22]] and cold [the appearance of low d*T*/d*P*[18] metamorphic mafic rocks defined in this study])"

Brown, M. & Johnson, T. Secular change in metamorphism and the onset of global plate tectonics. American Mineralogist 103, 181-196 (2018). https://doi.org/10.2138/am-

2018-6166

Minor comment 2.15

Line 168-169. Seeing that the region is extremely poorly exposed, you cannot be sure of this extent at all - even less than for well exposed orogens

Thank you for your careful comment. Given the extremely severe glacier coverage in Antarctica, the estimation of the distribution area of UHT metamorphism may not be rigorous. Therefore, we have removed the specific numerical estimate and revised the related description accordingly (Lines 178–179).

"In the Neoarchaean Napier Complex, UHT metamorphism developed over an extensive area and persisted for 70–100 Myr (from ca. 2.59 to 2.49 Ga; ref.[58,59])……"

Minor comment 2.16

Lines 180-181. I'm not quite sure how this is calculated - there is accretionary orogenesis here lasting from 1080 Ma to 650 Ma when there is a major collision and crustal thickening - then a second collision and crustal thickening at ca. 580 Ma with peak UHT metamorphism at ca. 550 Ma and cooling to ca. 500 degrees by 520 Ma.

Thank you for your constructive comment. Upon careful re-evaluation, we identified that the previously calculated cooling duration for the East African Orogen was overestimated. We are sorry for our careless mistake. The latest ages recorded by a substantial dataset of zircon and monazite are approximately 500 Ma, while the 450 Ma age is based on K-Ar dating. We have revised the data accordingly (Lines 188–192).

"The Neoproterozoic-Cambrian East African Orogen, including Southern India, Antarctica, Sri Lanka, and Madagascar, again documents a significantly shortened collisional cycle of over 150 Myr, including ca. 40–60 Myr heating, ca. 40–50 Myr UHT duration, and over 50 Myr cooling period (Fig. 3d; ref.[62-64])."

We understand your question regarding this point. A comprehensive investigation of the existing literatures reveals that the orogenic process during the Gondwanan period is relatively complex (e.g., Collins and Pisarevsky, 2005 ESR; Cawood and Buchan, 2007 ESR; GAF-BGR, 2008; Collins et al., 2012, 2014 GR; Clark et al., 2015; Horton et al., 2016). As you noted, prior to the collisional orogeny at ca. 580 Ma, there was an arc-continent accretionary process (ca. 650–630 Ma).

Based on the monazite ages of ca. 630 Ma (Jöns and Schenk, 201; Horton et al., 2016), the high concentrations of radioactive elements, and the 1D thermal modeling results, Horton et al. (2016) proposed that the early accretion of the Vohibory arc would have already initiated crustal thickening and prograde metamorphism before the UHT events. As mentioned above (Major comment 2.3), our consideration of orogenic duration focuses primarily on the thermal evolution. Thus, the early crustal thickening (ca. 650–630 Ma) is understood as the onset of thermal evolution (Horton et al., 2016).

Then, the zircon and monazite record the cooling to ~900°C by ca. 530–520 Ma (e.g., Durgalakshmi et al., 2021; Clark et al., 2015), with UHT duration ca. 40–50 Myr.

Finally, the ca. 500 Ma ages recorded by zircon correspond to its closure temperature of ~800°C (Tucker et al., 2014; Durgalakshmi et al., 2021). This temperature is higher than the 600°C threshold used in our 1D thermal modeling to define the orogenic end. This explains why the geological record (ca. 150 Myr duration) appears shorter than the modeled duration (ca. 200 Myr).

On the other hand, the extensive and enduring (ca. 40–50 Myr) UHT in the East African Orogen requires radiogenic heating as the primary heat source rather than mantle heat (characterised by short-lived and rapid) (Harley, 2016, 2021). The ca. 40–60 Myr prograde heating is also consistent with the radiogenic heat source.

Collins, A. S., and S. A. Pisarevsky (2005), Amalgamating eastern Gondwana: The evolution of the Circum-Indian Orogens, Earth Sci. Rev., 71(3–4), 229–270.

Cawood, P. A., & Buchan, C. (2007). Linking accretionary orogenesis with supercontinent assembly. Earth-Science Reviews, 82(3-4), 217-256.

Collins, A. S., P. D. Kinny, and T. Razakamanana (2012), Depositional age, provenance and metamorphic age of metasedimentary rocks from southern Madagascar, Gondwana Res., 21(2), 353–361.

Collins, A. S., C. Clark, and D. Plavsa (2014), Peninsular India in Gondwana: The tectonothermal evolution of the Southern Granulite terrain and its Gondwanan counterparts, Gondwana Res., 25(1), 190–203.

GAF-BGR (2008), Final report: Réalisation des travaux de cartographie géologique de Madagascar, révision approfondie de la cartographie géologique et minière aux échelles 1/100 000 et 1/500 000 zone Sud. République de Madagascar, Ministère de l'Energie et des Mines EM/SG/DG/UCP/PGRM, Geochim. Cosmochim. Acta, 74(5), 1579–1592.

Clark, C., Healy, D., Johnson, T., Collins, A. S., Taylor, R. J., Santosh, M., & Timms, N. E. (2015). Hot orogens and supercontinent amalgamation: A Gondwanan example from southern India. Gondwana Research, 28(4), 1310-1328.

Horton, F., Hacker, B. R., Kylander-Clark, A. R. C., Holder, R. M., & Jöns, N. (2016). Focused radiogenic heating of middle crust caused ultrahigh temperatures in southern Madagascar. Tectonics, 35, 293–314. https://doi.org/10.1002/2015TC004040

Jöns, N., & Schenk, V. (2011). The ultrahigh temperature granulites of southern Madagascar in a polymetamorphic context: Implications for the amalgamation of the Gondwana supercontinent. European Journal of Mineralogy, 23(2), 127–156. https://doi.org/10.1127/0935-1221/2011/0023-2087

Durgalakshmi, Sajeev K, Williams I S, et al. (2021). The timing, duration and conditions of UHT metamorphism in remnants of the former eastern Gondwana. Journal of Petrology, 62(12): egab068.

Tucker, R. D., J. Y. Roig, B.Moine, C. Delor, and S. G. Peters (2014), A geological synthesis of the Precambrian shield in Madagascar, J. Afr. Earth Sci., 94, 9–30.

Harley, S. L. (2016). A matter of time: The importance of the duration of UHT metamorphism. Journal of Mineralogical and Petrological Sciences, 111, 50–72.

Harley, S. L. in Encyclopedia of Geology (Second Edition) (eds David Alderton & Scott A. Elias) 522-552 (Academic Press, 2021)

Minor comment 2.17

Line 196. So looking at the parameters used a major control is the thickness of crust that you take from Tang et al. 2021. Note that this paper excludes S-type granites, which means that continent-continent collisional orogens are excluded from the dataset! i.e. the modern Himalaya would not be visible here.

We agree with your viewpoint. Indeed, the issue you raised is discussed in Supplementary Table 6. However, we recognize that this point was not clearly articulated. Accordingly, we have added the description in Methods (Lines 388–390).

"Parameters such as the lithospheric thickness, crustal thickness (the thickness of active continental crust[66] plus the thickness of average continental crust of 30 km)……"

In our modeling, we used the average crustal thickness (30 km) to represent the

passive continental crust and superimposed it with the crustal thickness data from Tang et al. (2021) representing active continental crust to simulate the corresponding continent–continent collisional orogenic process.

Furthermore, although Tang et al. (2021) exclude S-type granites, that does not mean that collisional orogens are excluded from the data set for calibrating thickness calculations, as such orogens also contain I-type magmatism. Indeed, Tang et al. calibrated their data on the Himalayan collisional orogen. Also, we note that S-types would be excluded from accretionary orogens as the type example of S-type granites occurs in eastern Australia (Chappell and White, 1974), but this does not mean that accretionary orogens are excluded from their data set.

Minor comment 2.18

Line 226. Note that with full-plate tectonic reconstructions you don't see any notable slow down – e.g. Cao et al 2024 – Figure 15. The confusion is that previous studies are based on continental drift models that only consider continents.

We appreciate your comment and have updated the latest plate velocity curve derived from full-plate reconstructions (Cao et al., 2024) (see Figure 5b). However, as the new velocity dataset only extends back to 1.8 Ga, and our discussion requires consideration of the Neoarchean to Paleoproterozoic period, we have retained the previous velocity curve for comparative analysis.

We appreciate that the work by Cao et al. and the earlier work by Merdith et al. (2017) have provided important insights into Proterozoic palaeogeographic reconstructions; they are just models and are only one possible (albeit internally consistent palaeogeographic model. Such reconstructions are a bit like balanced structural cross-sections, which, when they work, are terms viable in recognition that they work but need not be the only solution or the correct solution. This is not to diminish their importance in moving science forward, but a recognition that they should not be taken as absolute. Thus, as you rightly point out, the plate velocity for the period 1.7–1.3 Ga (Cao et al., 2024; Fig. 15) is relatively constant, but available data points used in their reconstruction decrease with increasing age and hence lack the fidelity of younger times.

Furthermore, owing to the scarcity of available paleomagnetic data, full-plate reconstructions for the Neoarchean (ca. 2.5 Ga) are particularly challenging. Inferred plate motion rates based on the passive margin record indicate relatively low velocities during this period. These rates align with the protracted, sluggish, and high-thermal characteristics recorded in orogenic belts, as well as with extensive deformation domains, suggesting the lithospheric coupling parameters of relatively low plateness and mobility in the Neoarchean.

Thus, both datasets are used jointly to discuss plate velocity variations. We have added the following statement in the text (Lines 834–837).

"Plate velocities after 1.8 Ga primarily cite results from the full-plate reconstruction models[79], whereas plate-velocity curves before 1.8 Ga are from the reconstructed models based on passive margin record[73], due to the lack of full-plate reconstruction data. RMS-root mean square."

Merdith, A. S., Collins, A. S., Williams, S. E., Pisarevsky, S., Foden, J. D., Archibald, D. B., ... & Müller, R. D. (2017). A full-plate global reconstruction of the Neoproterozoic. Gondwana Research, 50, 84-134.

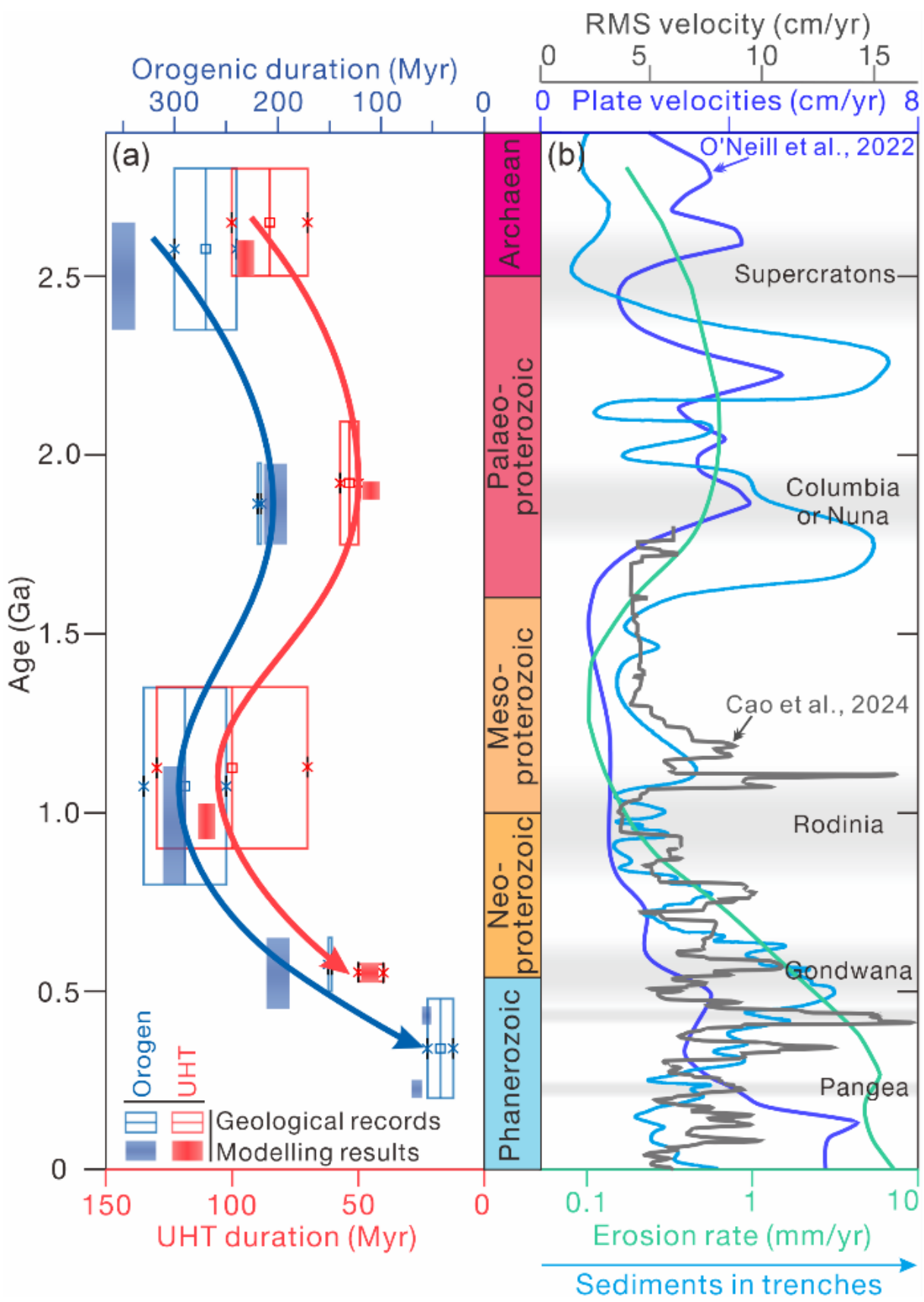

Minor comment 2.19

Line 230-232. these orogens are not anomalously thin though – they have similar geometries to Phanerozoic orogens…

Thank you for your comment. In contrast to the extensive broad deformation domains during the Archean, Paleoproterozoic orogenic belts are characterized by narrow and linear belts, exhibiting a geometry similar to that of Phanerozoic orogens. We have incorporated this description into the text (Line 264).

"…… similar geometries to Phanerozoic orogens……"

Minor comment 2.20

Line 250. This is only when simple continental reconstrcutions are considered - see Cao et al. 2024.

We have updated the latest plate velocity curve derived from full-plate reconstructions (Cao et al., 2024) (see Figure 5b). However, as the new velocity dataset only extends back to 1.8 Ga, and our discussion requires consideration of the Neoarchean to Paleoproterozoic period, we have retained the previous velocity curve for comparative analysis (see also our response to minor comment 2.18). Both datasets are used jointly to discuss plate velocity variations.

For the Mesoproterozoic (ca. 1.6–1.0 Ga), both velocity curves indicate a period of relatively sluggish plate activity, except around ca. 1.1 Ga (Figure 5b). Current interpretations of the significant paleomagnetic variations observed at ca. 1.1 Ga are divided between rapid plate motion and true polar wander. (e.g., Swanson-Hysell et al., 2019; Swanson-Hysell, 2021; Cao et al., 2024). Therefore, combined with the extremely

slow erosion-cooling rates recorded in Mesoproterozoic metamorphic rocks (Zou et al., 2023), we consider that a plate slowdown likely occurred during this period.

Swanson-Hysell, N.L., Ramezani, J., Fairchild, L.M., Rose, I.R., 2019. Failed rifting and fast drifting: Midcontinent Rift development, Laurentia's rapid motion and the driver of Grenvillian orogenesis. GSA Bull. 131 (5–6), 913–940.
Swanson-Hysell, N.L., 2021. The Precambrian paleogeography of Laurentia. In: Pesonen, L.J., Salminen, J., Elming, S-A., Evans, D.A.D., Veikkolainen, T. (Eds.), Ancient Supercontinents and the Paleogeography of Earth. Elsevier, pp. 109–153.
Cao, X. et al., 2024. Earth's tectonic and plate boundary evolution over 1.8 billion years. Geoscience Frontiers. 15. https://doi.org/10.1016/j.gsf.2024.101922
Zou, Y. et al., 2023. Surface evolution during the mid-Proterozoic stalled by mantle warming under Columbia-Rodinia. Earth and Planetary Science Letters. 607. https://doi.org/10.1016/j.epsl.2023.118055

Minor comment 2.21

Lines 258. Rather than 'incomplete' break up - I think more accurate is that the breakup didn't lead to an extraversion - with break up of Laurentia and Australia/NChina/EAntarctica then forming an ocean that closed by introversion... see Cao et al. 2024

Thank you for your careful comment. We have revised the corresponding sentence (Lines 292–295) and supplemented the relevant recent references (Cao et al., 2024 GF).

"The extremely low erosion rates may be linked to the unique breakup mode of the Columbia supercontinent, which limited passive continental margins[82] and maintained largely major continental stability during Rodinia assembly[70,79,81]."

Minor comment 2.22

Line 261-263. The plate velocities that you have in your model are just continent plates, these will not be affected by friction changes at the margins of the supercontinent - as the plates affected are the oceanic plates being subducted. I don;t think that this line is needed here.

We have added the latest plate velocity curve in Figure 5b. As suggested, we have also removed the description in that line (Line 297).

Minor comment 2.23

Fig 1. I strongly suggest using moden full-plate tectonic reconstructions here rather than these older versions - if nothing else you can be consistent for all but the Neoarchaean reconstruction. I suggest using Cao et al. 2024 - Geoscience Frontiers.

We have revised the supercontinent reconstruction model and have now referenced the most recent model from Cao et al. (2024). (Figure 1)

Minor comment 2.24

Fig 2c. This spread of data could also be interpreted simply as ca. 1986 Ma monazite that lost radiogenic Pb at ca. 1800 Ma [or even with mixed analyses of different monazite domains recording these two ages]

We agree that, based on the concordia diagram alone, some data points for Mnz could

potentially be interpreted as the result of Pb-loss or mixing. However, our classification of the four monazite microdomains in this sample is not based solely on the concordia diagram. It is instead constrained by multiple lines of evidence, including BSE imaging, geochemical signatures, and textural positions of the monazite grains.

Firstly, prior to conducting *in situ* analysis on the thin-section scale, we performed detailed geochemical and geochronological analysis on the separated grains (see Supplementary Figure S5). The BSE images, elemental mapping, geochemical data, and age data of monazite exhibit a precise one-to-one correlation. This robust correlation reflects the characteristics of multi-stage growth of distinct monazite microdomains, rather than being a consequence of simple Pb loss or mixing. These defined three microdomains and obtained three ages (1977 Ma, 1912 Ma, and 1841 Ma).

Then, based on the textural analysis between garnet and monazite, we proceeded with *in situ* monazite analysis on the thin-section scale. The results of BSE images, elemental mapping, geochemical data, and age data are similar to the separated-grain monazite. Combined with the textural characteristic, four monazites can be defined and obtained four ages (1986 Ma, 1917 Ma, 1854 Ma, 1774 Ma).

Therefore, we believe that, given the detailed analytical framework outlined above, the different monazite microdomains likely represent distinct growth stages. Furthermore, the shielding effect of garnet has likely trapped and preserved the geochronological information corresponding to these individual growth stages.

Minor comment 2.25

These errors are all quoted at the 1 sigma level and therefore are artificially reduced. They should be quoted at 2 standard deviations (or standard errors) to be realistic of the true error

Thank you for your comment. We have revised the geochronological data for garnet and monazite throughout the text and figures, updating the reported uncertainties to a two-standard-deviation level. (Lines 123–124, 128, 137–138, and 141; Figures 2, S3, and S5)

Minor comment 2.26

why do we not see the 'young' garnet here as well??

Thank you for your careful comment. The Lu-Hf isochron age diagram for the garnet mantle was placed in Supplementary Figure S3. As noted in the comment above (minor comment 2.7), the low Lu content in the garnet mantle resulted in a limited range of measured Lu/Hf isotope ratios, which in turn led to a relatively large uncertainty in the isochron age, making the data less than ideal. Our interpretation of the genesis and metamorphic stage of the garnet mantle is primarily based on its textural and trace element characteristics. Therefore, this is the reason we have placed the corresponding age diagram in the supplementary materials.

Minor comment 2.27

Fig. 3. I'm afraid that I would argue that most of these time lines are gross simplifications - sorry... For example, the Eastern Ghats. There is very little evidence for pre-950 Ma UHT metamorphism (a few monazites in inclusions) and extremely little evidence for the so-

called heating stage.

You are right to point out that pre-UHT geochronological records are scarce for most hot orogens (e.g., the Eastern Ghats), which is fundamentally governed by the behavior of accessory minerals during metamorphism.

Thermodynamic modeling results (Yakymchuk et al., 2017) show that under supersolidus conditions, continued temperature increase leads to pronounced dissolution of monazite in the rock system. With increasing temperature, the dissolution proportion of monazite rises until it is completely exhausted under UHT conditions (see image below). It is only during the subsequent retrograde cooling stage that monazite can recrystallize and grow anew.

FIGURE REDACTED

Image from Yakymchuk et al. (2017 RMG: Phase Relations, Reaction Sequences and Petrochronology)

Therefore, in theory, for UHT rocks, the dominant monazite population typically crystallizes during the cooling stage, recording retrograde ages. This explains why, in UHT granulites from hot orogens, we primarily obtain post-UHT cooling ages.

Fortunately, garnet porphyroblasts can act as protective hosts for some prograde monazite. As mentioned above (Major comment 2.3), our study demonstrates that garnet can preserve prograde ages by encapsulating early monazite inclusion. This allowed us to reconstruct a complete orogenic cycle for a hot orogen. Thus, our constraint on the age of initial collision relies on these sparse age data preserved within mineral inclusions.

Minor comment 2.28

Also, the East African orogen, at 650 Ma there was an earlier continental-collisional orogeny (the East African Orogeny) - there was basin formation (extension?) between that and the subsequent collision at ca. 580 Ma (the Malagasy Orogeny) that caused UHT metamorphism at ca. 550 Ma.

Thank you for your comment. As mentioned above (major comment 2.3 and minor comment 2.16), our consideration of orogenic duration focuses primarily on the thermal evolution. Based on the monazite ages of ca. 630 Ma (Jöns and Schenk, 201; Horton et

al., 2016), the high concentrations of radioactive elements, and the 1D thermal modeling results, Horton et al. (2016) proposed that the early accretion (from 650–630 Ma to 580 Ma) of the Vohibory arc would have already initiated crustal thickening and prograde metamorphism before the UHT events, as the onset of thermal evolution.

Therefore, from the standpoint of metamorphic and thermal evolution, we incorporate this early prograde heating stage (from 650–630 Ma to 580 Ma) into the evolution of the UHT terrane, even though the tectonic contexts of the two stages may differ.

Horton, F., Hacker, B. R., Kylander-Clark, A. R. C., Holder, R. M., & Jöns, N. (2016). Focused radiogenic heating of middle crust caused ultrahigh temperatures in southern Madagascar. Tectonics, 35, 293–314. https://doi.org/10.1002/2015TC004040

Minor comment 2.29

Fig. 5. Note that Cao et al. 2024 have an updated plate velocity curve based on a full earth full-plate tectonic model - this model you have here is based on continent velocities only.

We have updated the latest plate velocity curve derived from full-plate reconstructions (Cao et al., 2024) (see Figure 5b).

Minor comment 2.30

Figure S1. Please use a more recent reconstruction - this one is very out of date - suggest Cao et al. 2024 - GSF

We have revised the supercontinent reconstruction model and have now referenced the most recent model from Cao et al. (2024). (Figures 1 and S1)

Minor comment 2.31

Fig S03. I'm not sure what has happened to Mnz-4 here - firstly, the colours don't seem to match the legend (brown versus orange). Secondly, you seem to suggest that the Mnz-4 here has the largest Eu anomaly, yet in figure (f) below, Mnz-4 has no Eu anomaly??? While Mnz-1 has the largest anomaly - that is NOT what this shows here....
Figure S3 captions
note that the Eu anomaly seems very stange when you compare (b) with (f)???

We are sorry for our careless mistake. We have unified the colours for Mnz-4 and the legend in Figure S03.

Regarding the Eu anomaly, the previous discrepancy in observations was likely due to this error in the monazite label colors in the figure. The issue has been corrected in the revised figure, which should now be accurate.

Eu/Eu* > 1: positive Eu anomaly; Eu/Eu* = 1: no Eu anomaly; Eu/Eu* < 1: negative Eu anomaly. In Fig. S03, the Eu/Eu* values of all monazites are less than 1, indicating negative Eu anomalies for all. Moreover, **the smaller the Eu/Eu* value, the more pronounced (large) the negative Eu anomaly**. Mnz-4 has the smallest Eu/Eu* value (Fig S03f), thus exhibiting the largest negative Eu anomaly, which is consistent with the REE pattern in Fig S03b.

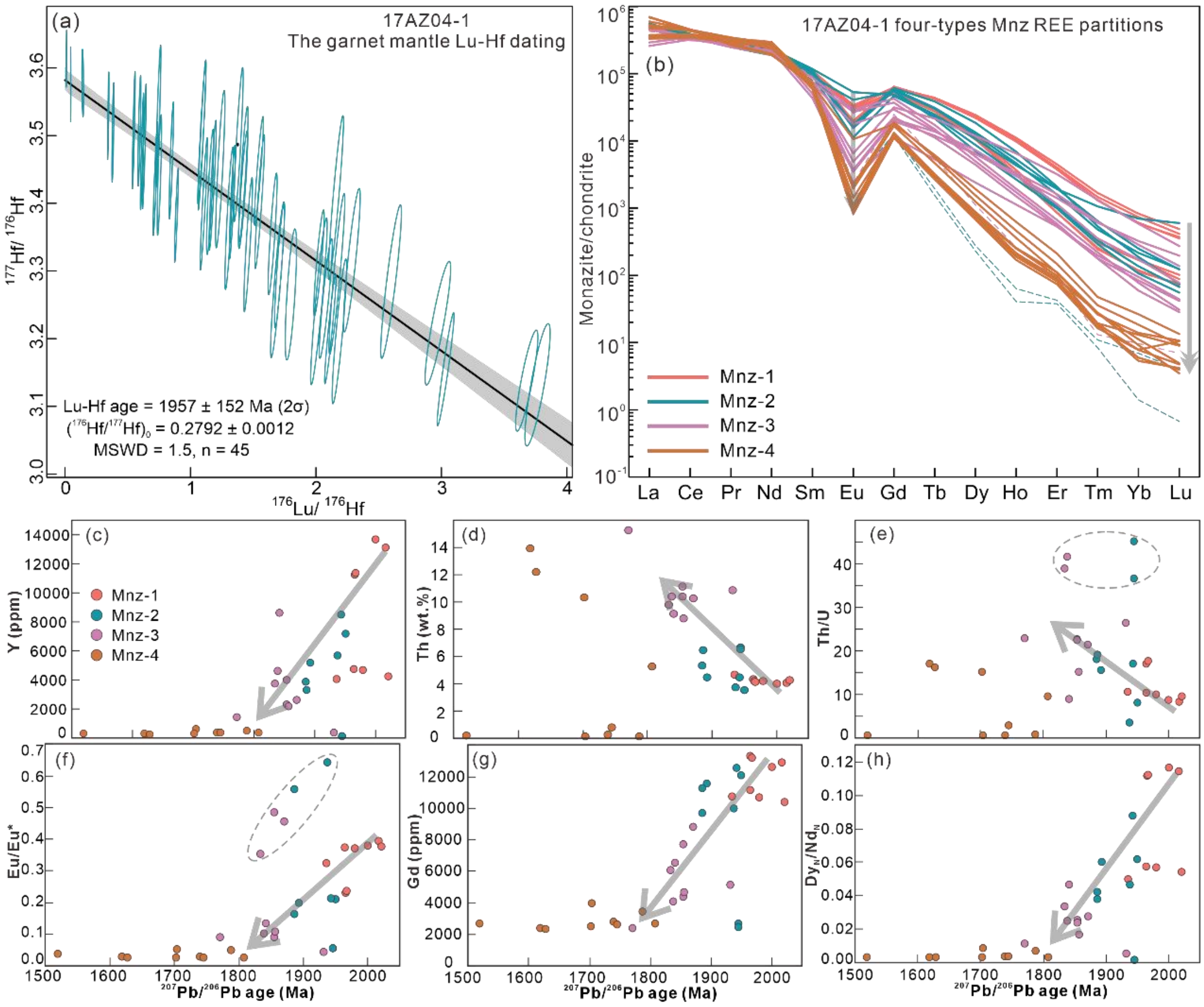

Fig. S03

Minor comment 2.32

Fig S04. It is interesting how this monazite has itself a core and rim even though it is contained as an inclusion in the garnet mantle....

Thank you for your careful and insightful comment. The monazite inclusion located within the garnet mantle, exhibiting a core-rim structure, is particularly interesting. Our interpretation is as follows:

The texture and trace elements of garnet in this study reveal a multi-stage growth history, including an early prograde stage (core), the biotite peritectic stage (mantle), and the retrograde cooling stage (rim). Accordingly, monazite crystals formed at different times may have been encapsulated and shielded by garnet, thereby preserving their respective geochronological information.

However, the episodic growth of garnet was accompanied by intermittent consumption. Phase equilibrium modeling indicates that the garnet mantle in this sample likely formed during a HP granulite-facies metamorphic stage (see image below). After garnet mantle growth, the modal proportion of garnet is predicted to decrease, meaning the garnet mantle was likely consumed. During this process, monazite inclusions previously enclosed within the garnet mantle could have been partially exhumed and consequently overprinted.

Subsequent growth of the garnet rim may have re-entrapped these monazite grains. The rim observed on the monazite inclusion within the garnet mantle may thus represent metamorphic overgrowth that occurred during this exhumation period.

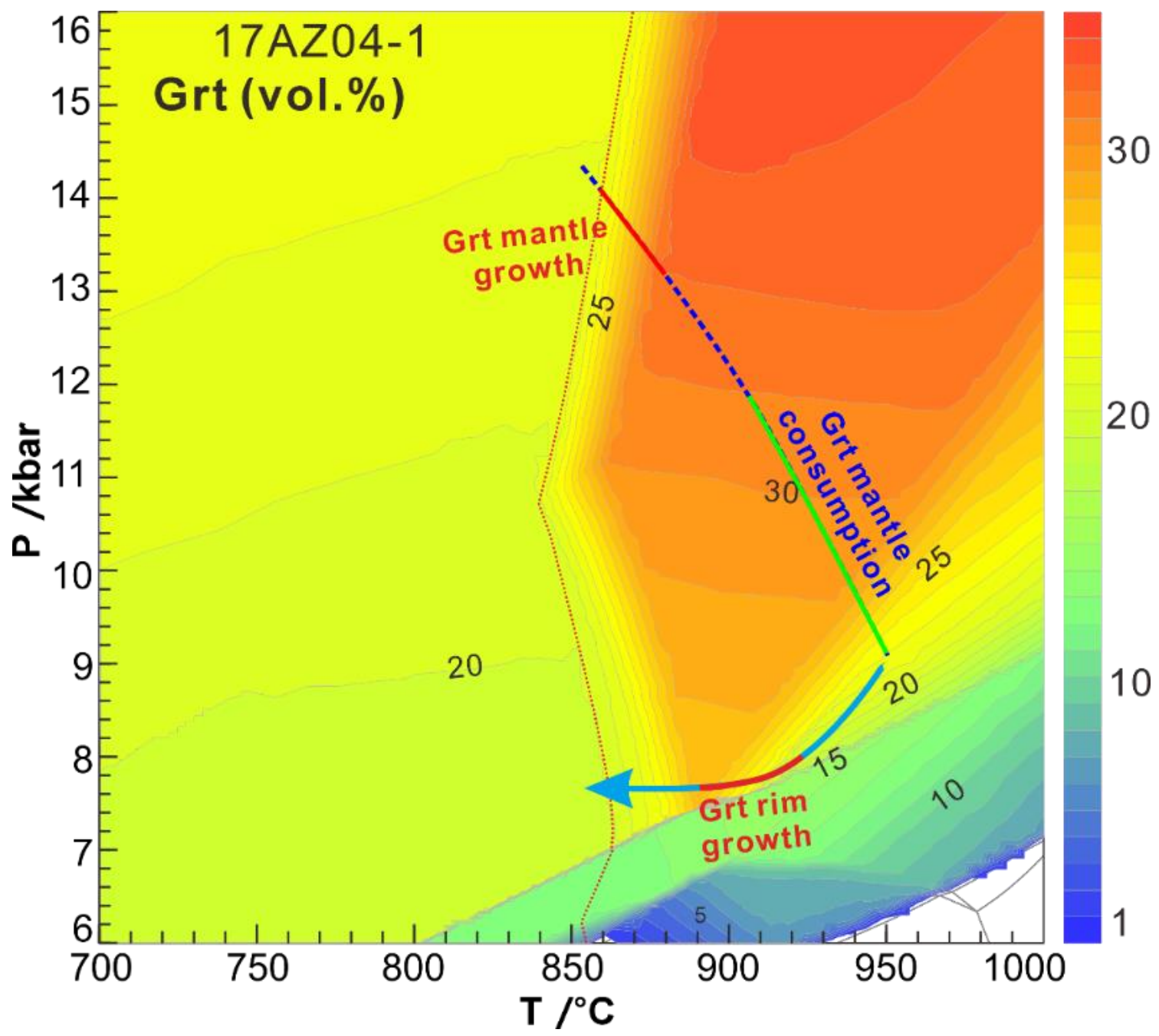

Predicted evolution of garnet volume isopleths from phase equilibrium modeling.

Minor comment 2.33

Also, you can see the 'ghost' of the original extent of the early monazite in the Y and Th maps - suggesting that you have mixed ages in your data.

You are correct in noting that the element mapping and our spot selection protocol indicate the possibility of minor mixing of signals within the analyzed monazite microdomains. We acknowledge that analyzing some of the Mnz-2 microdomains using the thin-section scale *in situ* approach is challenging, as some of these grains are located within very small rim domains. The scarcity of analysis points reflects the inherently limited record of this specific stage in this particular corundum-bearing granulite sample (17AZ04-1).

In contrast, a spinel-bearing UHT granulite sample (21AZ04-2) collected from the same outcrop scale (see image below) records a more abundant age signal for this stage (Zou et al., 2016). In that sample, **the vast majority of garnet grains grew during the retrograde cooling stage (exhibiting trace element characteristics similar to the garnet rims in this study)**. The monazite inclusions within those garnets consistently yield a near-peak UHT age of 1916 Ma, which is consistent with the ages obtained for Mnz-2 in this study (1912 Ma and 1917 Ma).

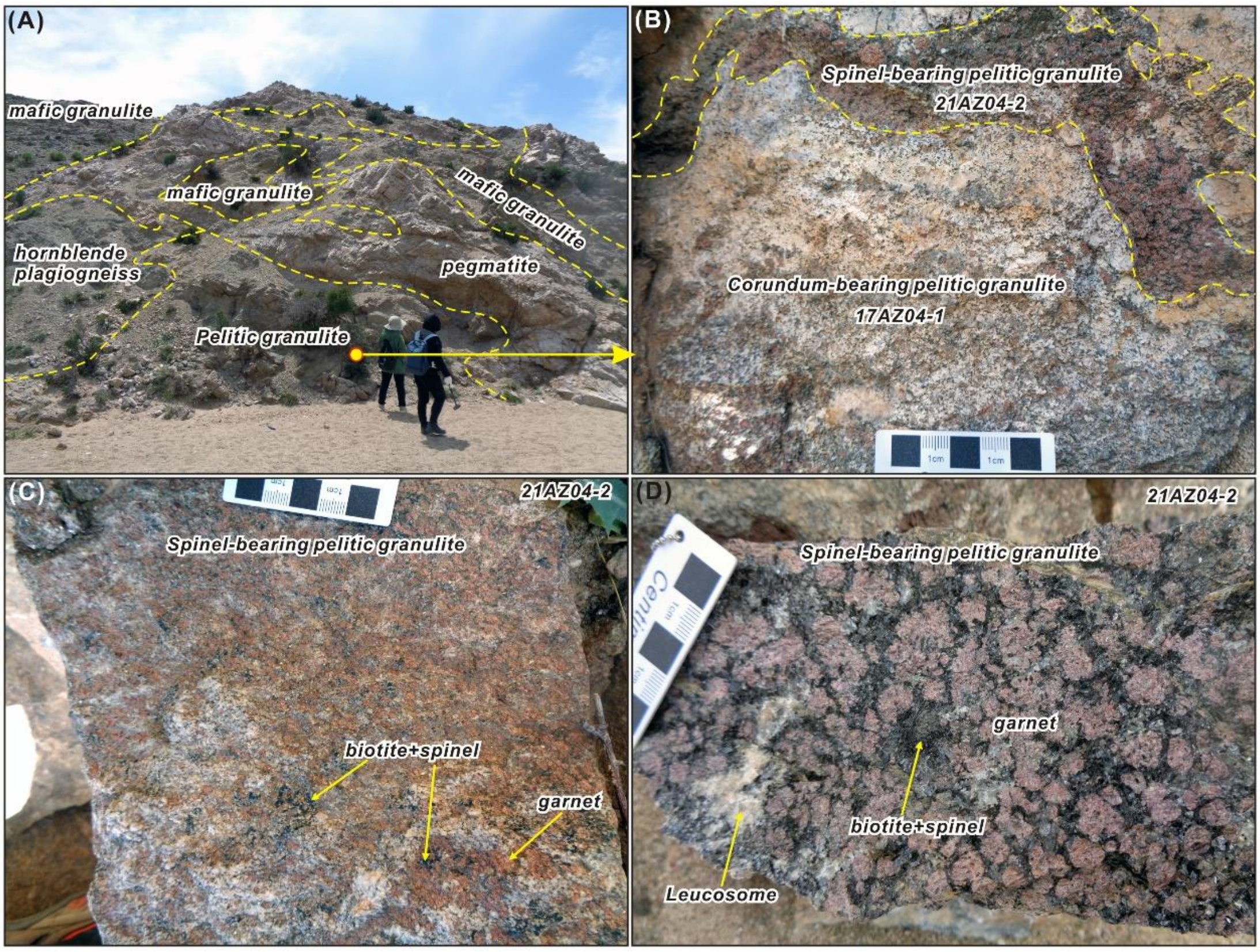

Field contact relationship between corundum-bearing and spinel-bearing metapelitic granulites

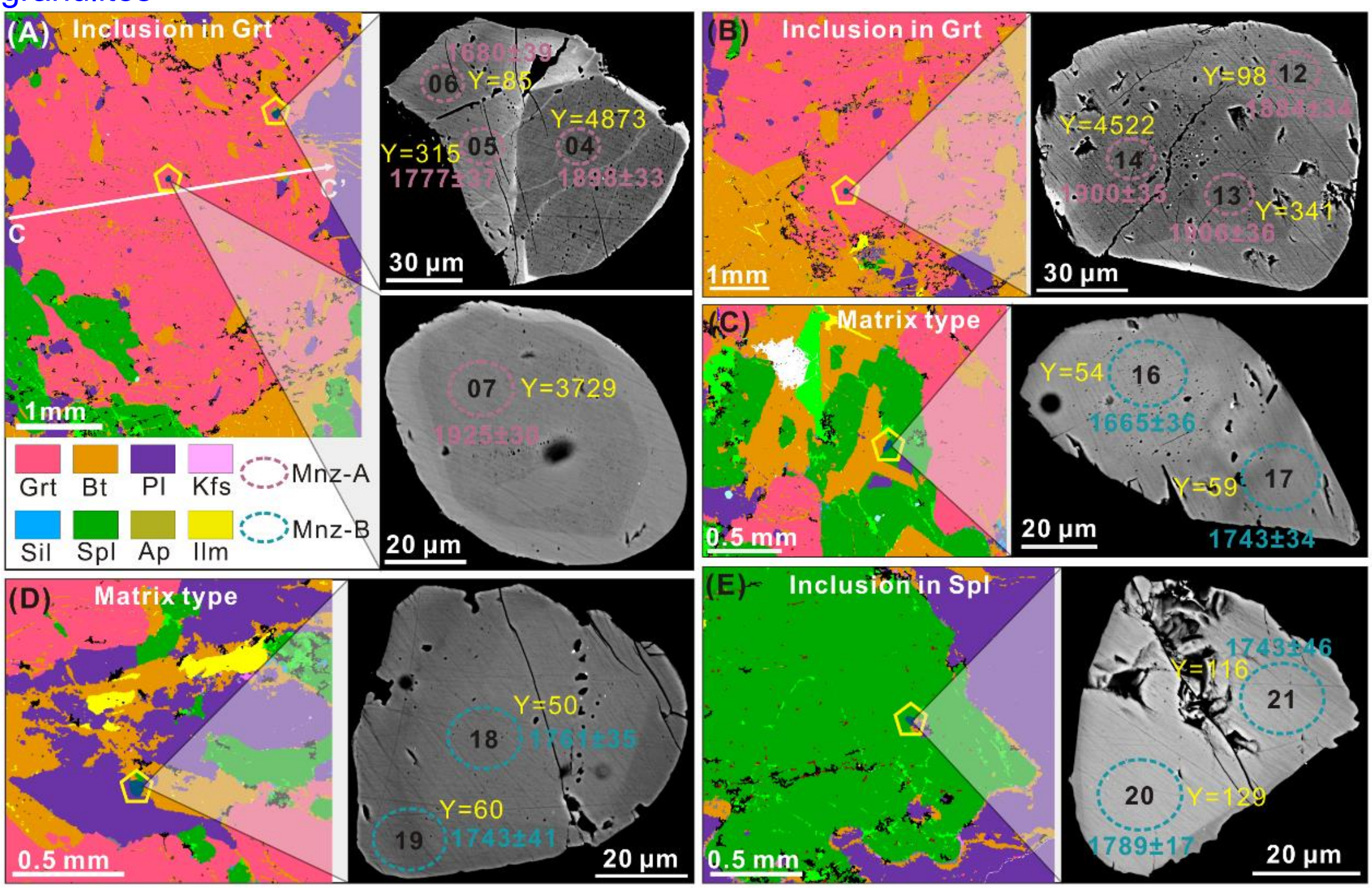

The internal structures of monazite inclusion in garnet and contact with biotite + spinel + plagioclase in matrix from the spinel-bearing UHT granulite sample 21AZ04-2

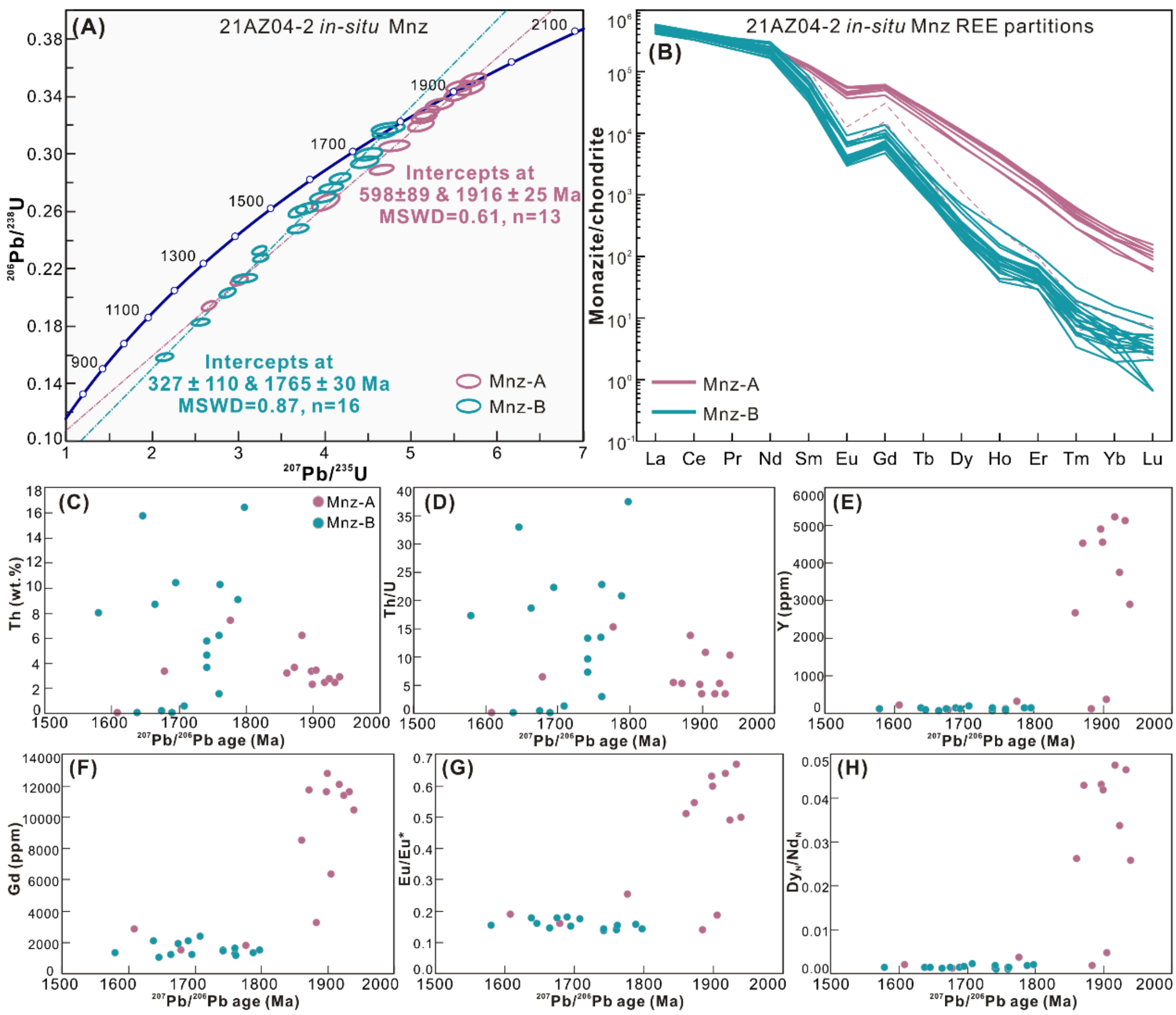

Chronological and compositional characteristics of two texturally controlled types of monazite microdomains from the spinel-bearing UHT granulite sample 21AZ04-2

Therefore, we maintain that the Mnz-2 microdomains in this study indeed represent the corresponding near-peak UHT metamorphic stage. We believe the potential minor mixing in a limited number of analytical spots has a weak impact on the overall geochronological interpretation.

Zou, L., Huang, G.Y., Jiao, S.J., Prasanth, M.M., Liu, P.H., and Guo, J.H., 2026. The Paleoproterozoic Ultrahigh-Temperature Metamorphic Evolution of Alxa Block, North China Craton: Insights from Phase Equilibria Modeling, Garnet Trace Element Chemistry, and Monazite Geochronology. Geological Society of America Bulletin, 138: https://doi.org/10.1130/B38583.1

Minor comment 2.34

Fig S05a. I think another interpretation of these images and data are that this reflects an early monazite age of ca. 1930 Ma that is then overprinted by a later monazite of ca. 1830 Ma with a mixed region between the two - this looks pretty clear with the Pb map in particular.... In fact in the Pb, Th and Ce maps, it looks like you can see the early monazite 'ghost' in preserved even below analysis spot 44 - suggesting that the 'real' later age is probably younger than 1830 Ma... and represented by the deep red (Th) and blue (Ce maps)

Thank you for your careful observation and suggestions. We acknowledge that the

ages of monazite in this sample are quite complex, and some microdomains (e.g., Mnz-2) may allow for multiple interpretations based on the concordia diagram alone. As mentioned above (minor comment 2.24), based on the detailed analysis of monazite microdomains geochemistry, geochronology, and texture (not only for ages), we divided them into four types with different genesis. They represent distinct growth stages, rather than being a consequence of simple Pb loss or mixing.

On the other hand, the primary objective and central theme of this article is to constrain the duration of the orogeny. In this context, the early prograde age (ca. 1.99 Ga) jointly constrained by the oldest monazite (Mnz-1) and the garnet Lu-Hf age is more significant, and it is an age previously unreported for the Khondalite Belt. Regarding the UHT-stage age (ca. 1.92 Ga) and the ca. 1.85 Ga cooling age, there are already abundant reports from other UHT rocks in the western and eastern Khondalite Belt (e.g., Peng et al., 2014; Gou et al., 2019; Jiao et al., 2020; Zou et al., 2026). In this study, the ca. 1.92 Ga age from Mnz-2 is consistent with the previous results from the western Khondalite Belt, so we interpret it as the near-UHT age.

In addition, we fully agree with the issue you raised regarding analysis point 44 from Mnz-3. As mentioned above (minor comment 2.24), we applied two different analytical approaches (separated grains and *in situ* thin-section scale) to the monazite in the sample. The results in Fig. S5 are from the separated-grain monazite. The Mnz-3 BSE image from this approach is overly bright (Fig. S5a), and its chemical microdomain is too narrow (as you noted for Th and Ce). These prevented us from making any further detailed divisions of it. In contrast, the *in-situ* analysis at the thin section found that these Mnz-3 have two textures (garnet outermost rim and included in retrograde minerals). The latter records the 'real' later age of ca. 1774 Ma (Fig. 2c).

Peng, P., Wang, X., Windley, B.F., Guo, J., Zhai, M., and Li, Y., 2014. Spatial Distribution of ~1950–1800 Ma Metamorphic Events in the North China Craton: Implications for Tectonic Subdivision of the Craton. Lithos, 202–203: 250–266. https://doi.org/10.1016/j.lithos.2014.05.033

Gou, L.L., Zi, J.W., Dong, Y., Liu, X., Li, Z., Xu, X., Zhang, C., Liu, L., Long, X., and Zhao, Y., 2019. Timing of Two Separate Granulite-Facies Metamorphic Events in the Helanshan Complex, North China Craton: Constraints from Monazite and Zircon U-Pb Dating of Pelitic Granulites. Lithos, 350–351: 105216. https://doi.org/10.1016/j.lithos.2019.105216

Jiao, S.J., Fitzsimons, I.C.W., Zi, J.W., Evans, N.J., Mcdonald, B.J., and Guo, J.H., 2020. Texturally Controlled U–Th–Pb Monazite Geochronology Reveals Paleoproterozoic UHT Metamorphic Evolution in the Khondalite Belt, North China Craton. Journal of Petrology, 61: egaa023. https://doi.org/10.1093/petrology/egaa023

Zou, L., Huang, G.Y., Jiao, S.J., Prasanth, M.M., Liu, P.H., and Guo, J.H., 2026. The Paleoproterozoic Ultrahigh-Temperature Metamorphic Evolution of Alxa Block, North China Craton: Insights from Phase Equilibria Modeling, Garnet Trace Element Chemistry, and Monazite Geochronology. Geological Society of America Bulletin, 138: https://doi.org/10.1130/B38583.1

Minor comment 2.35

Fig S05c and g. Similar issue here - increasing Eu anomaly from Mnz-1 to Mnz-3 yet this is plotted inversely in (g).

Thank you for your comment. As mentioned in Minor comment 2.31, the previous discrepancy in observations was likely due to this error in the monazite label colors in the

figure. The issue has been corrected in the revised figure, which should now be accurate.

In this study, the REE distribution patterns of all monazite microdomains exhibit negative Eu anomalies. The Eu/Eu* values of these microdomains are all less than 1. The smaller the value, or the further it deviates from 1, the more pronounced the negative anomaly.

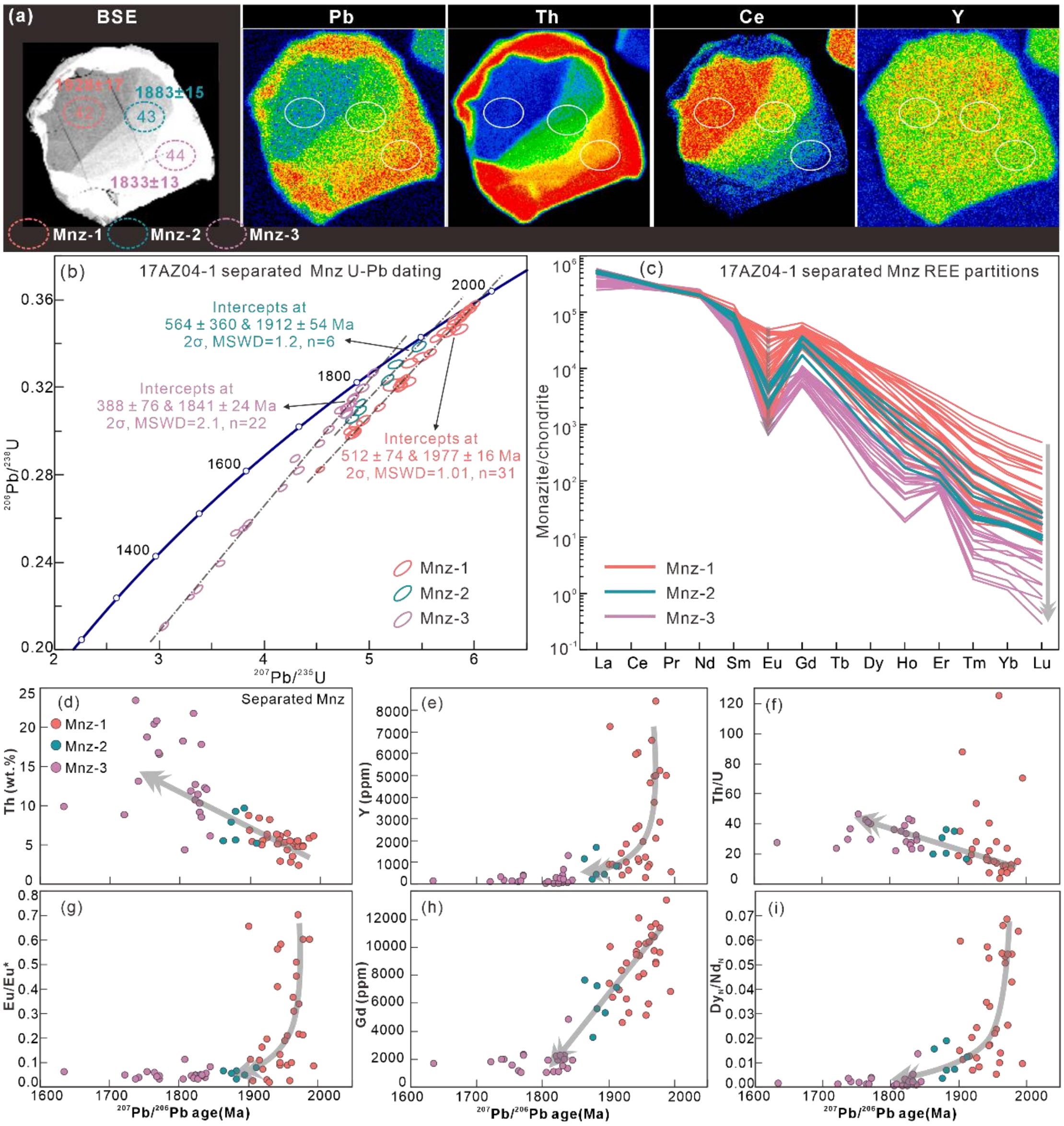

Minor comment 2.36

References. OK - this formatting error isn't helping...

We apologize for the formatting issues that caused the references in the Supplementary material to not display correctly. We have reformatted the references, and they are now visible.

Regards

Alan Collins

Reviewer #3 (Remarks to the Author):

The manuscript by Zou et al. uses new (monazite U-Pb and garnet Lu-Hf) and compiled data to study hot-orogens in the Paleoproterozoic. The text is well written, and the figures and data are of great quality.

Below are some comments:

Comment 3.1

(1) Fig. 1: Numbers plotted in the figure are not mentioned in the legend. What do the numbers 1, 2, 3, 19, etc. mean?

The numbers represent the orogen identifiers used for statistical purposes in this paper, and they correspond sequentially to the indices listed in the Supplementary Table 5. We have added a corresponding explanation in the caption of Figure 1 (Lines 805–807).

"The numbers in the figure represent the orogenic belt numbers compiled in this study, and each number corresponds to that in Supplementary Table 5."

Comment 3.2

(2) Lines 141 – 142: "Collision age at ca. 1.99 – 1.98 Ga progressing to peak conditions at ca. 1.96 – 1.95 Ga". I'm not sure where these ages came from, since they do not match the garnet Lu-Hf or monazite U-Pb ages.
I'm guessing 1.96 – 1.95 Ga is the age of zircons previously cited, but I'm not sure.
This part needs some rewriting to make this discussion and interpretation clearer and based on literature and/or the new data exposed in the text.

Thank you for your constructive comments. We have now explicitly linked the garnet Lu-Hf and monazite ages to specific collisional orogenic stages in the text (Lines 125 and 131). This reveals that the garnet core Lu-Hf age and the Mnz-1 U-Pb age (ca. 1.99–1.98 Ga) represent a time close to the initial collision.

As you pointed out, the age of the peak collisional stage (ca. 1.96–1.95 Ga) primarily cites previously reported data from the Khondalite Belt. Accordingly, we have revised the corresponding description. It now emphasizes that the results of this study, integrated with previous data, collectively reveal the collisional orogenic duration and tectonic evolutionary stages of the Khondalite Belt during the Paleoproterozoic (Lines 146–152).

"Collectively, combining our multi-chronometer framework and the previous metamorphic (a single UHT event[43,52]) and geochronological data, reveals a collisional orogenic duration of ca. 220 Myr for the western Khondalite Belt during the Palaeoproterozoic. Collision commenced at ca. 1.99–1.98 Ga (this study), progressed to peak collision by ca. 1.96–1.95 Ga[38,44,48,49], culminating in the thermal peak at ca. 1.92–1.91 Ga (e.g., ref.[45,50,53,54] and this study) and concluded with gradual cooling until ca. 1.8–1.77 Ga (e.g., ref.[43-45] and this study)."

Comment 3.3

(3) Fig. S6 / Line 193: Fig. 6 uses crust thickness based on Eu anomalies (Tang et al., 2021).
This is a problem that is well constrained in the literature now.

Eu anomalies are controlled by plagioclase proportion and are a function of pressure, temperature, rock composition, and changes in oxygen fugacity.
So now we know that Eu anomalies in zircon should not be used as a proxy for crustal thickness (Yakymchuk et al., 2023).
If the authors used this proxy to model the results shown in Figure 4 and Figure S07, they should acknowledge this important limitation. I personally believe that the authors should not use Eu anomalies to estimate crustal thickness and should not use these results for modelling. Using the Eu anomaly proxy could yield a wrong model, and, subsequently, all results and discussions will be questionable.
Yakymchuk, C., Holder, R. M., Kendrick, J., & Moyen, J. F. (2023). Europium anomalies in zircon: A signal of crustal depth?. Earth and Planetary Science Letters, 622, 118405. https://doi.org/10.1016/j.epsl.2023.118405

Thank you for your constructive comment. We recognize our oversight. In the new text, we have acknowledged the limitations of the crustal thickness model we employed (Lines 206–208). Our 1D modeling results indicate that radiogenic heat production, erosion rate, and mantle temperature are the primary controlling factors for orogenic duration and thermal state, while crustal thickness has a relatively minor influence on the simulation results and discussion.

"(we employed the model of Tang et al.[66] to calculate crustal thickness, although some researchers[67] also posed several limitations on this model, since the Eu anomaly is controlled by multiple complex factors)"

67 *Yakymchuk, C., Holder, R. M., Kendrick, J. & Moyen, J. F. Europium anomalies in zircon: A signal of crustal depth? Earth and Planetary Science Letters 622, 118405 (2023). https://doi.org/doi.org/10.1016/j.epsl.2023.118405*

Comment 3.4

(4) Line 234 – 238: Huang et al (2026) dated eclogite (low T/P) in the transition between the Mesoarchean and Neoarchean - 1.5 – 2.5 GPa and temperatures of 580 – 660 °C (low T/P conditions of 230 – 440 °C/GPa).
Also, there are the classical Brown and Johnson graphs (and other authors as well; https://doi.org/10.1144/jgs2024-091?ref=pdf&rel=cite-as&jav=VoR; https://doi.org/10.1016/j.precamres.2023.107195; https://doi.org/10.1038/s41598-024-56691-1) compiling high, moderate, and low T/P data from the metamorphic record worldwide. They have shown a series of low T/P eclogites in the Paleoproterozoic and even in the Archean.
Also, potential evidence of low T/P metamorphism before ca. 2.5 Ga can be also found in North China (Ning et al., 2022), and Bundelkhand Craton (India; Saha et al., 2011)
All these information should be acknowledged in the manuscript. Will this change the interpretations of this manuscript?

Thank you for your constructive comment. It was an oversight on our part to have omitted the information regarding these Archaean HP and eclogite-facies metamorphic events. We have now added a discussion in the manuscript (Lines 254–260), incorporating those well-documented cases. However, the low d*T*/d*P* metamorphism before ca. 2.5 Ga found in North China (Ning et al., 2022) remains subject to considerable debate regarding both its *P-T* conditions (Zou et al., 2022) and metamorphic timing (Yan et al., 2026); therefore, we decided not to include it in this study.

It is noteworthy that all currently reported Archean low d*T*/d*P* metamorphic rocks are metapelites rather than eclogites. These lower-density HP metapelites facilitate their exhumation to the surface, standing in stark contrast to denser eclogites, which promote accelerated plate motion. The first reported occurrences of these low d*T*/d*P* rocks in the Archaean may provide a record of the transition from a pre-plate tectonic regime towards plate tectonics (Huang et al., 2026). However, metamorphism during the Neoarchaean remained characterized by high to moderate d*T*/d*P* metamorphism (Brown et al., 2020). Combined with extensive broad structural deformation domains and prolonged high thermal states, these collectively imply relatively low plateness and surface mobility consistent with inferred plate-velocity proxies, revealing a relatively sluggish tectonic regime.

(Lines 254–260) "It is noteworthy that the first appearance of HP to eclogite-facies metapelites (ref.[61,72]) under low d*T*/d*P* ratios during this period may record the transition from some pre-plate tectonic regime to the plate tectonics[1,3,10,61]. However, widespread high to moderate d*T*/d*P* metamorphism[55], combined with extensive broad deformation domains and prolonged high-thermal states, implies relatively low plateness and surface mobility[2] consistent with inferred plate-velocity proxies[73] (Fig. 5b), revealing a relatively sluggish tectonic regime[10]."

Ning, W., Kusky, T., Wang, L., & Huang, B. (2022). Archean eclogite-facies oceanic crust indicates modern-style plate tectonics. Proceedings of the National Academy of Sciences, 119(15), e2117529119.

Zou, Y., Chu, X., Wu, J., & Zhao, L. (2022). No evidence for Archean eclogite-facies metamorphism. Proceedings of the National Academy of Sciences, 119(39), e2208090119.

Yan, Y. Q., Wang, H., Yang, J. H., Wu, S. T., & Ran, J. (2026). Reevaluating Archean eclogite-facies metamorphism in the North China Craton. Geology. https://doi.org/10.1130/G54220.1

Huang, M. et al. Eclogite-facies metamorphism of continental crust at the Mesoarchean-to-Neoarchean transition. Geology (2026). https://doi.org/10.1130/g53819.1

Brown, M., Johnson, T., Gardiner, N.J. (2020). Plate Tectonics and the Archean Earth. Annual Review of Earth and Planetary Sciences. 48(1): 291-320.

Comment 3.5

(5) Line 286: "Alternating tectonic regime evolution". It is not clear why the authors concluded this. The authors did not mention low T/P (eclogite) data in the Archean/Paleoproterozoic to support these alternating tectonic regimes.

I believe this interpretation is based on the existence of low T/P (i.e., plate tectonics / mobile-lid) between periods of hot orogenesis. But this is not clear through the text: several authors dated low T/P rocks in the Archean/Paleoproterozoic.

As also noted by Reviewer #1, we neglected to provide a clearer definition and elaboration of the concept of an "alternating tectonic regime." This oversight made it difficult for readers to fully grasp the central thesis of the paper. Accordingly, we have now added a clearer definition and discussion of this concept into the revised manuscript (Lines 234–250).

“This alternating thermal evolution is likely a response to changing lithospheric coupling parameters such as the plateness and mobility (e.g., Fig. 5), which are an overall expression of the tectonic regimes under which the Earth has operated through its history[1]. Plateness refers to the degree of rigidity of tectonic plates[1,2]. Plate tectonics is characterized by high degrees of plateness (i.e., rigid plates with deformation focused at plate boundaries). In contrast, non-plate tectonic mobile lid regimes (e.g., sluggish lid) may involve broader zones of plate deformation, and hence lower plateness. Sluggish lid regimes will be facilitated by magmatism and UHT metamorphism, which will decrease, at least locally, plate rigidity[1,69]. This will also likely lead to non-uniform rates of plate motion. Thus, the non-monotonic evolution of orogenic belts can be viewed in response to changing plateness and mobility, leading to an alternation between sluggish and active plate-tectonic states. The Neoarchean is characterized by likely sluggish lid setting progressing to an active plate tectonic phase in the Paleoproterozoic, then a return to sluggishness in the Mesoproterozoic, which corresponds with Earth’s Middle Age[70], and finally a transition to contemporary active plate tectonics from the Neoproterozoic into the Phanerozoic.”

The term “alternating tectonic regimes” proposed here describes Earth’s non-monotonic progression through Neoarchean to Phanerozoic supercontinent cycles, characterized by alternating sluggish lid and plate tectonic states primarily defined by lithospheric plateness and mobility. This study utilizes orogenic duration and thermal state as primary proxies for these regimes. During the Precambrian, hot orogens prevailed, exhibiting higher thermal states and longer durations than their Phanerozoic counterparts. Specifically, Neoarchean orogenic events potentially exceeded 300 Myr with UHT metamorphism lasting over 100 Myr, signifying significantly lower plateness and mobility. While the Paleoproterozoic saw a reduction in these timescales and the appearance of cold orogens, reflecting increased mobility, the Mesoproterozoic reverted to Neoarchean-like characteristics with prolonged durations and fewer cold orogens. Finally, the Neoproterozoic–Phanerozoic transition marks a definitive shift to high plateness and mobility, evidenced by rapidly decreasing durations and thermal states. These trends are independently corroborated by plate velocity records, deformation styles, and the existence of low dT/dP metamorphism during Early Precambrian hot orogeny, as you noted. Therefore, we propose that these alternating evolutionary indicators may reveal an "alternating tectonic regimes."
